# IL-10 attenuates metabolic dysfunction–associated steatotic liver disease via modulation of hepatic responses to lipotoxicity

**DOI:** 10.1172/jci.insight.200231

**Published:** 2026-04-23

**Authors:** Akira Kado, Kazuya Okushin, Takeya Tsutsumi, Toshiyuki Kishida, Kazuhiko Ikeuchi, Hiroshi Yotsuyanagi, Kyoji Moriya, Kazuhiko Koike, Mitsuhiro Fujishiro

**Affiliations:** 1 Department of Gastroenterology, Graduate School of Medicine;; 2 Division for Health Service Promotion;; 3 Department of Infection Control and Prevention, Graduate School of Medicine;; 4 Department of Infectious Diseases, Graduate School of Medicine; and; 5 Division of Infectious Diseases, Advanced Clinical Research Center, Institute of Medical Science, The University of Tokyo, Tokyo, Japan.; 6 Division of Infection Control and Prevention, Education Research Center, Tokyo Health Care University, Higashigotanda, Shinagawa-ku, Tokyo, Japan.; 7 Department of Gastroenterology, Kanto Central Hospital, Kamiyoga, Setagaya-ku, Tokyo, Japan.

**Keywords:** Hepatology, Immunology, Metabolism, Cell stress, Hepatitis, Homeostasis

## Abstract

Lipotoxicity associated with metabolic dysfunction–associated steatotic liver disease (MASLD) causes dysregulated fatty acid (FA) and glucose metabolism, inducing cellular energy imbalance, oxidative stress (OS), and hepatocellular injury. IL-10 is altered in MASLD, including increased IL-10 transcripts in peripheral immune cells; however, its role in hepatic responses to lipotoxic stress remains unclear. We evaluated whether IL-10 treatment attenuates lipotoxic injury and MASLD-related phenotypes in vivo and in vitro to reveal MASLD treatment strategies. As MASLD models, mice fed a high-fat diet and in vitro normal human hepatocytes under palmitic acid exposure were treated with IL-10, along with confirmatory experiments in HepG2 cells. We assessed FA and glucose metabolism, OS, and apoptosis with histological changes and mechanisms related to hepatocellular viability/metabolic activity and stress-responsive survival signaling in vitro. IL-10 modulated FA synthesis and β-oxidation, reducing lipid accumulation, and IL-10 altered glucose metabolic pathways, consistent with improved glucose handling under lipotoxic stress. Furthermore, IL-10 reduced OS and cell death markers while enhancing antioxidant responses, consistent with hepatocellular protection. These data suggest that IL-10 attenuates lipotoxic injury by modulating hepatic response pathways, thereby improving MASLD-related phenotypes, and support the potential of IL-10 as a therapeutic target for MASLD.

## Abstract 2

In fatty liver disease models, interleukin-10 reduced liver fat, stress, and cell death, suggesting a promising new treatment strategy.

## Graphical abstract

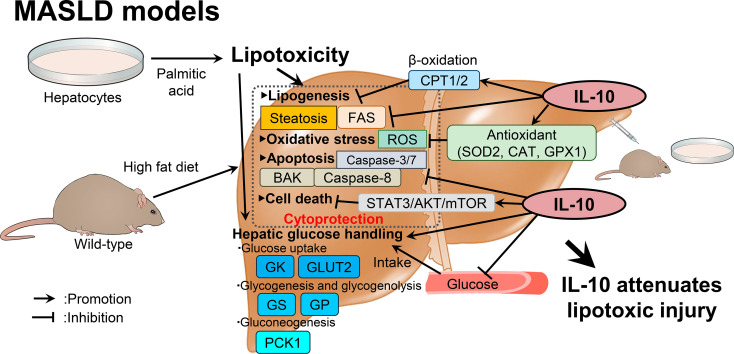

## Introduction

Metabolic dysfunction–associated steatotic liver disease (MASLD) is the most common chronic liver disease worldwide, closely linked to metabolic syndrome. Untreated MASLD increases the risk of metabolic complications, including diabetes mellitus, dyslipidemia, and liver cancer (1). Hepatic lipotoxicity is a major concern (2), frequently arising from obesity and excessive fatty acid (FA) accumulation in non-adipose tissues, leading to cell death and metabolic dysfunction in the liver (3), including disrupted FA and glucose metabolism (4). Lipotoxicity contributes to diabetes mellitus by inducing insulin resistance (IR) and pancreatic β cell dysfunction, ultimately reducing insulin secretion (5). Among saturated FAs, palmitic acid (PA), which is elevated in patients with MASLD, exacerbates hepatic lipotoxicity and worsens metabolic damage (6). Additionally, hepatic lipotoxicity induces mitochondrial dysfunction, apoptosis, and IR/diabetes mellitus via oxidative stress (OS) stemming from impaired β-oxidation and ROS accumulation in the liver. Although ROS are also generated by alcohol, drugs, and exercise (2, 3, 7), their excess contributes to disease progression. Lipotoxicity has been associated with multiple chronic conditions beyond MASLD (8, 9), underscoring the clinical importance of strategies that attenuate lipotoxic injury.

Homeostasis is a physiological mechanism that maintains a stable internal environment, including in the liver (10). Defining endogenous lipotoxic stress response pathways may help identify targets that limit hepatocellular damage. Although several mechanisms partially regulating this pathosis have been elucidated (11), the integrative mediators that coordinate these responses in MASLD remain incompletely understood. Among cytokines and chemokines, we focused on IL-10, an immunoregulatory cytokine that suppresses excessive inflammation (including hepatitis) and has been implicated in immune homeostatic regulation (12–14). IL-10 also influences cell survival and has been reported to modulate autophagy (15). In MASLD, IL-10 gene expression is upregulated in peripheral immune cells, and peripheral immune alterations have been linked to disease severity and inflammatory phenotypes (16–18). Hepatic and circulating IL-10 measurements in humans vary across cohorts, highlighting context dependence (16, 19). These observations suggest that IL-10 may modulate MASLD; however, how IL-10 influences hepatic responses to lipotoxic injury and related metabolic dysfunction remains unclear. Although endogenous IL-10 may be induced in MASLD, these responses may be insufficient in magnitude or timing to counter sustained lipotoxic stress, providing a rationale for therapeutic supplementation.

Here, we hypothesized that IL-10 induces hepatic homeostatic response programs and that augmenting IL-10 signaling can attenuate lipotoxic injury in MASLD models. We therapeutically evaluated the effects of IL-10 treatment on lipotoxicity-associated metabolic dysfunction, OS, and cell death in vivo in mice fed a high-fat diet (HFD) and in vitro in normal human hepatocytes (NHHs) under PA exposure, with confirmatory experiments in HepG2 cells. We focused on lipid and glucose metabolism together with OS and apoptosis to clarify how IL-10 treatment reshapes hepatic responses to lipotoxic stress. These findings support IL-10 as a potential modulator of lipotoxicity-related pathosis and a therapeutic target for MASLD.

## Results

### IL-10 regulates hepatic lipid accumulation.

**Figure 1:**
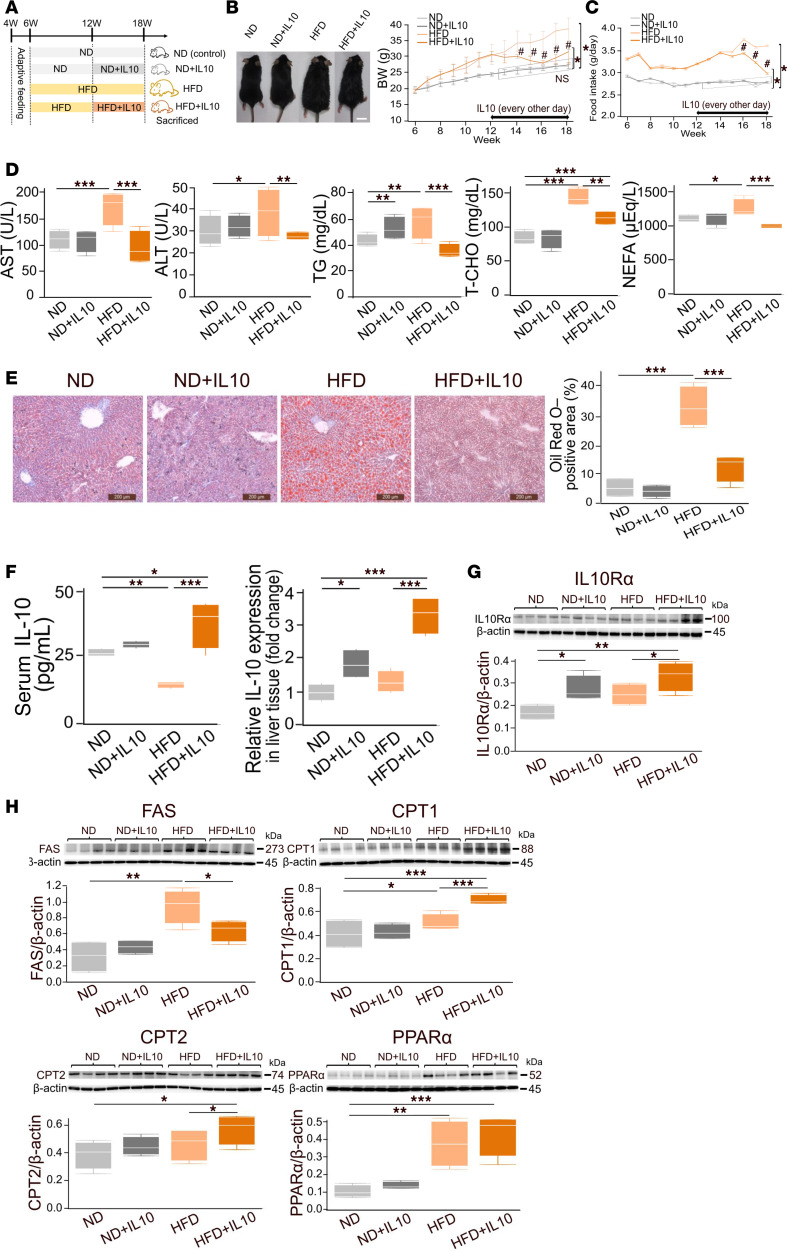
IL-10 suppresses hepatic lipid accumulation in HFD-fed mice. (**A**) Four experimental groups (ND, ND+IL-10, HFD, and HFD+IL-10). (**B**) Representative images of mice from 4 experimental groups before euthanasia and body weights in each group. *P < 0.05 versus ND-fed mice (control); ^#^*P* < 0.05 versus HFD-fed mice. Scale bar: 2 cm. (**C**) Food intake in each group. Food intake is monitored throughout the treatment period and expressed as mean (g/d) per mouse. (**D**) Fasting serum levels of AST, ALT, TG, T-CHO, and NEFA at euthanasia. (**E**) Oil Red O staining of the liver tissue sections and Oil Red O–positive area (percentage) in each view. Scale bar: 200 μm. The analysis was performed 4 times per group using different views. (**F**) Serum IL-10 levels at euthanization; quantitative hepatic IL-10 levels and relative fold-changes compared with control. (**G** and **H**) Immunoblot analysis of IL-10Rα, FAS, CPT1, CPT2, and PPARα. Protein band intensities are normalized to β-actin and expressed as ratios. Box-and-whisker plots show the median, IQR, and full data range. One-way ANOVA followed by Tukey’s multiple-comparison test; *n* = 4, *P < 0.05, **P < 0.01, ***P < 0.001. CPT, carnitine palmitoyltransferase; FAS, fatty acid synthase; HFD, high-fat diet; ND, normal diet.

**Figure 2:**
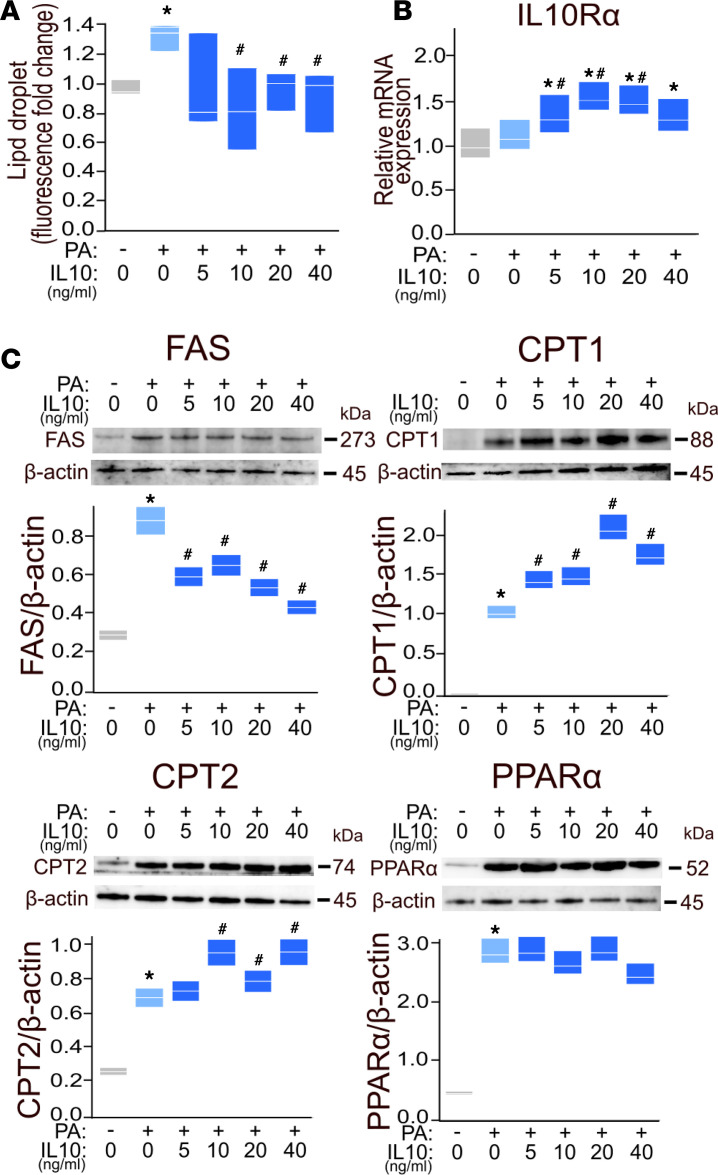
IL-10 suppresses hepatic lipid accumulation in NHHs. (**A**) NHHs were incubated with PA (0, 5, 10, 20, and 40 ng/mL) or with DMEM alone (control) for 24 hours. Quantitative lipid droplet accumulation and relative fold-changes compared with control. (**B**) mRNA expression levels in *IL-10Rα* and relative fold-changes compared with control normalized to *GAPDH*. (**C**) Immunoblot analysis of FAS, CPT1, CPT2, and PPARα. Protein band intensities are normalized to β-actin and expressed as ratios. Box-and-whisker plots show the median, IQR, and full data range. One-way ANOVA followed by Tukey’s multiple-comparison test; *n* = 3, *P < 0.05 versus non-PA and IL-10 (0 ng/mL) (control); ^#^*P* < 0.05 versus PA and IL-10 (0 ng/mL). CPT, carnitine palmitoyltransferase; FAS, fatty acid synthase; NHHs, normal human hepatocytes.

We assessed the effects of IL-10 on hepatic lipid accumulation in vivo using HFD-fed mice (Figure 1A) and in vitro using PA-exposed hepatocyte models. Body weight (BW) was increased in HFD-fed mice compared with controls fed a normal diet (ND), but among HFD-fed mice, it was decreased in the IL-10 treatment group (Figure 1B). To evaluate whether the attenuation of BW gain could be explained by altered caloric consumption, food intake was monitored and did not differ between ND and ND+IL-10 groups, whereas HFD feeding increased food intake compared with ND. In HFD-fed mice, IL-10 treatment was associated with a reduction in food intake after treatment initiation (Figure 1C). Serum aspartate aminotransferase (AST), alanine aminotransferase (ALT), triglyceride (TG), total cholesterol (T-CHO), and non-esterified FA (NEFA) levels were increased in HFD-fed mice, with a reduction after IL-10 treatment (Figure 1D). Hepatic lipid droplet accumulation was visually and quantitatively assessed, revealing that IL-10 reduced lipid droplet accumulation induced by the HFD (Figure 1E). To assess in vivo IL-10–induced exposure, we quantified circulating and hepatic IL-10 levels when the mice were euthanized. Among HFD-fed mice, IL-10 increased serum and hepatic IL-10 levels compared with nontreatment, whereas these levels were not increased in HFD-fed mice compared with ND-fed mice (Figure 1F). IL-10 increased IL-10Rα protein expression among ND- and HFD-fed mice (Figure 1G). To further investigate the hepatic response to IL-10, IHC staining for IL-10Rα in liver tissues was performed (Supplemental Figure 1; supplemental material available online with this article; https://doi.org/10.1172/jci.insight.200231DS1). This staining showed increased IL-10Rα immunoreactivity in non-parenchymal regions in HFD-fed mice compared with ND-fed mice, while intracellular staining in hepatocytes was more prominent after IL-10 treatment. To further characterize lipid metabolic pathways, we assessed FA-related enzymes: fatty acid synthase (FAS), FA β-oxidation (carnitine palmitoyltransferase [CPT] 1 and CPT2), and PPARα (20). Although HFD increased FAS, CPT1, and PPARα levels, IL-10 reduced FAS, increased CPT1 and CPT2, and interestingly had no effect on PPARα (Figure 1H). To assess whether these in vivo findings were recapitulated in hepatocytes under lipotoxic stress, we examined lipid accumulation in NHHs after PA exposure. IL-10 reduced PA-induced lipid droplet accumulation compared with controls (Figure 2A), with similar trends observed in HepG2 cells (Supplemental Figure 2, A–C). Then, changes in mRNA expression of IL-10Rα showed almost the same results as in liver tissues (Figure 2B). FA-related enzyme expression in vitro mirrored in vivo findings (Figure 2C).

Furthermore, the same analysis was performed after short-term IL-10 treatment to evaluate IL-10 temporal effects. Using the same mouse-rearing method, IL-10 treatment duration was reduced from 6 weeks to 1 week (Supplemental Figure 3A). In the short-term, BW gain was reduced (Supplemental Figure 3B), and food intake showed a similar directional change, with a trend toward reduction in HFD-fed mice after IL-10 initiation; however, the magnitude was smaller and statistically significant (Supplemental Figure 3C). Short-term IL-10 treatment also reduced serum AST, ALT, TG, T-CHO, and NEFA levels and hepatic lipid droplet accumulation (Supplemental Figure 3, D and E). Among HFD-fed mice, IL-10 increased IL-10Rα protein expression and serum and hepatic IL-10 levels compared with nontreatment (Supplemental Figure 4, A–C). Regarding FA metabolism, the results resembled those of long-term IL-10 treatment; however, CPT1 and CPT2 levels remained unchanged in HFD-fed mice receiving short-term IL-10 treatment (Supplemental Figure 4D). These findings suggest that IL-10 reduced hepatic lipid accumulation in both short- and long-term settings, with broader changes in β-oxidation enzymes observed after prolonged treatment.

### IL-10 attenuates pathological steatotic change.

**Figure 3:**
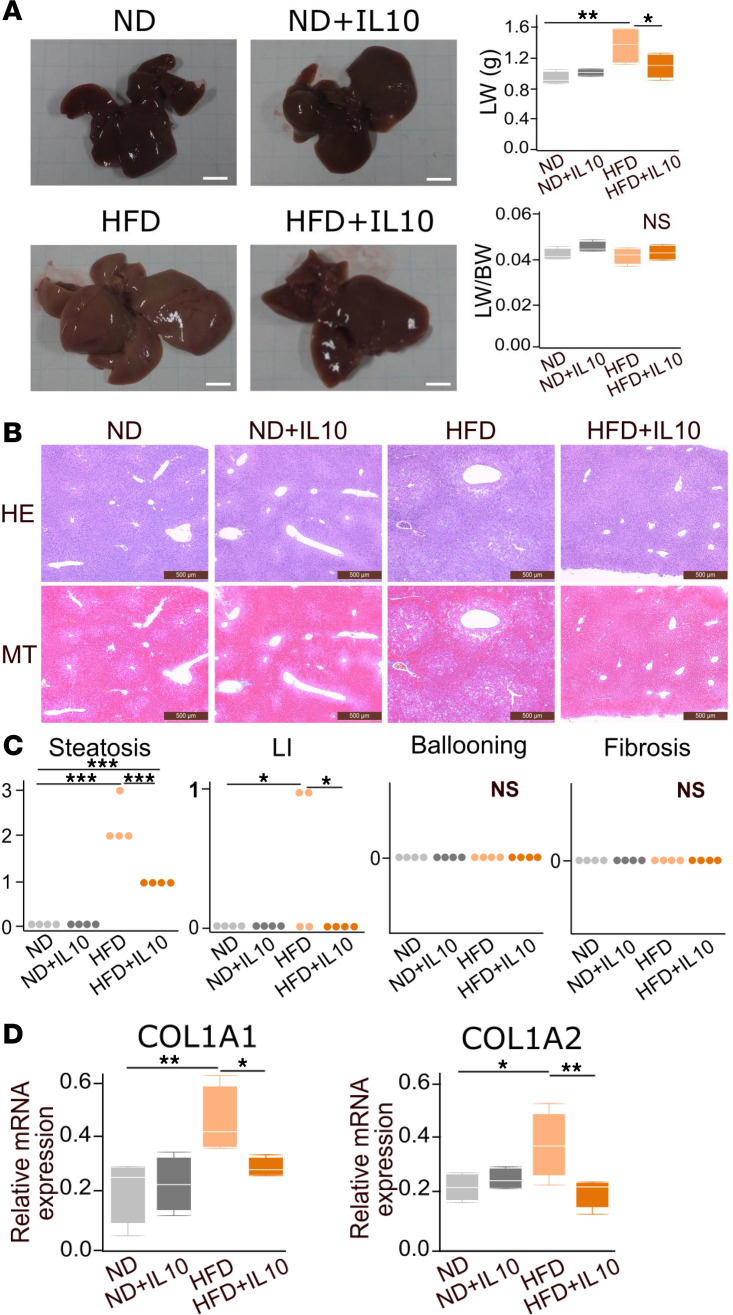
Long-term IL-10 treatment provides hepatic histological improvement in HFD-fed mice. (**A**) Representative images of mouse livers from 4 experimental groups, LWs in each group, and the LW/BW ratio. Scale bar: 5 mm. (**B**) H&E and Masson’s trichrome staining to evaluate liver histology. Scale bar: 500 μm. (**C**) Hepatic steatosis, lobular inflammation, hepatocyte ballooning, and fibrosis staging. (**D**) mRNA expression levels in *COL1A1* and *COL1A2* were examined and normalized to *GAPDH*. All data are presented as box-and-whisker plots showing the median, IQR, and full data range. One-way ANOVA followed by Tukey’s multiple-comparison test; *n* = 4, *P < 0.05, **P < 0.01, ***P < 0.001. BW, body weight; HFD, high-fat diet; LW, liver weight; ND, normal diet.

We assessed liver weight (LW), the LW/BW ratio, and H&E and Masson’s trichrome staining in liver pathology and quantified hepatic steatosis, lobular inflammation, hepatocyte ballooning, and fibrosis. IL-10 reduced LW, which had been increased by the HFD, compared with controls, and the LW/BW ratio remained unchanged (Figure 3A). The treatment also reduced HFD-induced steatosis and lobular inflammation but did not alter hepatocyte ballooning or fibrosis (Figure 3, B and C). Notably, IL-10 decreased mRNA expression levels of *COL1A1* and *COl1A2* fibrosis markers (21), which were elevated by the HFD (Figure 3D). To further assess the activation of hepatic stellate cells involved in liver inflammation and fibrosis (22), we performed immunostaining for α–smooth muscle actin (α-SMA) and IL-10Rα (Supplemental Figure 5); α-SMA signal was increased in HFD-fed mice with a reduction after IL-10 treatment. In short-term IL-10 treatment, IL-10 did not alter LW or *COL1A1* and *COL1A2* expression in HFD-fed mice (Supplemental Figure 6, A and D). Hepatic pathological assessments largely mirrored findings from long-term IL-10 treatment (Supplemental Figure 6, B and C). These findings suggest that IL-10 improves steatosis and lobular inflammation and reduces fibrogenic gene expression, although hepatocyte ballooning and fibrosis scores remained unchanged, consistent with relatively early-stage HFD phenotype and the limited dynamic range within the study duration.

### IL-10 modulates hepatic glucose content and glucose-related pathways.

**Figure 4:**
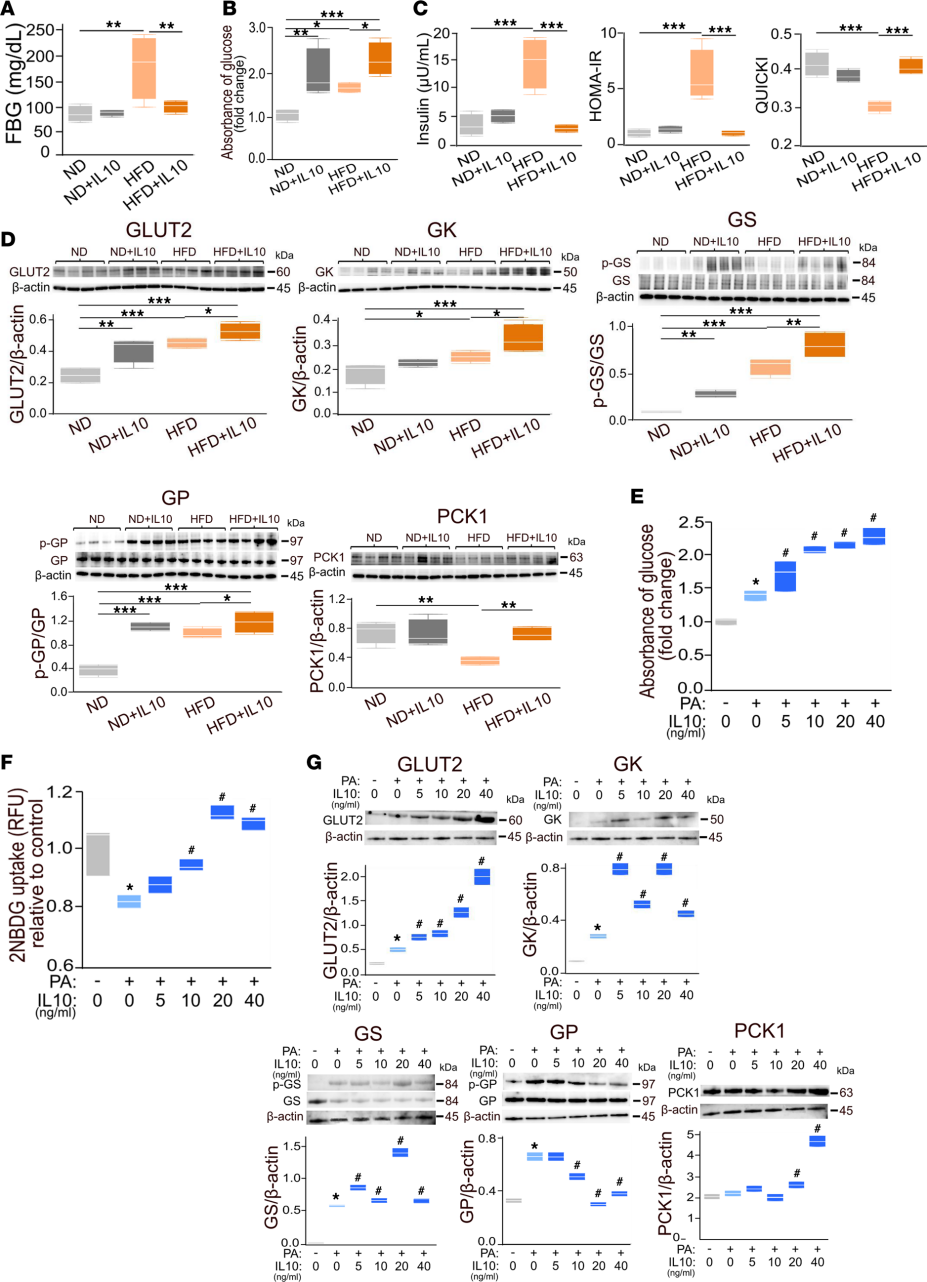
IL-10 promotes hepatic glucose content in HFD-fed mice and normal human hepatocytes. HFD-fed mice (**A**–**D**). (**A**) FBG levels at time of euthanasia. (**B**) Quantification of hepatic glucose content, expressed as relative fold-changes compared with the control. (**C**) Fasting serum insulin levels, HOMA-IR, and QUICKI in each group. QUICKI is calculated from fasting glucose (G0) and insulin (I0) as QUICKI = 1/[log(I0) + log(G0)]. (**D**) Immunoblot analysis of GLUT2, GK, GS, GP, and PCK1. Protein band intensities are normalized to β-actin and expressed as ratios. Box-and-whisker plots show the median, IQR, and full data range. One-way ANOVA followed by Tukey’s multiple-comparison test; *n* = 4, *P < 0.05, **P < 0.01, ***P < 0.001. Normal human hepatocytes (NHHs) (**E**–**G**). (**E**) Quantitative cellular glucose content in NHHs and relative fold-changes compared with the control. (**F**) 2-NBDG uptake in NHHs under palmitate ± IL-10. Fluorescence (RFU) is shown relative to control. (**G**) Immunoblot analysis of GLUT2, GK, GS, GP, and PCK1 in NHHs. β-Actin loading control for GK was obtained from the same gel with GLUT2. Box-and-whisker plots show the median, IQR, and full data range. One-way ANOVA followed by Tukey’s multiple-comparison test; *n* = 3, *P < 0.05 versus non-PA and IL-10 (0 ng/mL) (control); ^#^*P* < 0.05 versus PA and IL-10 0 ng/mL. GLUT2, glucose transporter 2; GK, glucokinase; GP, glycogen phosphorylase; GS, glycogen synthase; HFD, high-fat diet; HOMA-IR, homeostatic model assessment for IR; IR, insulin resistance; ND, normal diet; PA, palmitic acid; PCK1, phosphoenolpyruvate carboxykinase 1.

We next examined systemic and hepatic glucose-related phenotypes. Blood biochemistry analysis showed that fasting blood glucose (FBG) levels were elevated in HFD-fed mice compared with controls, but IL-10 reduced FBG in HFD-fed mice (Figure 4A). Hepatic glucose content quantification revealed that IL-10 further increased hepatic glucose content induced by the HFD compared with nontreatment (Figure 4B). IL-10 also reduced serum insulin levels and homeostatic model assessment for IR (HOMA-IR), both of which were elevated by the HFD (Figure 4C). To strengthen fasting-based assessment of insulin sensitivity beyond HOMA-IR, we calculated the quantitative insulin sensitivity check index (QUICKI) from fasting glucose and insulin values (QUICKI = 1/[log(I0) + log(G0)]) (23). IL-10 also increased QUICKI, which were decreased by HFD; these results suggest that IL-10 suppresses IR. We also evaluated the effects of IL-10 on enzymes related to glucose metabolism, specifically glucose uptake (glucose transporter 2 [GLUT2]), glycolysis (glucokinase [GK]), glycogen synthesis (glycogen synthase [GS]), and degradation (glycogen phosphorylase [GP]), and gluconeogenesis-related enzyme expression (phosphoenolpyruvate carboxykinase 1 [PCK1]). Given that glycogen enzymes are primarily regulated by phosphorylation, we quantified inhibitory p-GS (Ser641) and activating p-GP (Ser15) in addition to total proteins.

HFD increased GLUT2 and GK and elevated basal p-GS/GS and p-GP/GP ratios, consistent with impaired insulin signaling that sustains GS phosphorylation and preserved glycogenolytic drive (24). Among HFD-fed mice, IL-10 increased GLUT2 and GK and restored PCK1, while further increasing basal p-GS/GS and p-GP/GP ratios (Figure 4D). To assess the hepatic glucose accumulation of IL-10 in hepatocellular lipotoxicity, we evaluated glucose content in NHHs. IL-10 further enhanced glucose accumulation after PA exposure compared with controls (Figure 4E). A 2-NBDG uptake assay showed that IL-10 restored hepatocellular glucose uptake that had been reduced under PA-induced lipotoxicity (Figure 4F). In PA-treated NHHs, IL-10 increased GLUT2/GK/GS (and related proteins) but decreased the basal p-GP/GP ratio (Figure 4G), suggesting an acute shift toward glycogen preservation under lipotoxic stress.

Furthermore, short-term IL-10 reduced serum FBG levels (Supplemental Figure 7A) and reduced HFD-induced IR compared with controls (Supplemental Figure 7B). However, unlike long-term treatment, it did not increase hepatic glucose content or alter GK and basal p-GS/GS and p-GP/GP ratios in HFD-fed mice (Supplemental Figure 7, C and D). These findings suggest that IL-10 improves systemic glycemia/IR in the short term, whereas prolonged IL-10 treatment is associated with measurable remodeling of hepatic glucose handling.

### IL-10 regulates hepatic oxidative stress.

**Figure 5:**
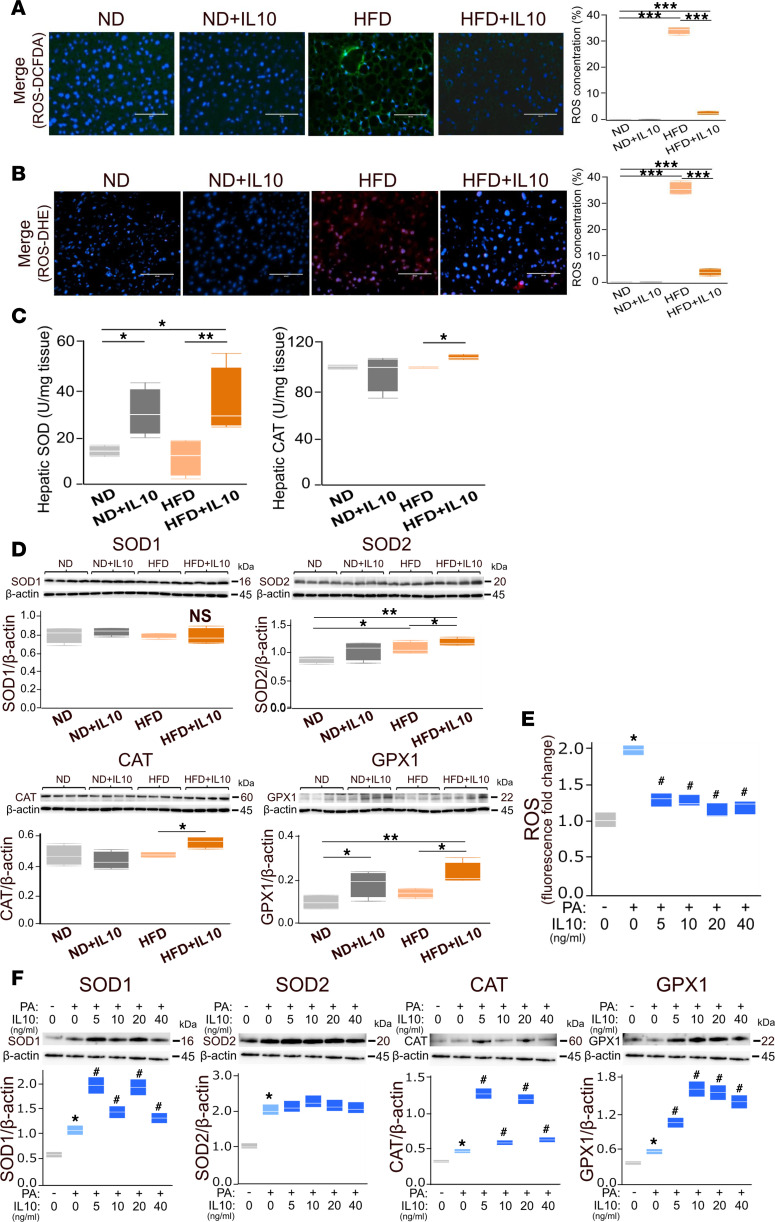
IL-10 suppresses hepatic oxidative stress in HFD-fed mice and normal human hepatocytes. HFD-fed mice (**A**–**D**). (**A**) Mean fluorescence intensity (MFI) of the merged channel, a pseudo-colored overlay of images from the GFP (green, DCFDA) and DAPI (blue) channels, showing liver tissues from 4 experimental groups. ROS localization is shown with green fluorescence. Scale bar: 50 μm. Quantitative hepatic ROS levels and the ROS-positive area (percentage) in each view. The analysis was performed 4 times per group using different views. (**B**) MFI of the merged channel from the RFP (red, DHE) and DAPI (blue) channels, showing liver tissues from 4 experimental groups. Nuclear ROS localization is shown with red fluorescence. Scale bar: 50 μm. Quantitative evaluation of ROS is as described above. (**C**) Hepatic total SOD and catalase activities measured in liver homogenates (normalized to tissue/protein as indicated). Data are expressed as U/mg tissue. (**D**) Immunoblot analysis of SOD1, SOD2, CAT, and GPX. Protein band intensities are normalized to β-actin and expressed as ratios. Box-and-whisker plots show the median, IQR, and full data range. One-way ANOVA followed by Tukey’s multiple-comparison test; *n* = 4, *P < 0.05, **P < 0.01, ***P < 0.001. Normal human hepatocytes (NHHs) (**E** and **F**). (**E**) Quantitative intracellular ROS accumulation in NHHs and relative fold-changes compared with the control. (**F**) Immunoblot analysis of SOD1, SOD2, CAT, and GPX1 in NHHs. β-Actin loading controls for SOD2, CAT, and GPX1 were obtained from the same gel with SOD1. Box-and-whisker plots show the median, IQR, and full data range. One-way ANOVA followed by Tukey’s multiple-comparison test; *n* = 3, *P < 0.05 versus non-PA and IL-10 (0 ng/mL) (control); ^#^*P* < 0.05 versus PA and IL-10 (0 ng/mL). CAT, catalase; GPX, glutathione peroxidase; HFD, high-fat diet; ND, normal diet; SOD, superoxide dismutase.

We next evaluated OS and antioxidant responses after IL-10 treatment. In the long-term, the degree of ROS in liver tissues was visually and quantitatively evaluated, revealing that IL-10 reduced hepatic ROS induced by the HFD compared with controls (Figure 5A). Consistent with DCFDA-based ROS reduction, dihydroethidium (DHE) staining revealed that IL-10 suppressed nuclear superoxide accumulation in hepatocytes of HFD-fed mice (Figure 5B). To substantiate enhanced antioxidant defense beyond protein abundance, we directly measured hepatic superoxide dismutase (SOD) and catalase (CAT) activities (Figure 5C), which were not markedly changed by HFD alone but were increased by IL-10 in HFD-fed mice. We examined the protein expression of antioxidant enzymes: SOD1, SOD2, CAT, and glutathione peroxidase 1 (GPX1) (7), and only SOD2 levels were increased in HFD-fed mice compared with controls (Figure 5D). IL-10 further increased SOD2, CAT, and GPX1 levels in HFD-fed mice, whereas SOD1 was not markedly altered, suggesting preferential engagement of mitochondrial antioxidant defenses under chronic diet-induced lipotoxic stress. To evaluate hepatocellular OS under lipotoxic conditions, we quantified ROS levels in NHHs, finding that IL-10 reduced PA-induced ROS accumulation compared with controls (Figure 5E). Similar results were visually and quantitatively observed in HepG2 cells (Supplemental Figure 8, A and B). Regarding the effect of IL-10 on antioxidant enzymes, their expression was increased by PA exposure compared with controls. IL-10 further upregulated SOD1 and GPX1, and CAT expression increased in treatment with some concentrations, whereas SOD2 was not significantly changed (Figure 5F), indicating a context-dependent antioxidant program in isolated hepatocytes.

Furthermore, short-term IL-10 treatment similarly reduced hepatic ROS induced by the HFD compared with controls (Supplemental Figure 8, C and D). Hepatic SOD activity was increased by short-term IL-10 treatment (Supplemental Figure 8E), whereas CAT activity showed smaller or nonsignificant changes. Regarding antioxidant activity, in HFD-fed mice, only SOD2 levels were increased with short-term IL-10 treatment (Supplemental Figure 8F). Overall, IL-10 reduced hepatic OS in long- and short-time regimens, with broader antioxidant upregulation observed after prolonged treatment.

### IL-10 attenuates hepatic apoptosis.

**Figure 6:**
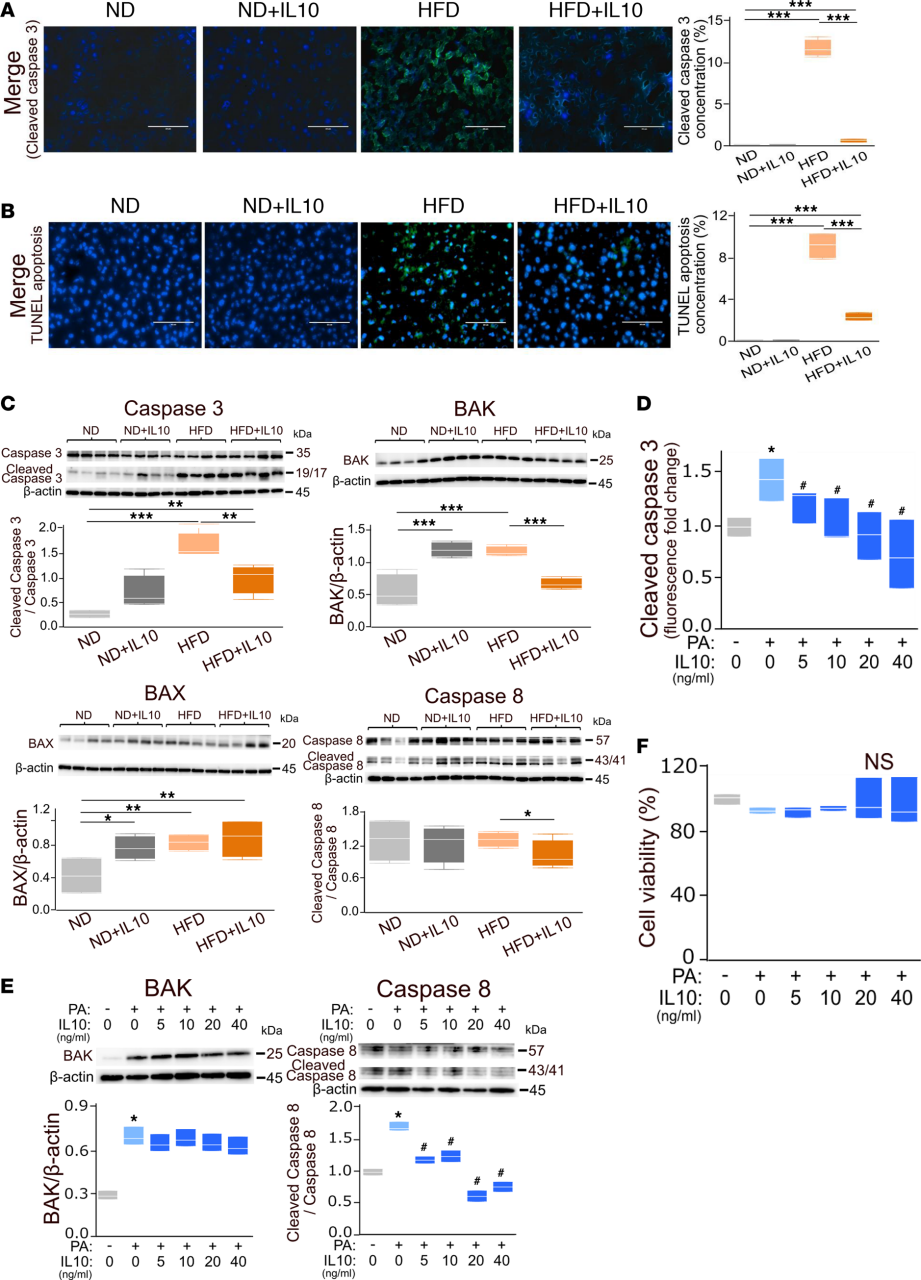
IL-10 suppresses hepatic apoptosis in HFD-fed mice and normal human hepatocytes. (**A**–**C**) HFD-fed mice. (**A**) MFI of the merged channel, a pseudo-colored overlay of images from the GFP (green, cleaved caspase-3) and DAPI (blue) channels, showing liver tissues from 4 experimental groups. Caspase-3 localization is shown with green fluorescence. Scale bar: 50 μm. Quantitative hepatic caspase-3/7 activity and the caspase-3/7–positive area (percentage) in each view. The analysis was performed 4 times per group using different views. (**B**) MFI of the merged channel from the GFP (green, TUNEL) and DAPI (blue) channels, showing liver tissues from 4 experimental groups. Nuclear DNA fragmentation as a marker of late-stage apoptosis is shown with green fluorescence. Scale bar: 50 μm. Quantitative evaluation of apoptosis is as described above. (**C**) Immunoblot analysis of BAK and BAX expression, along with the cleaved caspase-8/caspase-8 ratio. β-Actin loading control for BAK was obtained from the same gel with CPT1. Protein band intensities are normalized to β-actin and expressed as ratios. Box-and-whisker plots show the median, IQR, and full data range. One-way ANOVA followed by Tukey’s multiple-comparison test; *n* = 4, *P < 0.05, **P < 0.01, ***P < 0.001. Normal human hepatocytes (NHHs) (**D**–**F**). (**D**) Quantitative intracellular caspase-3/7 activity in NHHs and relative fold-changes compared with the control. (**E**) Immunoblot analysis of BAK and cleaved caspase-8/caspase-8 ratio. (**F**) Quantitative cell viability and relative fold-changes compared with the control (100%). Box-and-whisker plots show the median, IQR, and full data range. One-way ANOVA followed by Tukey’s multiple-comparison test; *n* = 3, *P < 0.05 versus non-PA and IL-10 (0 ng/mL) (control); ^#^*P* < 0.05 versus PA and IL-10 (0 ng/mL). BAK, Bcl-2 antagonist or killer; BAX, Bcl-2-associated X protein; HFD, high-fat diet; ND, normal diet.

We next assessed apoptotic markers after IL-10 treatment. In the long-term, cleaved caspase-3 expression in liver tissues was visually and quantitatively evaluated, revealing that IL-10 reduced hepatic cleaved caspase-3 expression induced by the HFD compared with controls (Figure 6A). Consistent with cleaved caspase-3 staining, TUNEL staining revealed that IL-10 suppressed late-stage apoptosis via reduced nuclear DNA fragmentation in HFD-fed mice (Figure 6B). We examined the protein expression of the following apoptotic regulators: Bcl-2 antagonist or killer (BAK), Bcl-2-associated X protein (BAX), and caspase 8 (25, 26). BAK and BAX levels were increased with the HFD compared with controls, but in HFD-fed mice, IL-10 reduced BAK levels and the cleaved caspase-8/caspase-8 (full-length) ratio (Figure 6C). In in vitro NHHs, IL-10 reduced PA-induced caspase-3/7 activity compared with controls (Figure 6D), with similar results observed visually and quantitatively in HepG2 cells (Supplemental Figure 9, A and B). Regarding the effect of IL-10 on hepatocellular apoptosis activation, BAK levels and the cleaved caspase-8/caspase-8 ratio increased after PA exposure compared with controls. However, under PA exposure, IL-10 selectively reduced the cleaved caspase-8/caspase-8 ratio (Figure 6E).

Cell viability showed no significant changes under PA exposure and IL-10 treatment (Figure 6F). This finding was validated in HepG2 cells, where, interestingly, IL-10 increased cell viability reduced by PA exposure, unlike in NHHs (Supplemental Figure 9C).

Furthermore, short-term IL-10 treatment similarly reduced hepatic caspase-3 expression and TUNEL induced by the HFD compared with controls (Supplemental Figure 9, D and E). Regarding apoptosis-activating expression, the results mirrored those of long-term IL-10 treatment (Supplemental Figure 9F). These findings suggest that IL-10 reduced apoptotic markers under both short- and long-term treatment conditions, with sustained reductions observed after prolonged treatment.

### IL-10–associated signaling preserves hepatocellular viability and stress-responsive survival pathways.

**Figure 7:**
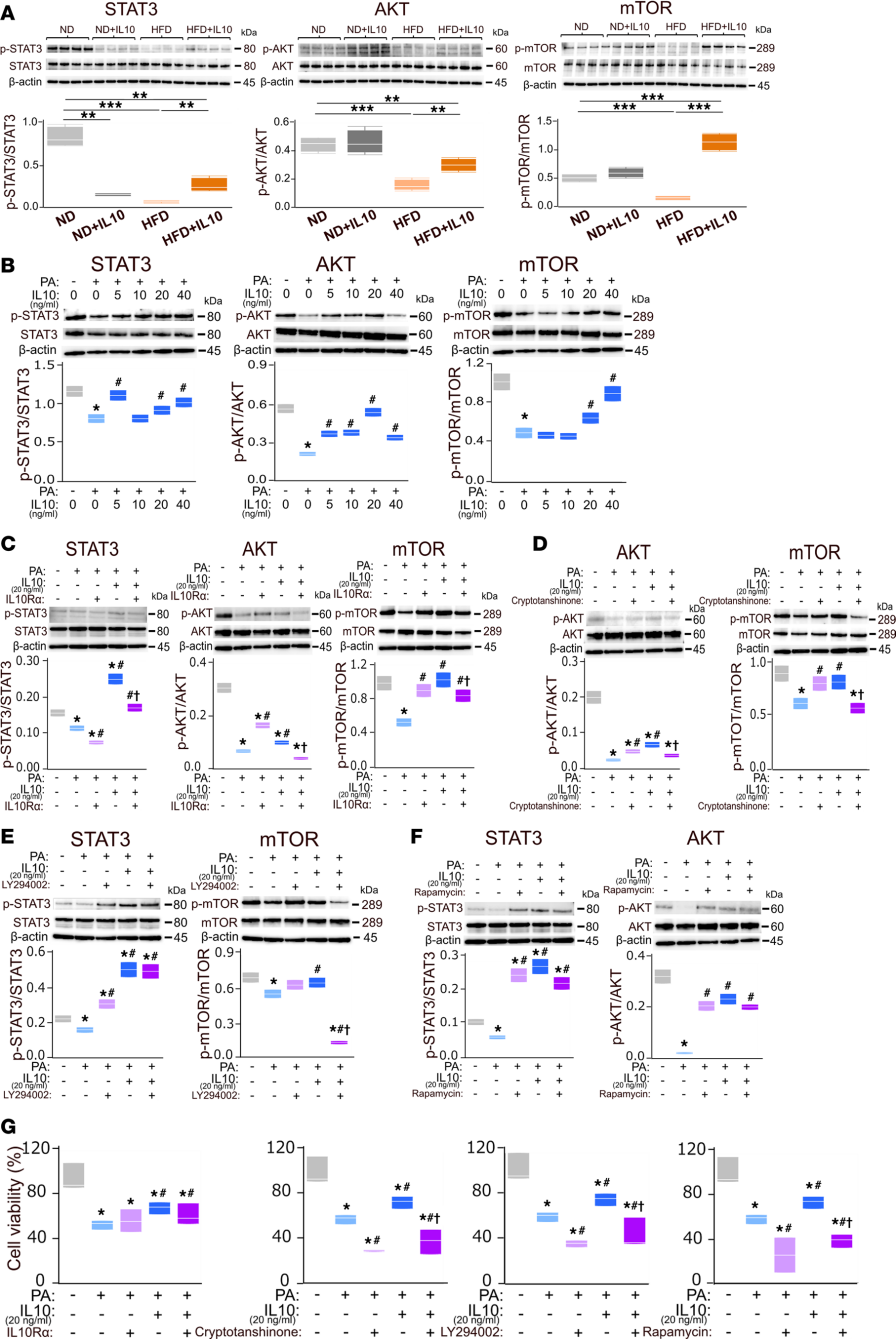
IL-10 preserves hepatocellular viability/metabolic activity and restores stress-responsive STAT3/AKT/mTOR signaling. Phosphorylation ratios are shown under basal conditions in the lipotoxicity model (PA exposure) and interpreted as stress-responsive survival signaling; insulin stimulation was not performed. (**A**) In vivo hepatic STAT3/AKT/mTOR signaling. Immunoblot analysis of p-STAT3/STAT3, p-AKT/AKT, and p-mTOR/mTOR in liver tissues from ND, ND+IL-10, HFD, and HFD+IL-10 groups. Protein band intensities are quantified and expressed as phosphorylation ratios. β-Actin loading controls for AKT and mTOR were obtained from the same gel with STAT3. (**B**) Cells were incubated with PA at increasing concentrations or with DMEM alone (control) for 24 hours. Immunoblot analysis of p-STAT3/STAT3, p-AKT/AKT, and p-mTOR/mTOR. Protein band intensities are normalized to β-actin and expressed as ratios. Box-and-whisker plots show the median, IQR, and full data range. One-way ANOVA followed by Tukey’s multiple-comparison test; *n* = 3, *P < 0.05 versus non-PA and IL-10 (0 ng/mL) (control); ^#^*P* < 0.05 versus PA and IL-10 (0 ng/mL). Cells were then incubated with PA exposure in the presence of IL-10 (0 and 20 ng/mL) and/or (**C**) IL-10Rα, (**D**) cryptotanshinone, (**E**) LY294002, (**F**) rapamycin, or DMEM (control) for 24 hours. (**B**–**E**) For each inhibitor, immunoblot analysis was conducted to evaluate protein expression changes in p-STAT3/STAT3, p-AKT/AKT, and p-mTOR/mTOR ratios, except for the specific pathway targeted by each inhibitor. (**G**) Quantitative cell viability. For each inhibitor, relative fold-changes compared with the control (100%) were examined. Box-and-whisker plots show the median, IQR, and full data range. One-way ANOVA followed by Tukey’s multiple-comparison test; *n* = 3, *P < 0.05 versus non-PA and IL-10 (0 ng/mL) (control); ^#^*P* < 0.05 versus PA and IL-10 (0 ng/mL); ^†^*P* < 0.05 versus PA and IL-10 (20 ng/mL). HFD, high-fat diet; ND, normal diet; PA, palmitic acid.

To investigate signaling associated with IL-10–mediated cytoprotection under lipotoxic stress, we examined protein expression levels of AKT/mTOR/STAT 3 pathway activation in liver tissues of HFD-fed mice and in PA-exposed HepG2 cells treated with IL-10. PA exposure reduced basal p-STAT3/STAT3, p-AKT/AKT, and p-mTOR/mTOR ratios relative to control conditions, whereas IL-10 increased these ratios under PA exposure (Figure 7, A and B), consistent with restoration of stress-responsive survival signaling. Under ND conditions, IL-10 did not increase p-AKT/AKT or p-mTOR/mTOR ratios and was associated with a reduction in basal pSTAT3/STAT3. To support the interpretation of autophagy modulation, we assessed autophagy markers in liver tissue. Consistent with altered mTOR-associated signaling, the HFD increased the LC3-II/I ratio and reduced p62 expression, whereas IL-10 partially reversed these autophagy-related changes, again with no significant differences between the ND and ND+IL-10 groups (Supplemental Figure 10). To probe pathway dependencies, we used specific antagonists: IL-10R, a function-blocking antibody targeting IL-10RA; STAT3, cryptotanshinone; AKT, LY294002; and mTOR, rapamycin. Under PA exposure and IL-10 treatment, IL-10RA reduced p-STAT3/STAT3, p-AKT/AKT, and p-mTOR/mTOR ratios (Figure 7C). Cryptotanshinone reduced p-AKT/AKT and p-mTOR/mTOR ratios (Figure 7D). LY294002 decreased p-mTOR/mTOR but not p-AKT/AKT (Figure 7E). Rapamycin did not alter p-STAT3/STAT3 or p-AKT/AKT ratios (Figure 7F). These inhibitor profiles were consistent with STAT3 activation upstream of AKT and mTOR.

For cell viability, under PA exposure and IL-10 treatment, the relative fold-change decreased with cryptotanshinone, LY294002, and rapamycin but remained unchanged with IL-10RA (Figure 7G). These results suggest that IL-10 activates IL-10R–dependent STAT3/AKT/mTOR signaling under lipotoxic stress and that cell viability–related outcomes depend on STAT3/AKT/mTOR activity, while the impact of IL-10Rα blockade on cell viability was not detectable under the current experimental conditions.

## Discussion

In this study, we evaluated the effects of IL-10 treatment on hepatic responses to lipotoxic stress in MASLD, using HFD-fed mice and PA-exposed cells. We identified IL-10 as a modulator of hepatic homeostatic response pathways under lipotoxic conditions. IL-10 reduced hepatic lipid accumulation, promoted glucose content, and suppressed OS and apoptosis caused by lipotoxicity, consistent with improved homeostatic responses in MASLD. Short-term IL-10 treatment counteracted lipotoxicity and reduced BW, and the long-term treatment maintained BW suppression and enhanced antioxidant activity. Fibrosis scores remained unchanged, although fibrogenic gene expression and activation was reduced. IL-10 systematically exerted similar effects on hepatocytes, with cell context–dependent effects on viability (no significant change in NHHs; partial improvement in HepG2). The lipotoxicity-resistant function of IL-10 is particularly noteworthy beyond its established immunoregulatory functions (13).

IL-10Rα expression was observed in hepatocytes and non-parenchymal regions in HFD-fed mice, and cell type–specific identification of these IL-10Rα–positive cells, including Kupffer cells, hepatic stellate cells, and liver sinusoidal endothelial cells, will be required in future studies. IL-10Rα is primarily localized to the cell membrane in hepatocytes; therefore, intracellular staining observed after IL-10 treatment could be consistent with receptor internalization/trafficking, although this will require dedicated assays to confirm (27). Endogenous IL-10 can arise from hepatic immune compartments, including Kupffer cell subsets implicated in local IL-10 programs in MASLD (28). We did not distinguish endogenous from administered IL-10; however, serum and hepatic IL-10 increased with IL-10 treatment, with no evidence of overall IL-10 signal suppression. IL-10 can act on multiple hepatic cell types; activated hepatic stellate cells can acquire IL-10Rα and IL-10 responsiveness (29), consistent with our staining (Supplemental Figure 5). However, we focused on the therapeutic impact of IL-10 on lipotoxic stress responses and did not map IL-10–producing cell populations. Although direct evidence of IL-10–induced IL-10Rα upregulation in hepatocytes is limited, our findings suggest both hepatocyte-intrinsic and non-parenchymal immunomodulatory effects, warranting future cell type–specific studies targeting Kupffer cells, hepatic stellate cells, and liver sinusoidal endothelial cells. Non-parenchymal cells contribute to immune regulation under lipotoxicity (30), and our findings are consistent with combined hepatocyte IL-10Rα–linked effects and complementary non-parenchymal support.

In both in vitro and in vivo models of MASLD, PA and HFD increase lipid accumulation and alter FA metabolism, while β-oxidation and PPARα expression are unchanged or reduced (31–33). Similar results were observed in this MASLD model, particularly in lipid accumulation and FA synthesis. PA and HFD increased CPT1 and PPARα levels, suggesting that PA/HFD administration may not excessively impair hepatic β-oxidation or PPARα function. IL-10 regulates FA metabolism to limit inflammation, including modulation of lipid handling and β-oxidation programs in macrophages (5, 34). In our models, IL-10 reduced hepatic and hepatocellular lipid accumulation and increased CPT1/2 after prolonged treatment. Long-term IL-10 treatment tended to upregulate PPARα expression, though no significant changes were observed. PPARα is a transcription factor regulating FA synthesis and β-oxidation (35). Interestingly, IL-10 functions as a modulator of FA synthesis and β-oxidation with limited PPARα changes, suggesting PPARα-independent or downstream mechanisms. Further studies are needed to elucidate the detailed mechanisms linking IL-10 and FA metabolism.

In MASLD, BW, serum liver enzymes, T-CHO, and TG levels increase, and liver fibrosis progresses compared with a normal liver (21, 36); these trends were also observed in this MASLD model. Histologically, IL-10 treatment reduced steatosis and lobular inflammation and improved liver fibrosis at the gene expression level in HFD-fed mice. However, visually, liver fibrosis showed minimal changes in HFD-fed mice, indicating a need for further studies using MASLD models with more advanced fibrosis. In other liver diseases with severe fibrosis, IL-10 normalizes liver enzymes and reduces lobular inflammation/hepatitis and fibrosis (37). As hepatocyte ballooning is more prominent in advanced steatohepatitis, the early-stage HFD model with modest ballooning likely limited detection of an IL-10 effect (16, 17). Regarding blood biochemistry, IL-10 reduced serum AST, ALT, TG, T-CHO, and NEFA levels in HFD-fed mice, consistent with improved systemic metabolic stress and hepatic injury. Notably, serum TG showed a context-dependent pattern, with a modest increase in ND+IL-10 but a reduction in HFD+IL-10 compared with HFD. This apparent divergence may reflect differences in whole-body lipid handling between physiological and lipotoxic states. Under HFD, reduced NEFA with improved insulin sensitivity and hepatic lipid reduction suggests improved lipid flux/handling that could lower TG. Under ND, the modest TG increase in the absence of hepatic lipid accumulation may reflect altered lipoprotein production/clearance dynamics rather than pathologic lipotoxicity (38, 39). We did not measure VLDL production, lipoprotein clearance, or adipose lipolysis; these mechanisms require future study.

Regarding glucose metabolism, MASLD is characterized by elevated FBG levels; altered hepatic glucose handling, including changes in gluconeogenic and glycogen-related pathways (4, 40); and increased glycogen synthesis and storage (41). In vitro, PA stimulates glucose uptake and gluconeogenesis (42), and in HFD-fed mice, glucose uptake is promoted and hepatic gluconeogenesis is reduced (40). Similar trends were observed in this MASLD model. The effects of IL-10 on glucose uptake and glycogen synthesis appear context dependent across experimental settings (43), leaving its contribution to hepatic glucose handling incompletely defined. IL-10 reduced fasting blood glucose while increasing hepatic glucose content and enhancing GLUT2/GK and phosphorylation readouts of glycogen enzymes (p-GS Ser641 and p-GP Ser15), and it restored PCK1 levels in HFD-fed mice. Because p-GS (Ser641) is inhibitory and p-GP (Ser15) is activating, total abundance and phospho-states reflect different regulatory layers (44, 45). In HFD-fed mice, short-term IL-10 treatment did not alter hepatic glucose content, and long-term treatment increased it. This reason may be that short-term IL-10 treatment improves insulin sensitivity without sufficient time for hepatic glucose-handling remodeling and net glucose/glycogen accumulation, whereas longer treatment permits cumulative adaptation and measurable hepatic glucose content changes. This pattern suggests that IL-10 reshapes hepatic glucose-handling under lipotoxic stress, with systemic glycemia reflecting integrated hepatic and whole-body effects. The simultaneous increase in inhibitory p-GS and activating p-GP may be most consistent with altered glycogen turnover control in HFD liver rather than a simple unidirectional shift in net glycogen storage. Cellular glucose content does not directly reflect transport, but a 2-NBDG assay showed that PA reduced uptake and IL-10 partially restored it. Discordance with protein abundance may reflect compensatory induction, posttranslational regulation (e.g., trafficking), and intracellular utilization/pooling. Here, we identified IL-10 as a modulator of glucose handling under lipotoxic stress that supports hepatocellular glucose uptake capacity and reshapes glucose-handling pathways, consistent with improved fasting glycemia in HFD-fed mice. IL-10 also protects against diet-induced IR in the liver (46). In this MASLD model, IL-10 suppressed worsening of hyperglycemia and IR and improved insulin sensitivity, which was evaluated as HOMA-IR and QUICKI (23). However, gluconeogenic flux and dynamic insulin sensitivity were not directly measured; therefore, future studies using tracer/pyruvate tests and GTT/ITT (or clamp) will be required (47).

Notably, BW loss induced by IL-10 treatment may contribute to decreased lipid and glucose levels (48); therefore, BW reduction can be a potential confounder. However, several lipotoxicity-related hepatic readouts improved even in the short term where food intake/BW changes were smaller, and hepatocellular experiments under PA exposure support direct IL-10 effects. Future pair-feeding/weight-matched designs and tissue-specific IL-10R approaches will help separate hepatic-intrinsic from systemic contributions. Furthermore, in mice, circulating IL-10 is typically low under physiological conditions, suggesting that our in vitro dose series should be viewed as a mechanistic probe rather than a direct mimic of circulating concentrations (15, 49). Further studies should relate IL-10 dosing to systemic exposure and BW changes in MASLD.

Among ND-fed mice, IL-10 did not affect FA and glucose metabolism, BW, or liver histology, except for glucose uptake. Notably, IL-10 did not reduce BW in HFD-fed mice below the physiological BW gained by ND-fed mice. IL-10 treatment did not cause external changes, abnormal behavior, or mortality in mice. In ND-fed mice, IL-10 had limited effects on most metabolic and histological parameters, suggesting that IL-10’s impact is more apparent under lipotoxic/metabolic stress conditions. Several MASLD therapies are under investigation (50); however, IL-10 augmentation for lipotoxic injury has been less explored. Our findings support further evaluation of IL-10 signaling with careful attention to dosing, safety, and context dependence.

Regarding OS and apoptosis, MASLD is characterized by increased ROS and cell death (36); these trends were also observed in this MASLD model. HFD-fed mice exhibit minimal changes in hepatic antioxidant enzymes (51), and the antioxidant system in MASLD remains controversial. We found that PA and the HFD upregulated the hepatic antioxidant system, likely as a compensatory response to OS exposure. Apoptosis in MASLD is associated with increased cleaved caspase-3 expression (2); this trend was also observed in this MASLD model. However, apoptosis-related proteins BAK/BAX and caspase-8 differed between HFD-fed mice and NHHs. These proteins regulate apoptosis, with BAK/BAX activating caspase-3 via the intrinsic mitochondrial pathway (25, 52), whereas caspase-8 initiates caspase-3 activation through the extrinsic pathway (26, 52). PA and the HFD induced apoptosis via the intrinsic pathway, whereas only PA induced apoptosis via the extrinsic pathway. PA and the HFD trigger intrinsic apoptosis due to increased OS (25, 52). HFD-induced inflammatory responses in hepatocytes also activate extrinsic apoptosis, though short-term HFD may not provide sufficient inflammatory signals to induce this process (53). PA may create hypoxic-ischemic conditions in hepatocytes, leading to extrinsic apoptosis (54). IL-10 has antioxidant properties and suppresses OS (37); however, its precise mechanism remains unclear. Remarkably, IL-10 increased hepatic SOD activity in both ND- and HFD-fed mice through modulation of hepatic antioxidant-related enzymes and suppressed OS under HFD, with long-term IL-10 treatment enhancing antioxidant activity. Among these enzymes, the differential SOD2 versus SOD1 regulation between HFD liver and PA-exposed hepatocytes is consistent with compartmentalized redox control. In hepatocytes, STAT3 signaling preferentially induces mitochondrial SOD2, but not cytosolic SOD1, as IL-10 increased SOD2 in HFD-fed mice (7, 55, 56). In contrast, isolated hepatocytes under acute PA stress lack systemic hormonal cues and multicellular liver interactions and may exhibit predominant cytosolic redox adaptation, reflected by SOD1 induction without a measurable SOD2 change (57). Non-parenchymal cells (including liver sinusoidal endothelial cells) can also shape redox homeostasis (58) and should be addressed in future cell type–resolved studies. HFD increased SOD2 protein without a parallel rise in total SOD activity, possibly reflecting functional limitation under OS (59). CAT expression changes were smaller, suggesting IL-10 preferentially enhances the SOD step. These findings suggest that IL-10 acts as an antioxidant inducer, reducing OS and enhancing antioxidant activity long-term in MASLD. In contrast, IL-10 is known to induce apoptosis (35), though its role in liver disease remains unclear. Among ND-fed mice, some apoptosis-related proteins changed with IL-10; the intrinsic pathway (BAK/BAX) was significantly upregulated. However, in this MASLD model, IL-10 suppressed apoptosis through extrinsic pathway regulation (caspase-8) and via the intrinsic pathway (BAK). Interestingly, although HFD-induced differences between intrinsic and extrinsic apoptosis were observed, IL-10 also functioned as an antiapoptotic inducer, directly reducing apoptosis in MASLD by inhibiting intrinsic and extrinsic pathways. Here, we noted that some in vitro dose-response relationships for IL-10 were non-monotonic, and several protein changes showed quantitative differences between isolated hepatocytes and whole-liver tissue. These nonlinear cytokine responses can occur due to receptor saturation, feedback regulation, cell context–dependent signaling thresholds, species/context differences (mouse liver tissue versus human hepatocytes), multicellular crosstalk and paracrine signaling in vivo (non-parenchymal/immune compartments), or differences in the lipotoxic stimulus (chronic HFD feeding versus acute PA exposure) (60, 61). Therefore, we avoid overinterpreting small differences between individual IL-10 doses and emphasize effects that were consistent across experiments.

IL-10 maintains cell viability via the STAT3/AKT/mTOR pathways (15). IL-10 promoted cell survival impaired by PA exposure through a similar mechanism. IL-10 activated STAT3/AKT/mTOR via IL-10R; however, IL-10Rα blockade reduced the phosphorylation, whereas its effect on cell viability was not detectable in our assay conditions. This discrepancy may reflect incomplete receptor blockade and/or parallel STAT3-activating inputs, which can be prominent in transformed hepatocyte lines; our findings may be supportive but not definitive evidence of exclusive IL-10R dependence. This STAT3/AKT/mTOR axis may also apply to MASLD (15). PA and the HFD reduced basal p-STAT3/p-AKT/p-mTOR ratios, and IL-10 restored lipotoxic stress-responsive survival signaling toward control levels, consistent across liver tissue and HepG2 findings. Under ND conditions, IL-10 did not increase basal p-AKT/p-mTOR and reduced basal p-STAT3, suggesting that IL-10 does not simply elevate these signals in the absence of metabolic stress. Consistent with the limited proliferative capacity of primary hepatocytes in culture, we therefore describe these findings as preservation of hepatocellular viability/metabolic activity rather than induction of proliferation. Given that HepG2 cells are derived from hepatocellular carcinoma and do not fully recapitulate primary hepatocyte biology, HepG2 cells were adopted primarily as a complementary system to probe signaling relationships, whereas our core hepatocellular phenotypes were established in NHHs and validated in vivo. We further assessed autophagy-related markers in vivo and found that the HFD increased LC3-II/I and reduced p62, whereas IL-10 reversed these changes. Although these markers do not directly quantify autophagic flux, they support the involvement of mTOR/autophagy-associated pathways in IL-10–mediated protection under lipotoxic stress and provide a plausible framework for the observed reductions in OS and apoptosis (15, 62). Here, we identified IL-10 as a cytoprotective/cell viability–related inducer under lipotoxic stress in MASLD. These signaling changes link IL-10R engagement to downstream modulation of OS and apoptotic pathways under lipotoxic conditions, although cell type–specific necessity and upstream regulators will require further study.

This study provides evidence that IL-10 modulates hepatic lipotoxicity through gene and protein expression analyses; however, we acknowledge several limitations. First, our HFD-fed C57BL/6J model represents early-stage MASLD with limited histological fibrosis, limiting inference for advanced MASH/fibrosis; more fibrogenic models (e.g., Gubra amylin NASH–type diets) will be needed in future studies (63). Second, only male mice were examined; sex-dependent IL-10 responses will require future study. Third, IL-10 gene expression is elevated in human peripheral immune cells and correlates with hepatocyte ballooning in patients with MASLD (16). However, hepatic and circulating IL-10 measurements can vary across cohorts (19), and IL-10/IL-10Rα signaling in human liver tissue was not assessed. Hepatic IL-10Rα expression observed in our MASLD model mice may reflect a lipotoxicity-related pathological response relevant to disease progression. In addition, we did not directly quantify gluconeogenic flux (e.g., tracer-based measurements or pyruvate tolerance testing); therefore, changes in PCK1 and related proteins should be viewed as pathway-associated readouts rather than definitive evidence of increased gluconeogenesis. Further translational validations will be required, including analysis of human liver tissue and evaluation of IL-10–based strategies for MASLD. Fourth, although IL-10Rα blockade and pathway inhibitors support receptor-dependent signaling in vitro, cell type necessity will require conditional loss-of-function models (e.g., hepatocyte- versus macrophage- versus stellate cell–specific IL-10R deletion) and/or IL-10 deletion. These approaches are needed to separate hepatocyte-intrinsic effects from indirect paracrine mechanisms via non-parenchymal cells. Finally, although we demonstrate the multifaceted regulatory effects of IL-10, detailed mechanistic insights (e.g., upstream signaling pathways, transcriptional regulation, and cell type–specific responses) were beyond the scope of this study. Nevertheless, our findings motivate mechanistic studies to define cell type–specific pathways for MASLD therapy.

In conclusion, IL-10 attenuates lipotoxic injury by modulating hepatic homeostatic response pathways, thereby improving MASLD-related phenotypes, supporting further evaluation of IL-10 signaling as a therapeutic strategy. Increased IL-10 in MASLD may serve as a physiological compensatory response to lipotoxicity, in addition to suppression of hepatitis and autoimmune factors (13, 16, 17). However, endogenous IL-10 alone may be insufficient in vivo, suggesting a role for therapeutic supplementation. Our findings suggest that the homeostatic response of IL-10 in lipotoxicity presents a promising treatment option for MASLD and may extend to other lipotoxicity-related contexts, such as age-related diseases (metabolic disorders, malignancy, and cardiovascular disease) (8, 9).

## Methods

### Sex as a biological variable.

Only male C57BL/6J mice were used in this study. Male mice were selected because this diet-induced MASLD model has been extensively characterized in males and was used to reduce biological variability during this revision-focused mechanistic study. The relevance of the findings to females remains to be determined and should be addressed in future studies.

### Cell culture.

NHHs (SCR, 5200) and HepG2 cells (European Collection of Authenticated Cell Cultures, EC85011430-G0) were purchased, with the former used as an NHH model and the latter as an immortalized model with distinct properties. Both cell types were suspended in DMEM (Gibco, 11965092) supplemented with 10% FBS (Biosera, 556-33865), 100 U/mL penicillin, and streptomycin (Merck, P7539). The cells were adjusted to 5.0 × 10^5^/mL and cultured at 37°C in a humidified incubator with 5% CO_2_ for 24 hours in 6-well plates (1.0 × 10^6^ cells/well) in DMEM. Next, cells were cultured in 1 well with DMEM (control) and in the remaining 5 wells with PA under varying concentrations of recombinant human IL-10 (PeproTech, 200-10) (0, 5, 10, 20, and 40 ng/mL) for 24 hours as previously described (15). PA (Merck, p5585), widely used for in vitro MASLD assessment (2, 64), was prepared at 150 μM PA/BSA (Wako) in DMEM. Vehicle controls for PA experiments consisted of BSA-only treatment to match PA-BSA conditions. After treatment, cells were collected, washed 2–3 times with PBS (Gibco, 10010023) at 1,500*g* for 5 minutes at 23°C, and analyzed biochemically at 70%–80% confluence.

To assess IL-10–mediated cell viability via the STAT3/AKT/mTOR pathways, phosphorylation of STAT3, AKT, and mTOR was examined in HepG2 cells after exposure to PA and IL-10 (20 ng/mL) with specific inhibitors: IL-10Rα (1:500 dilution; Santa Cruz Biotechnology, 365374), LY294002 (50 μM; Beyotime, S1737), cryptotanshinone (4.6 μM; Selleckchem, S2285), or rapamycin (1 μg/μL; Enzo, BML-A275) for 24 hours, as previously described (15). IL-10Rα selectively inhibited IL-10R, cryptotanshinone targeted p-STAT3, LY294002 targeted p-AKT, and rapamycin p-mTOR.

### Mouse rearing.

WT (C57BL/6J) male mice (4 weeks old) were purchased from The Jackson Laboratory and fed either an ND or an HFD (CLEA Japan, 32% animal fat) for 12 weeks after a 2-week adaptive feeding period (*n* = 8 per group), a method known to induce MASLD (65). Mice were then treated with or without recombinant mouse IL-10 (50 μg/kg; PeproTech, 210-10) every 2 days for 6 weeks before euthanasia (*n* = 4 per group), as previously reported (66). BW was measured weekly after adaptive feeding, and LW and LW/BW ratios were recorded at euthanasia. Food intake was assessed during the IL-10 treatment period by placing a known amount of chow in each cage and weighing the remaining chow at 24-hour/48-hour/7-day intervals. Spillage was collected from cage bedding (when feasible) and subtracted from the estimated intake. Food intake was expressed as g/d per mouse by dividing cage-level intake by the number of mice in the cage. Whole-blood samples collected at euthanasia were centrifuged at 1,000*g* for 10 minutes, and serum was separated and stored at –20°C for biochemical analysis (Oriental Yeast Co.). No mortality occurred in any experimental group during the study period. Biochemical parameters included AST, ALT, TG, T-CHO, NEFA, FBG, and insulin. The HOMA-IR score was calculated to assess glucose tolerance and insulin resistance (67). Serum IL-10 concentrations were measured at euthanasia using Mouse IL-10 Quantikine ELISA kit (R&D Systems) according to the manufacturer’s protocol. To quantify hepatic IL-10, liver tissues (50 mg) were homogenized in PBS with protease inhibitor, clarified by centrifugation, and IL-10 levels in the supernatant were measured by ELISA and relatively calculated compared with the control. Samples were collected at euthanasia after an overnight fast, 24 hours after the last IL-10 injection.

### Histological and lipid assessment.

At euthanasia, liver tissue samples were fixed in 10% buffered formalin, embedded in paraffin, and sectioned at 5 μm for histology, Oil Red O staining, and IHC. The histological assessment included H&E for diagnosis and Masson’s trichrome staining for fibrosis. Steatosis, lobular inflammation, ballooning, and fibrosis scores were quantified as previously reported (16, 18). Liver disease in the samples was assessed using an Oil Red O stain kit (ScyTek) following the manufacturer’s instructions. Cytocentrifugation of 1 or 2 drops of the removed fluid concentrated the samples onto glass slides, which were then stained using a modified Disbrey and Rach procedure with stock Oil Red O (0.1% in isopropanol) diluted to 60% with distilled water. Images were acquired and digitized using a Leica DM6000B microscope with an attached digital camera. Liver lipid concentrations were quantified using ImageJ (NIH) by selecting red-stained lipids and calculating the percentage per area. One image per mouse (*n* = 4 per group) was analyzed to obtain mean values. Additionally, 5 μm–thick FFPE sections were deparaffinized, dehydrated, autoclaved in 10 mM citrate buffer (pH 6.0), incubated with normal swine serum, and stored frozen for IHC evaluations.

### Immunostaining.

Hepatic IL-10Rα immunostaining was assessed using frozen liver cross sections. The tissue sections were dewaxed and treated with 3% hydrogen peroxide for 15 minutes, followed by blocking with goat serum for 30 minutes, incubation at 4°C overnight with a primary mAb against IL-10Rα (1:100 dilution; Santa Cruz Biotechnology, 365374), and immunostained with DAB Substrate kit (Thermo Fisher Scientific) according to the manufacturer’s instructions. To assess fibrogenic cell activation and ability to respond to IL-10, we performed double immunostaining for IL-10Rα (DAB Substrate kit) and α-SMA (Alkaline Phosphatase Substrate kit; Vector Laboratories) in frozen liver sections using a similar method. α-SMA–positive areas were quantified using ImageJ by threshold-based area fraction measurements from multiple fields per mouse. Briefly, tissue sections were incubated with a biotinylated secondary antibody, treated with streptavidin-biotin-HRP for signal amplification, and then stained with diaminobenzidine. Finally, the tissue sections were counterstained with hematoxylin. Isotype-matched IgG was used as a negative control for each immunostaining procedure.

### Cellular lipid assessment.

Cellular lipid droplet accumulation was assessed visually and quantitatively using LipiDye II (Funakoshi) with fluorescence microscopy imaging according to the manufacturer’s instructions, as previously reported (68). As described in *Cell culture*, NHHs and HepG2 cells were cultured in 96-well black plates (5.0 × 10^4^ cells/well) in triplicate and gelatin-coated 6-well plates (2.0 × 10^6^ cells/well), respectively, followed by incubation with 1 μM LipiDye II reagent at 37°C in DMEM for 30 minutes and 2–3 PBS washing steps before and after staining. The fluorescence intensity of cellular lipid droplets in NHHs was measured at 485/535 nm using a DTX plate reader (Beckman Coulter) from 3 independent experiments. Visual imaging was not possible due to unstable cell fixation. For nuclear staining, Hoechst 33342 (Invitrogen) or DAPI was used. Cellular lipid droplets in HepG2 cells were visualized using a fluorescent microscope (EVOS FL Imaging System, Invitrogen) via the GFP channel (green, lipid droplet) and DAPI channel (blue), with images from more than 10 cells per experiment (3 independent experiments). Fluorescence intensity was measured using the same method, and relative fold-changes compared with the control were examined.

### Glucose assessment.

Cellular and hepatic glucose storage were quantified using the Glucose Assay kit-WST (Dojindo, 346-0411) with absorption spectroscopy, following the manufacturer’s instructions, as previously reported (69). As described in *Cell culture*, both cell types were cultured in 96-well plates (5.0 × 10^4^ cells/well) in triplicate, followed by incubation with a prescribed concentration of WST at 37°C in DMEM for 30 minutes. Crushed mouse liver tissue was processed similarly. Glucose consumption was normalized to average cell numbers and sample weights. Absorbance at 450 nm was measured using a DTX plate reader, and relative fold-changes to the control were calculated.

Glucose uptake was assessed in NHHs using the fluorescent glucose analog 2-NBDG (Cayman Chemical), following the manufacturer’s instructions, as previously reported (70). After treatment with palmitate with or without IL-10, cells were incubated with 2-NBDG for 2 hours, then washed with PBS, and intracellular fluorescence was quantified using a plate reader (excitation/emission: 475/550 nm). Fluorescence intensity was measured using the same method, and relative fold-changes compared with the control were examined.

### ROS and antioxidant detection.

Cellular ROS formation was assessed using a DCFDA/H2DCFDA Cellular ROS Assay kit (Abcam, ab113851) according to the manufacturer’s instructions (7). NHHs and HepG2 cells were cultured, then incubated with 20 μM DCFDA at 37°C in the dark for 45 minutes, washed with PBS, and covered in a mounting medium containing DAPI. Fluorescence intensity was measured at 485/535 nm using a DTX plate reader from 3 independent experiments. HepG2 ROS were visualized using a fluorescent microscope (EVOS FL Imaging System, Invitrogen) via the GFP channel (green, ROS) and DAPI channel (blue). Images were obtained from more than 10 cells per experiment (3 independent experiments). Fluorescence intensity was measured using the same method, and relative fold-changes compared with the control were examined.

Hepatic ROS formation was assessed using frozen liver cross sections. Cross sections were washed with ice-cold PBS for 5 minutes, incubated with 300 nM DCFDA in PBS at 37°C for 30 minutes, followed by a DAPI-containing mounting medium. To specifically evaluate nuclear superoxide production, adjacent sections were incubated with 10 μM DHE (1:500 dilution; Thermo Fisher Scientific, D11347) in PBS at 37°C for 30 minutes in the dark. Slides were mounted using anti-fade mounting media and visualized with fluorescent microscopy. The ROS-positive area (percentage) was quantified using ImageJ (NIH) software by selecting green-stained regions (DCFDA) and subtracting blue-stained (DAPI) areas. For DHE staining, red fluorescence intensity within nuclear regions was quantified to assess superoxide accumulation. The proportional area of ROS-stained tissue was digitally quantified and expressed per area. One image per mouse (4 per group) was analyzed to obtain mean values.

For hepatic antioxidant enzyme activities, total SOD and CAT activity were measured in liver homogenates using a Superoxide Dismutase Assay kit (Cayman Chemical, 706002) and Catalase Assay kit (Cayman Chemical, 707002) and normalized to tissue/protein amount, as previously described (71).

### Cell viability.

Cell viability was assessed using an XTT Cell Viability Assay kit (Biological Industries, 20-300-1000) following the manufacturer’s instructions (7). As outlined in *Cell culture*, both cell types were seeded in 96-well plates (5.0 × 10^4^ cells/well) in triplicate. After incubation, the culture medium was removed, and 100 μL of fresh DMEM was added to each well. Subsequently, 50 μL of activated XTT solution was introduced, and samples were incubated for 2 hours at 37°C. Absorbance was measured at 450 nm using the previously described DTX plate reader. Cell viability was calculated as follows: Cell viability % = (Absorbance of sample – Blank) / (Absorbance of control – Blank) × 100.

### Apoptosis detection.

Cellular apoptotic activity was assessed using CellEvent Caspase-3/7 Green Detection reagent (Invitrogen, C10423). Both cell types were cultured and incubated with 5 μM of the reagent and DAPI for 30 minutes at room temperature. Fluorescence intensity corresponding to apoptosis in NHHs was measured at 502/530 nm using the previously described DTX plate reader from 3 independent experiments. HepG2 apoptosis was visualized using FITC and Alexa Fluor 488 dye; apoptotic cells exhibited green fluorescence. Representative images were captured from at least 10 cells per field across 3 independent experiments. Fluorescence intensity measurements in HepG2 cells followed the same protocol. In both cell types, relative fold-changes in apoptosis compared with the control were determined.

Hepatic apoptosis was examined in frozen liver cross sections using a cleaved caspase-3 antibody (1:400 dilution; Cell Signaling Technology, 9661) (72). Cross sections were deparaffinized in xylene for 10 minutes and rehydrated using ethanol and distilled water. Antigen retrieval was performed by placing samples in 0.01 M citrate buffer and heating them in a microwave for 10 minutes. Samples were blocked with 5% BSA in TBS for 20 minutes before overnight incubation with the primary antibody at 4°C. After 3 washes with TBS, samples were incubated with the secondary antibody for 30 minutes at room temperature. After 3 additional washes in TBS, samples were mounted with a DAPI medium. To further evaluate apoptotic activity, nuclear DNA fragmentation was assessed using the Cell Meter Live Cell TUNEL Apoptosis Assay kit (Green Fluorescence; AAT Bioquest). Frozen liver sections were incubated with the TUNEL reaction mixture according to the manufacturer’s instructions. This method specifically detects late-stage apoptosis by labeling fragmented DNA within the nucleus, resulting in distinct green fluorescence confined to nuclear regions (73). In contrast, cleaved caspase-3 staining reflects mid-stage apoptosis and typically shows broader cytoplasmic distribution, occasionally extending into surrounding tissue structures (74). Apoptotic activity was assessed visually and quantitatively as described in *ROS and antioxidant detection*.

### Quantitative real-time PCR.

mRNA was extracted from liver tissues using the RNeasy Micro kit (QIAGEN), and complementary DNA was synthesized using the RT2 First Strand kit (QIAGEN), as previously described (16). mRNA expression of liver fibrosis–related genes (COL1A1 and COL1A2) was quantified using TaqMan PCR with the corresponding TaqMan probes (Thermo Fisher Scientific). The specific PCR primers and probes used were as follows: COL1A1, Hs00164004_m1; COL1A2, Hs01028956_m1. The relative expression of target gene transcripts was determined as the mean abundance from 3 independent reactions. Expression levels were normalized to GAPDH, and relative expression values were calculated accordingly.

### Western blotting.

Western blotting was performed to assess in vitro and in vivo protein expression changes related to FA and glucose metabolism, OS, apoptosis, and cell viability, as previously described (2, 15, 64). Cells or tissues were lysed and homogenized in RIPA lysis and extraction buffer (Thermo Fisher Scientific, 89901) supplemented with 0.1% protease inhibitor cocktail (Merck, P8340). For phosphorylation analyses of GS, GP, STAT3, AKT, and mTOR, the protease inhibitor cocktail was replaced with a protease and phosphatase inhibitor cocktail (Thermo Fisher Scientific, 78442) at a concentration of 10 μL/mL. Approximately 30 μg of protein was separated by SDS-PAGE (Bio-Rad Laboratories) and transferred onto a PVDF membrane (Millipore). After blocking with 5% BSA in Tris-buffered saline containing 0.1% Tween 20 (TBST), the membrane was rinsed and incubated overnight at 4°C with specific primary antibodies (detailed in Supplemental Table 1). After TBST washes, membranes were incubated with HRP-conjugated secondary antibodies, including Rabbit anti-Mouse IgG (H+L) Secondary Antibody, HRP (Invitrogen, 31450), and Goat anti-Rabbit IgG (H+L) Secondary Antibody, HRP (Invitrogen, 31460), at a 1:2,000 dilution for 1 hour at room temperature. Chemiluminescent signals were developed at multiple exposure times to avoid saturation and to maintain signals within a linear range using an ECL substrate, and membrane signals were detected using the ImageQuant LAS 4000 mini system (GE HealthCare). For some targets with closely spaced molecular weights, membranes were cut and probed separately. For selected targets analyzed from the same membrane, the membrane was stripped after initial detection and re-probed with additional primary antibodies or β-actin using WB stripping solution (ATTO, WSE7240) according to the manufacturer’s instructions. In such cases, the same membrane and lane set were used for sequential probing, and a single β-actin loading control is shown once for the corresponding group of proteins. For experiments in which proteins were analyzed on separate gels/blots processed in parallel using the same lysates, each gel/blot was assigned its own matched loading control, and panels derived from different gels/blots are separated by white space in the figures. Band intensities were quantified using ImageJ. Expression of β-actin was used as the internal loading control. All experiments were performed in triplicate. Notably, in NHHs, BAX protein expression levels were extremely low and could not be assessed.

### Statistics.

Data were analyzed using JMP 17 software (SAS Institute Japan). A *P* value less than 0.05 was considered significant. Quantitative data are presented as box-and-whisker plots showing the median, IQR, and full data range. Comparisons between 2 groups were performed using a 2-tailed unpaired Student’s *t* test. For experiments involving more than 2 groups, 1-way ANOVA followed by an appropriate post hoc multiple-comparison test was used.

### Study approval.

All animal experiments were approved by the IACUC of The University of Tokyo, Tokyo, Japan (P15-078). No human participants were enrolled, and no identifiable human data were collected in this study.

### Data availability.

Values for all data points in graphs are reported in the Supporting Data Values file. Uncropped immunoblot images are provided in the blot file. No custom analytic code was used in this study. Additional data supporting the findings of this study are available from the corresponding author upon reasonable request.

## Author contributions

AK contributed to the study design; data acquisition, analysis, and interpretation; and manuscript drafting. KO contributed to the study design and data acquisition and analysis. TT contributed to study design, data interpretation, and manuscript drafting and managed manuscript submission and correspondence with the Journal. TK, KI, HY, and KM critically revised the manuscript. KK contributed to data analysis, data interpretation, manuscript drafting, and critical revision. MF contributed to data analysis and interpretation, manuscript drafting, critical revision, and supervision. All authors reviewed and approved the final version of the manuscript. KK and MF jointly supervised this study.

## Conflict of interest

The authors have declared that no conflict of interest exists.

## Funding support

- JSPS KAKENHI grant JP24K18936 to AK.
- Health Sciences Research Grant from the Ministry of Health, Labour and Welfare of Japan (Research on Hepatitis) to KK.
- Japan Agency for Medical Research and Development grant JP23fk0210090 to KK.

## Supplementary Material

Supplemental data

Unedited blot and gel images

Supporting data values

## Version 1 04/23/2026

In-Press Preview

## Version 2 06/22/2026

Electronic publication
