# Supplementary material for: IL-10 attenuates metabolic dysfunction–associated steatotic liver disease via modulation of hepatic responses to lipotoxicity
Source: JCI Insight. 2026 Apr 23;11(12):e200231. doi: 10.1172/jci.insight.200231 (PMC13313487; doi:10.1172/jci.insight.200231)
Supplement: Supplemental data [file jciinsight-11-200231-s070.pdf]

Supplemental Material

Interleukin-10 attenuates metabolic dysfunction-associated steatotic liver disease via modulation of hepatic responses to lipotoxicity

Akira Kado, Kazuya Okushin, Takeya Tsutsumi, Toshiyuki Kishida, Kazuhiko Ikeuchi, Hiroshi Yotsuyanagi, Kyoji Moriya, Kazuhiko Koike, and Mitsuhiro Fujishiro

Table of contents

Supplementary table (Table S1) 2
Supplementary figures (Fig. S1-6) 3

Supplementary table

Table S1. Specific primary antibodies for western blot analysis

Name	Host	Dillution	Supplier	Cat no.
IL10Rα	Mouse monoclonal	1:200	Santa Cruz, USA	sc-365374
FAS	Rabbit monoclonal	1:1000	CST	3180
CPT1	Rabbit monoclonal	1:1000	CST	12252
CPT2	Rabbit monoclonal	1:2000	abcam, UK	ab181114
PPARα	Rabbit monoclonal	1:1000	abcam, UK	ab126285
GLUT2	Rabbit monoclonal	1:200	Santa Cruz, USA	sc-9117
GK	Mouse monoclonal	1:200	Santa Cruz, USA	sc-17819
GS	Rabbit monoclonal	1:1000	CST	3893
Phospho-Glycogen Synthase (Ser641)	Rabbit monoclonal	1:1000	CST	3891
GP	Rabbit monoclonal	1:1000	proteintech, USA	55429-1-AP
Phospho-PYGL (Ser15)	Rabbit monoclonal	1:1000	Invitrogen	PA5-114628
PCK1	Rabbit monoclonal	1:1000	CST	12940
SOD1	Rabbit monoclonal	1:1000	proteintech, USA	10269-1-AP
SOD2	Rabbit monoclonal	1:1000	proteintech, USA	24127-1-AP
CAT	Rabbit monoclonal	1:1000	proteintech, USA	21260-1-AP
GPX1	Rabbit monoclonal	1:1000	proteintech, USA	29329-1-AP
BAK	Rabbit monoclonal	1:1000	CST	12105
BAX	Mouse monoclonal	1:2000	BD Biosciences, USA	556467
Caspase 8	Mouse monoclonal	1:2000	CST	9746
STAT3	Rabbit monoclonal	1:1000	CST	12640
p-STAT3	Rabbit monoclonal	1:500	CST	9145
AKT	Rabbit monoclonal	1:1000	CST	9272
p-AKT	Rabbit monoclonal	1:500	CST	4060
mTOR	Rabbit monoclonal	1:1000	CST	2972
p-mTOR	Rabbit monoclonal	1:500	CST	2971
LC3A/B (D3U4C)	Rabbit monoclonal	1:1000	CST	13118
SQSTM1/p62	Rabbit monoclonal	1:1000	CST	8025
β-actin	Rabbit monoclonal	1:1000	CST	44970

Supplementary figures

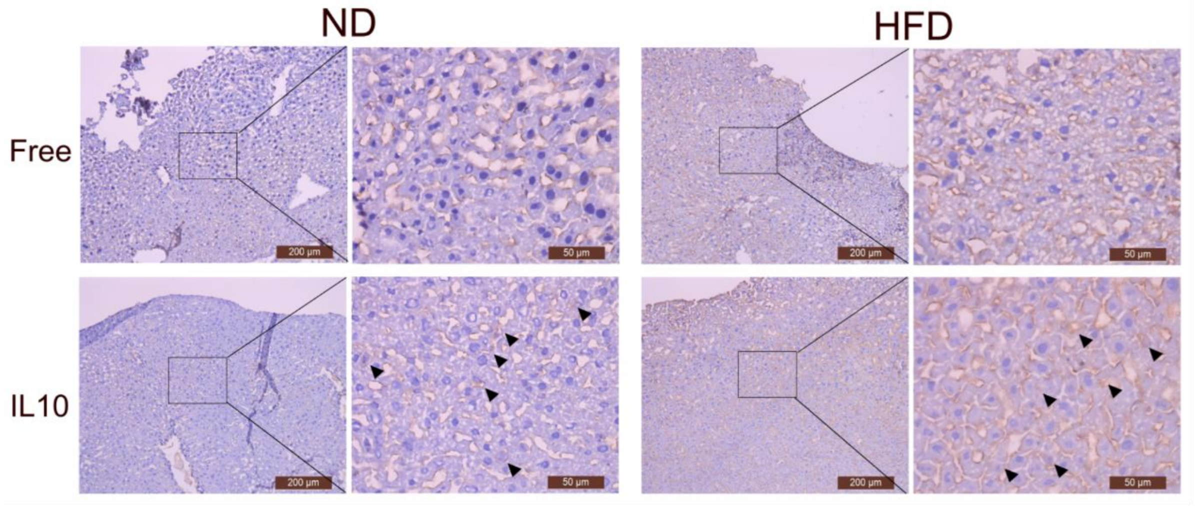

Fig. S1. Immunohistochemical staining of IL-10Rα in liver tissue sections of ND- and HFD-fed mice. Upper panel, no treatment; Lower panel, IL-10 treatment. Stronger staining is observed in the intracellular area of hepatocytes (arrowheads). The staining observed in perivascular regions of HFD-fed mice may reflect increased IL-10Rα expression in non-parenchymal cells such as Kupffer cells and perivascular immune cells under hepatic inflammation. Scale bars = 200, 50 µm. HFD, high-fat diet; ND, normal diet.

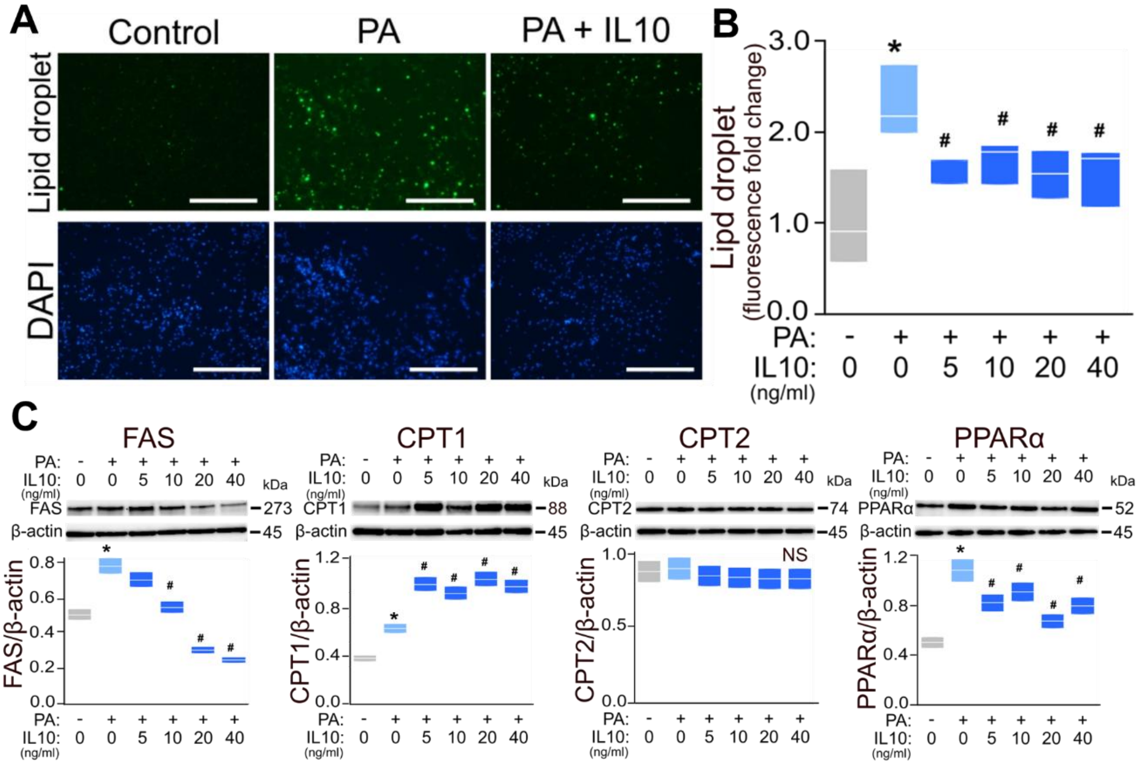

Fig. S2. IL-10 regulates hepatocellular lipid accumulation in HepG2 cells (A-C). HepG2 cells are incubated with DMEM and PA at increasing concentrations of 0, 5, 10, 20, and 40 ng/mL or with DMEM alone (control) for 24 h. (A) FMI of GFP channel (green, LD) and DAPI channel (blue) in HepG2 cells. Immunocytochemistry is performed using an anti-GFP antibody for LD detection, and subcellular localization following UV irradiation is captured using FMI. The upper panel displays raw images acquired in the GFP channel, while the lower panel shows the corresponding DAPI channel. Scale bar = 1000 μm. (B) Cellular LD accumulation is quantified using FMI, and relative fold changes compared to the control are examined. (C) Immunoblot analysis of FAS, CPT1, CPT2, and PPARα. Protein band intensities are normalized to β-actin and expressed as ratios. Data are presented as box-and-whisker plots showing the median, interquartile range, and full data range. One-way ANOVA followed by Tukey's multiple-comparisons test; n = 3, **p*<0.05 versus non-PA and IL-10 0 ng/mL (control); #*p*<.05 versus PA and IL-10 0 ng/mL. LD, lipid droplet; PA, palmitic acid.

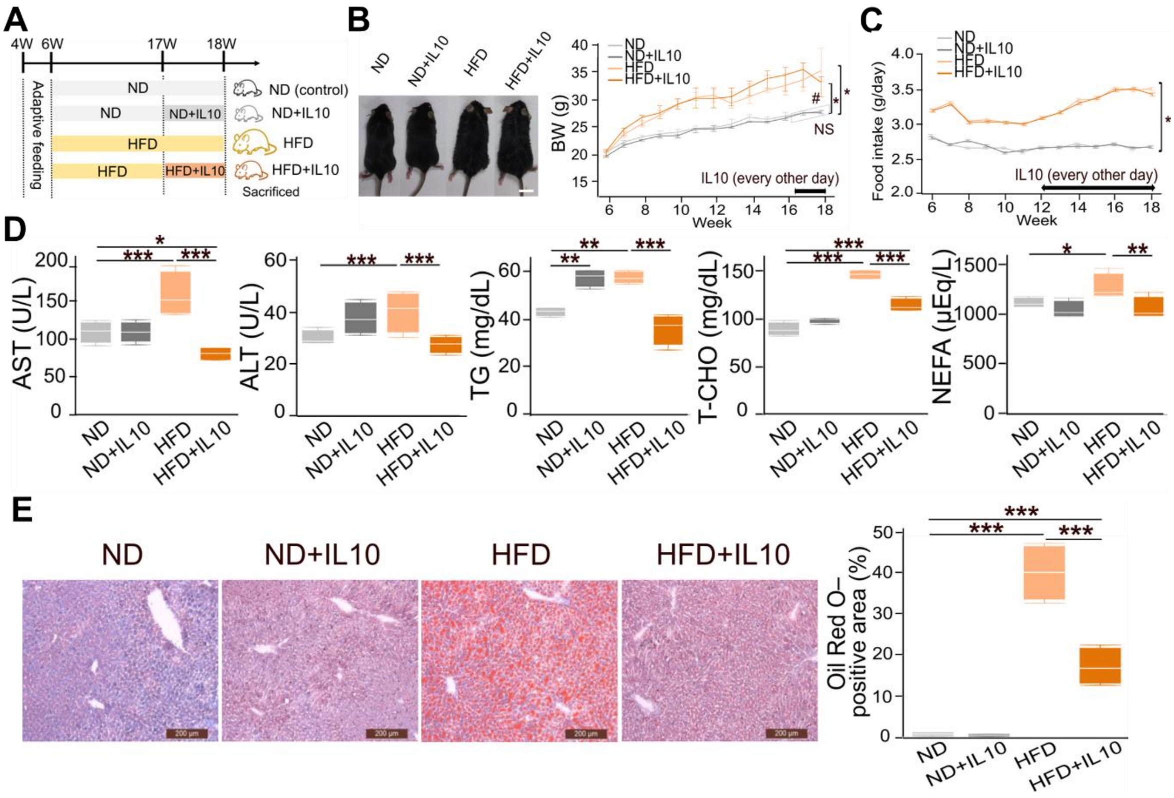

Fig. S3. Short-term IL-10 treatment suppresses hepatic lipid accumulation in HFD-fed mice. (A) Following adaptive feeding, mice are fed either an ND or HFD for 12 weeks and subsequently treated with or without IL-10 once every 2 days for 1 week before sacrifice. (B) Representative images of mice from four experimental groups (ND, ND + IL-10, HFD, and HFD + IL-10) are captured before sacrifice, and BWs in each group are monitored and recorded. Data are presented as box-and-whisker plots showing the median, interquartile range, and full data range. One-way ANOVA followed by Tukey's multiple-comparisons test; n = 4, *p<0.05 versus ND-fed mice (control); #p<0.05 versus HFD-fed mice. Scale bar = 2 cm. (C) Food intake in each group. Food intake is monitored throughout the treatment period and expressed as mean [g/day] per mouse (D) Fasting serum levels of AST, ALT, TG, T-CHO, and NEFA are measured at sacrifice. (E) Oil Red O staining is performed on liver tissue sections, and the Oil Red O-positive area (%) in each view is quantified. Scale bar = 200 µm. The analysis is performed four times per group using different views. Blood biochemistry and immunoblot data are presented as box-and-whisker plots showing the median, interquartile range, and full data range. One-way ANOVA followed by Tukey's multiple-comparisons test; n = 4, *p<0.05, **p<0.01, ***p<0.001. BW, body weight; CPT, carnitine palmitoyltransferase; FAS, fatty acid synthase; HFD, high-fat diet; LD, lipid droplet; ND, normal diet; PPAR, peroxisome proliferator-activated receptor.

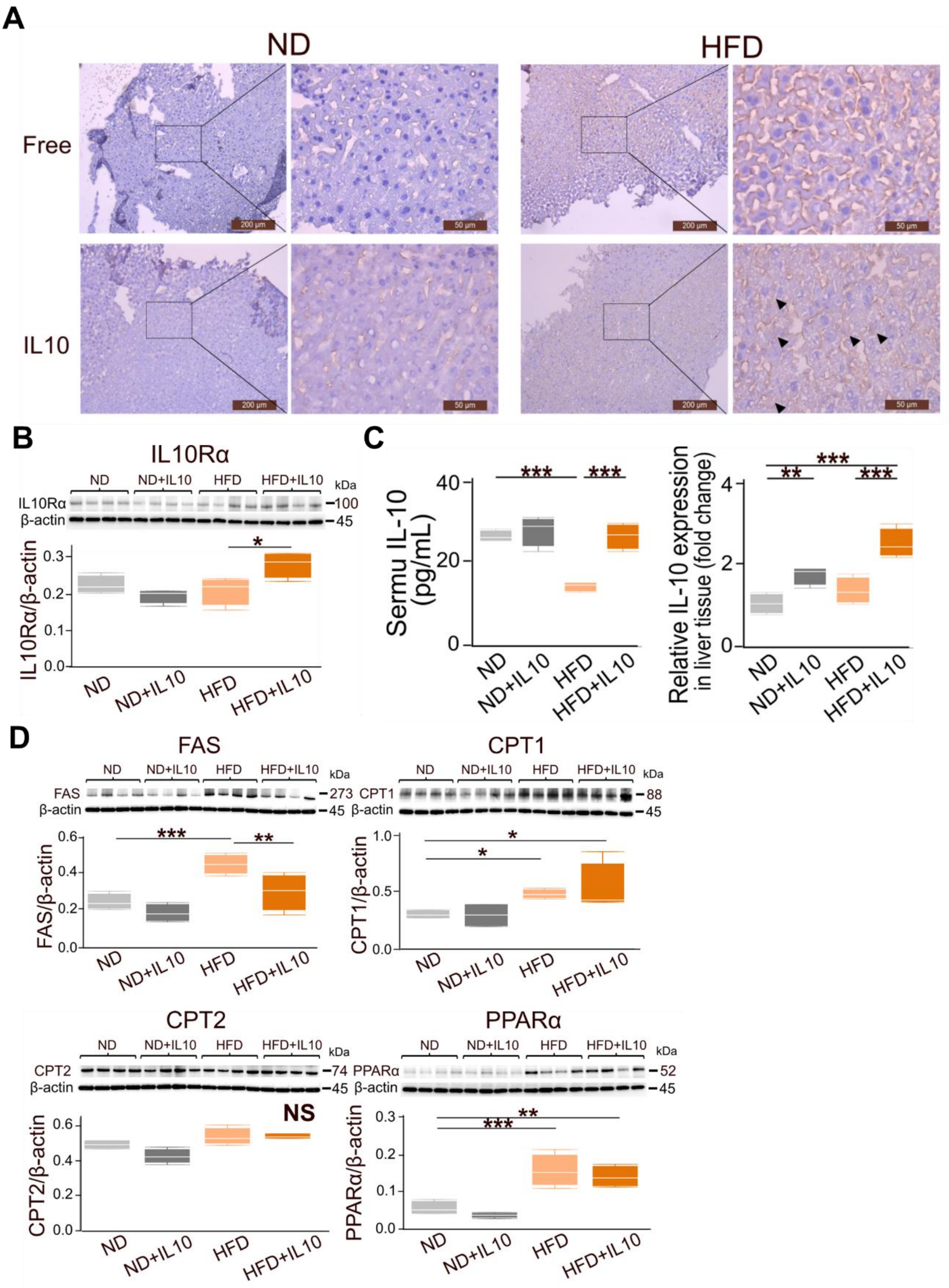

Fig. S4. Short-term IL-10 treatment promotes IL-10 and IL-10Rα expression and suppresses hepatic fatty acid synthase in HFD-fed mice. (A) Immunohistochemical staining of IL-10Rα in liver tissue sections of ND- and HFD-fed mice. Upper panel, no treatment; lower panel, IL-10 treatment. Stronger staining is observed in the intracellular area of hepatocytes (arrowheads). Scale bars = 200, 50 μm. (B) Immunoblot analysis of IL10Rα.

Protein band intensities are normalized to β-actin and expressed as ratios. (C) Serum IL-10 levels at sacrifice and quantitative hepatic IL-10 levels and relative fold changes compared to control. (D) Immunoblot analysis of FAS, CPT1, CPT2, and PPARα. Protein band intensities are normalized to β-actin and expressed as ratios. Immunoblot data are presented as box-and-whisker plots showing the median, interquartile range, and full data range. One-way ANOVA followed by Tukey's multiple-comparisons test; n = 4, *$p<0.05$, **$p<0.01$, ***$p<0.001$. CPT, carnitine palmitoyltransferase; FAS, fatty acid synthase; HFD, high-fat diet; ND, normal diet; PPAR, peroxisome proliferator-activated receptor.

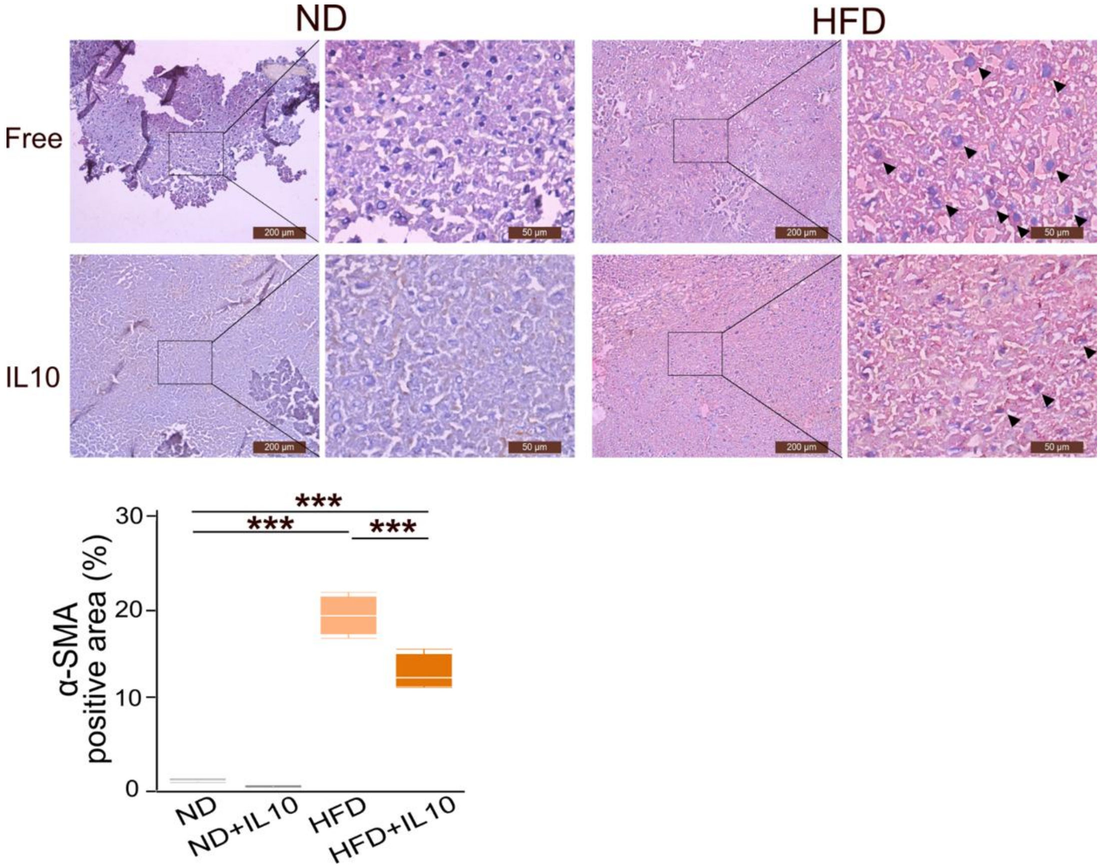

Fig. S5. IL-10 attenuates α-SMA–positive fibrogenic cell activation in HFD-fed mice. Representative liver sections from ND and HFD mice with or without IL-10 treatment are subjected to double immunostaining for IL-10Rα (DAB, brown) and α-SMA (Alkaline Phosphatase substrate, red), with hematoxylin counterstain (blue). Upper panel, no treatment; lower panel, IL-10 treatment. Stronger α-SMA staining is observed in the intracellular area of hepatocytes (arrowheads). α-SMA–positive area (%) is quantified using ImageJ in multiple fields per mouse and summarized as box-and-whisker plots. Scale bars = 200, 50 µm. HFD, high-fat diet; ND, normal diet. One-way ANOVA followed by Tukey's multiple-comparisons test; n = 4, *$p<0.05$, **$p<0.01$, ***$p<0.001$. HFD, high-fat diet; ND, normal diet.

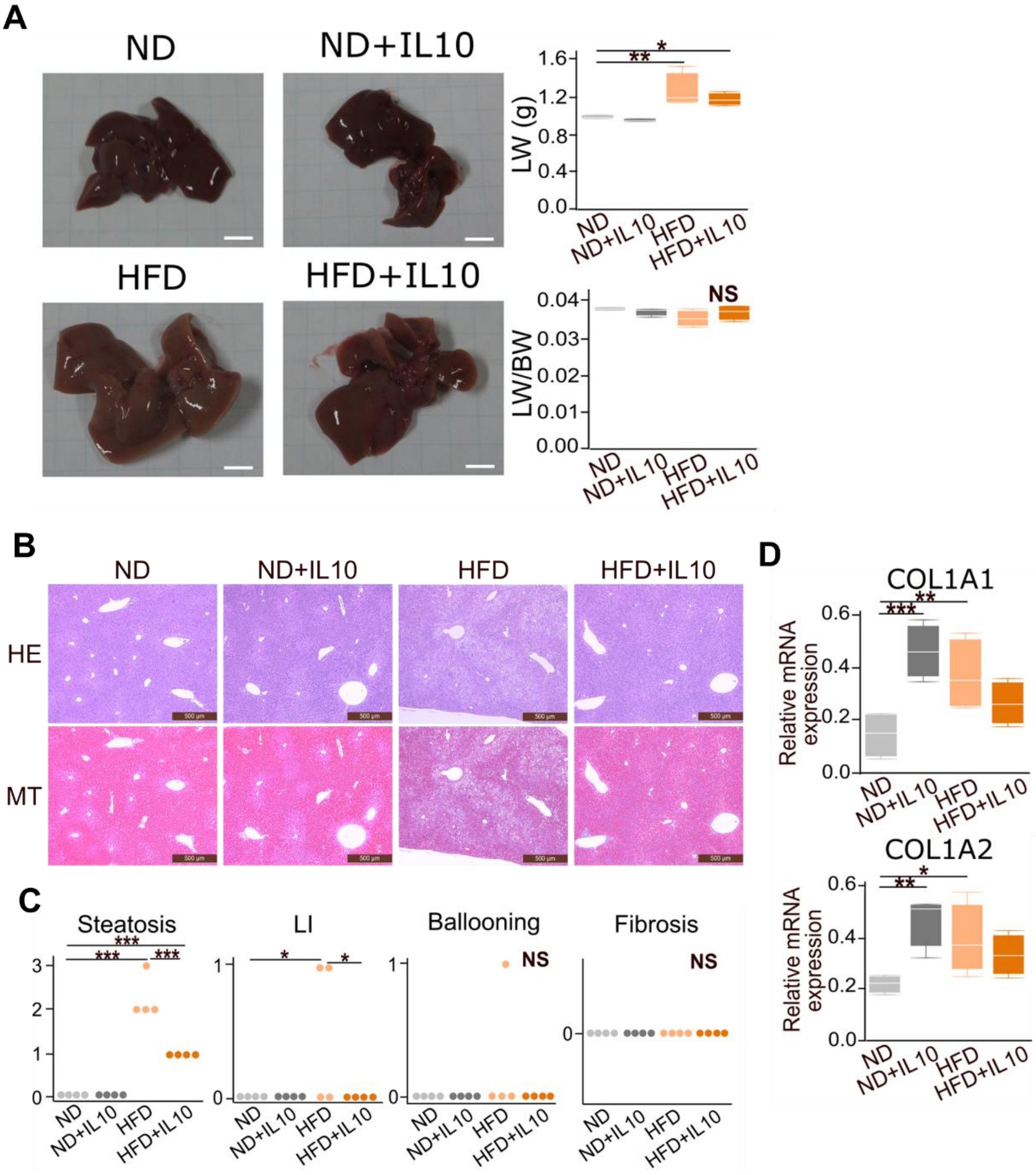

Fig. S6. Short-term IL-10 treatment provides hepatic histological improvement in HFD-fed mice. (A) Representative images of mouse livers from four experimental groups are captured after sacrifice. LWs in each group are recorded, and the LW/BW ratio is calculated. Scale bar = 5 mm. (B) HE and MT staining are performed to evaluate liver histology. Scale bar = 500 µm. (C) Hepatic steatosis, LI, hepatocyte ballooning, and fibrosis staging are analyzed. (D) mRNA expression levels in *COL1A1* and *COL1A2* are examined. They are quantified and normalized to *GAPDH*. Data are presented as box-and-whisker plots showing the median, interquartile range, and full data range. One-way ANOVA followed by Tukey's multiple-comparisons test; n = 4, **p*<0.05, ***p*<0.01, ****p*<0.001. BW, body weight; HFD, high-fat diet; LI, lobular inflammation; LW, liver weight; ND, normal diet.

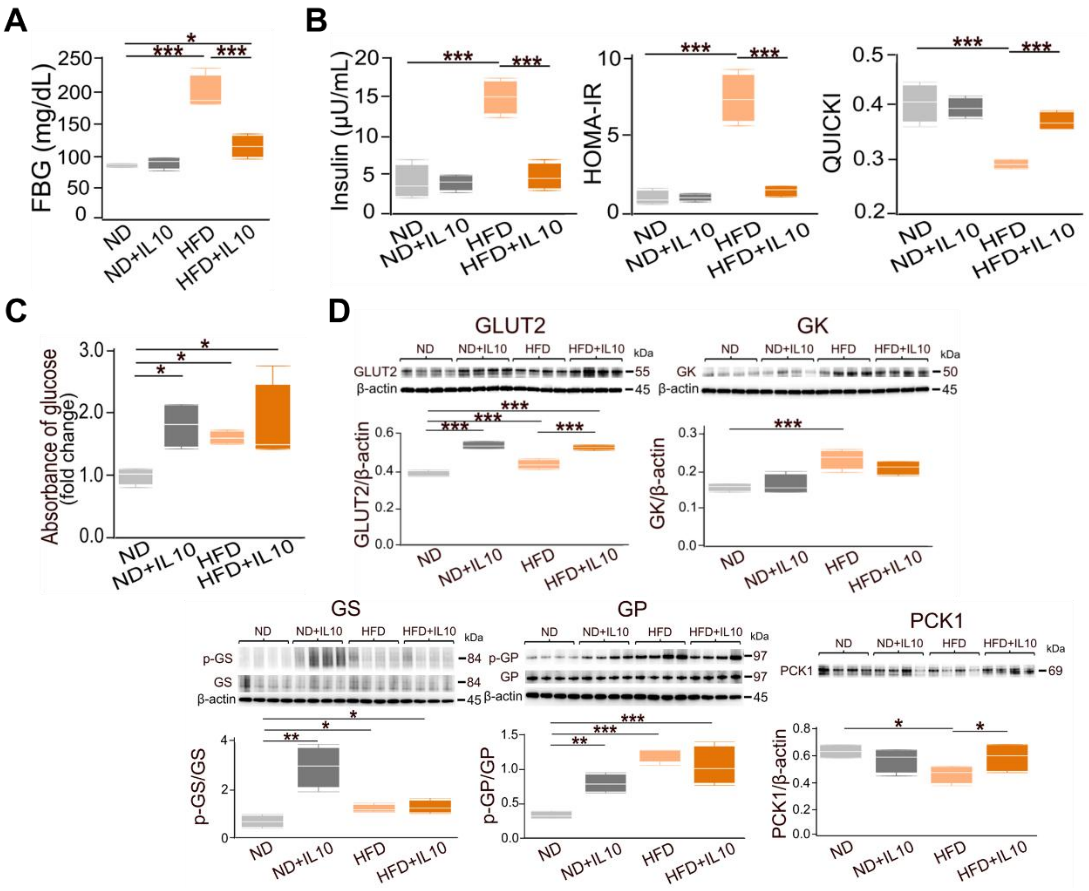

Fig. S7. Short-term IL-10 treatment also promotes hepatic glucose content in HFD-fed mice. (A) FBG levels are measured at sacrifice. (B) Fasting serum insulin levels, HOMA-IR, and QUICKI in each group. QUICKI is calculated from fasting glucose (G0) and insulin (I0) as QUICKI = 1/[log(I0) + log(G0)]. (C) Hepatic glucose content is quantified using absorption spectroscopy, and relative fold changes compared to the control are examined. (D) Immunoblot analysis of GLUT2, GK, GS, GP, and PCK1. β-actin loading control for GK was obtained from the same gel with PCK1. Protein band intensities are normalized to β-actin and expressed as ratios. Data are presented as box-and-whisker plots showing the median, interquartile range, and full data range. One-way ANOVA followed by Tukey's multiple-comparisons test; n = 4, **p*<0.05 versus ND-fed mice (control); #*p*<0.05 versus HFD-fed mice. GLUT2, glucose transporter 2; GK, glucokinase; GP, glycogen phosphorylase; GS, glycogen synthase; HFD, high-fat diet; HOMA-IR, homeostatic model assessment for IR; IR, insulin resistance; ND, normal diet; PCK1, phosphoenolpyruvate carboxykinase 1.

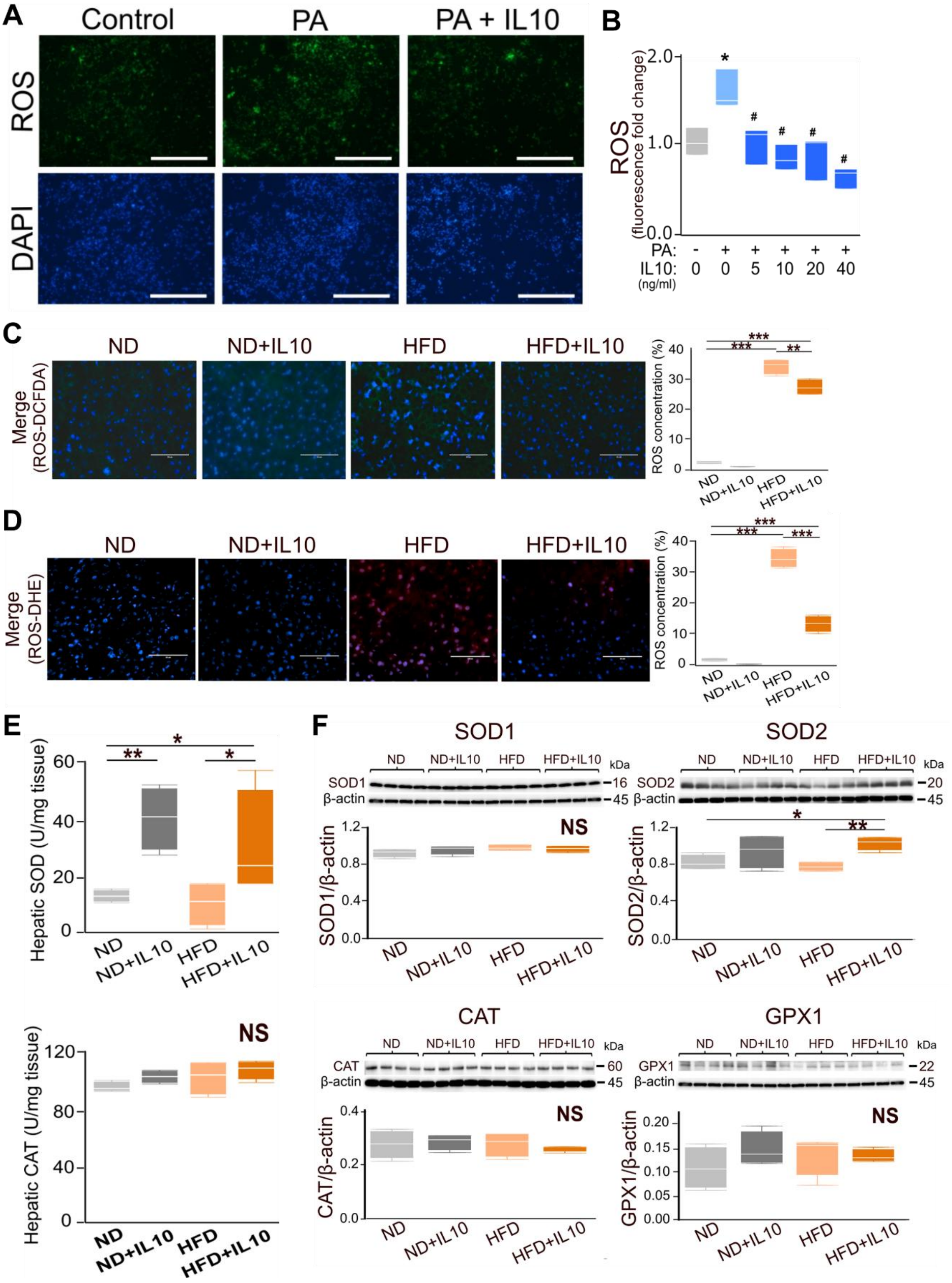

Fig. S8. Short-term IL-10 treatment suppresses hepatic oxidative stress in HepG2 cells. (A) FMI of GFP channel (green, ROS) and DAPI channel (blue) HepG2 cells. Immunocytochemistry is performed using an anti-GFP antibody for ROS detection, and subcellular localization following UV irradiation is captured using FMI. The upper panel displays raw images acquired in the GFP channel, while the lower panel shows the

corresponding DAPI channel. Scale bar = 1000 μm. (B) Cellular ROS accumulation is quantified using FMI, and relative fold changes compared to the control are examined. Data are presented as box-and-whisker plots showing the median, interquartile range, and full data range. One-way ANOVA followed by Tukey's multiple-comparisons test; n = 3, *p<0.05 versus non-PA and IL-10 0 ng/mL (control); #p<0.05 versus PA and IL-10 0 ng/mL. (C) FMI of the merged channel, a pseudo-colored overlay of GFP (green, ROS) and DAPI (blue) channels in liver tissues from four experimental groups. Immunohistochemistry is performed using an anti-GFP antibody for ROS detection, and subcellular localization following UV irradiation is captured using FMI. Scale bar = 50 μm. Hepatic ROS levels are quantitatively assessed using FMI, and the ROS-positive area (%) in each view is examined. The analysis is performed four times per group using different views. (D) FMI of the merged channel from the RFP (red, DHE) and DAPI (blue) channels, showing liver tissues from four experimental groups. Nuclear ROS localization is shown with red fluorescence. Scale bar = 50 μm. Quantitative evaluation of ROS is as described above. (E) Hepatic total SOD and catalase activities measured in liver homogenates (normalized to tissue/protein as indicated). Data are expressed as U/mg tissue. (F) Immunoblot analysis of SOD1, SOD2, CAT, and GPX. Protein band intensities are normalized to β-actin and expressed as ratios. Data are presented as box-and-whisker plots showing the median, interquartile range, and full data range. One-way ANOVA followed by Tukey's multiple-comparisons test; n = 4, *p<0.05, **p<0.01, ***p<0.001. CAT, catalase; GPX, glutathione peroxidase; HFD, high-fat diet; ND, normal diet; ROS, reactive oxygen species; SOD, superoxide dismutase.

150

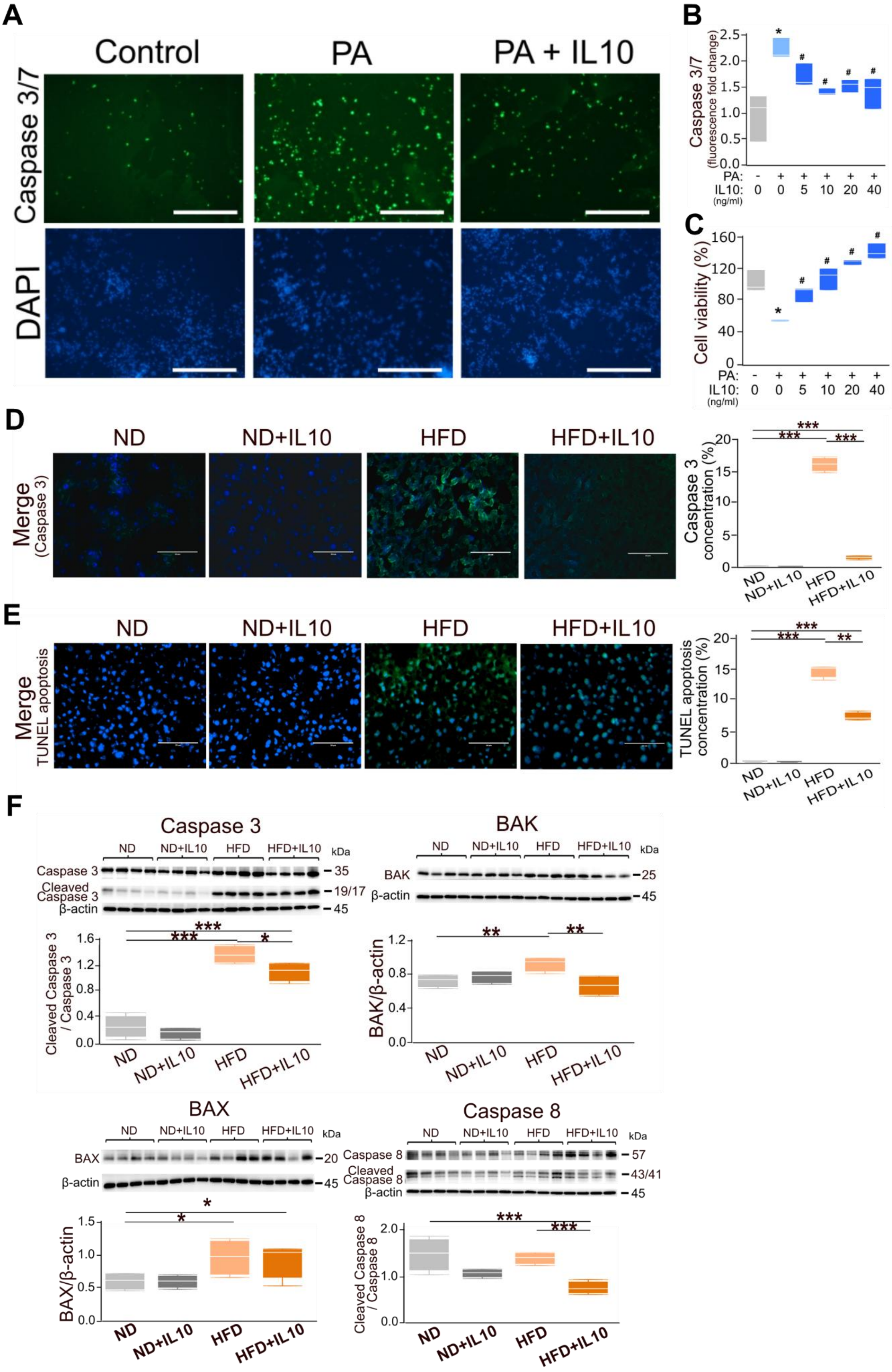
A
Control
PA
PA + IL10
Caspase 3/7
DAPI
B
Caspase 3/7 (fluorescence fold change)
PA: - + + + + +
IL10: 0 0 5 10 20 40
(ng/ml)
C
Cell viability (%)
PA: - + + + + +
IL10: 0 0 5 10 20 40
(ng/ml)
D
ND
ND+IL10
HFD
HFD+IL10
Merge (Caspase 3)
Caspase 3 concentration (%)
E
Merge TUNEL apoptosis
TUNEL apoptosis concentration (%)
F
Caspase 3
Cleaved Caspase 3
β-actin
Cleaved Caspase 3 / Caspase 3
BAK
BAK/β-actin
BAX
BAX/β-actin
Caspase 8
Cleaved Caspase 8
Cleaved Caspase 8 / Caspase 8
kDa

Fig. S9. Short-term IL-10 treatment suppresses hepatic apoptosis in HepG2 cells (A–C) and HFD-fed mice (D–F). (A) FMI of GFP channel (green, caspase-3/7) and DAPI channel (blue) in HepG2 cells. Immunocytochemistry and analysis are performed. The upper panel displays raw images acquired in the GFP channel, while the lower panel shows the corresponding DAPI channel. Scale bar = 1000 μm. (B) Cellular caspase-3/7 activity is quantified using FMI, and relative fold changes compared to the control are examined. (C) Cell viability, representing energy metabolic activity, is quantified using absorption spectroscopy with XTT. Relative fold changes compared to the control (100%) are examined. Data are presented as box-and-whisker plots showing the median, interquartile range, and full data range. One-way ANOVA followed by Tukey's multiple-comparisons test; n = 3, *p<0.05 versus non-PA and IL-10 0 ng/mL (control); #p<0.05 versus PA and IL-10 0 ng/mL. (D) FMI of the merged channel, a pseudo-colored overlay of GFP (green, cleaved caspase-3) and DAPI (blue) channels in liver tissues from four experimental groups. Immunohistochemistry is performed using an anti-GFP antibody for caspase-3 detection. Scale bar = 50 μm. Hepatic caspase-3 activity is quantified using FMI, and the caspase-3-positive area (%) in each view is examined. The analysis is performed four times per group using different views. (E) FMI of the merged channel from the GFP (green, TUNEL) and DAPI (blue) channels, showing liver tissues from four experimental groups. Nuclear DNA fragmentation as a marker of late-stage apoptosis is shown with green fluorescence. Scale bar = 50 μm. Quantitative evaluation of apoptosis is as described above. (F) Immunoblot analysis of BAK, BAX, and cleaved caspase-8/caspase-8 ratio. β-actin loading control for BAK was obtained from the same gel with cleaved caspase-8/caspase-8. Protein band intensities are normalized to β-actin and expressed as ratios. Data are presented as box-and-whisker plots showing the median, interquartile range, and full data range. One-way ANOVA followed by Tukey's multiple-comparisons test; n = 4, *p<0.05, **p<0.01, ***p<0.001. BAK, Bcl-2 antagonist or killer; BAX, Bcl-2-associated X protein; HFD, high-fat diet; ND, normal diet.

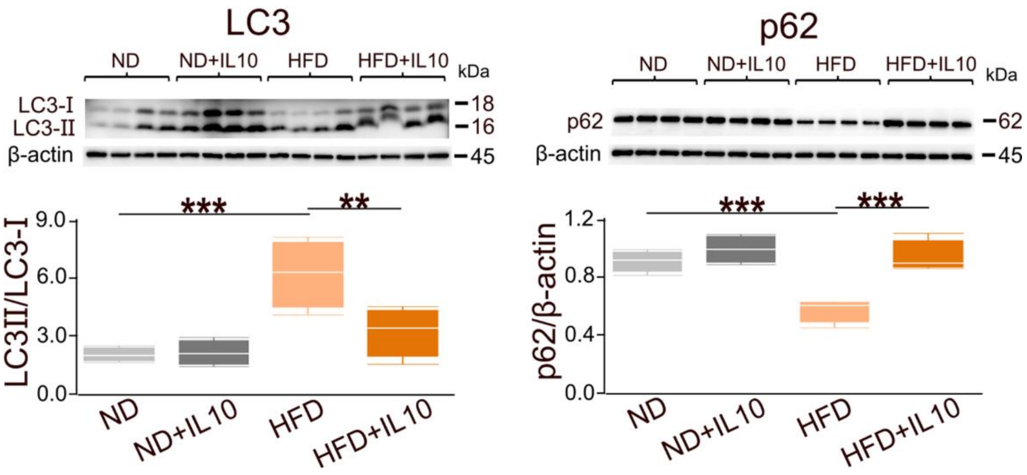

Fig. S10. IL-10 modulates autophagy-related markers *in vivo*. Immunoblot analysis of LC3 (LC3-I/LC3-II) and p62 in liver tissues from ND, ND+IL-10, HFD, and HFD+IL-10 groups. LC3-II/I ratio and p62 protein levels are quantified (normalized to β-actin) and expressed relative to ND controls. Data are presented as box-and-whisker plots showing the median, interquartile range, and full data range. One-way ANOVA followed by Tukey's multiple-comparisons test; n = 4, *p<0.05, **p<0.01, ***p<0.001. HFD, high-fat diet; ND, normal diet.
