# Supplementary material for: IL-10 attenuates metabolic dysfunction–associated steatotic liver disease via modulation of hepatic responses to lipotoxicity
Source: JCI Insight. 2026 Apr 23;11(12):e200231. doi: 10.1172/jci.insight.200231 (PMC13313487; doi:10.1172/jci.insight.200231)
Supplement: Unedited blot and gel images [file jciinsight-11-200231-s071.pdf]

Fig. 1G (IL-10Rα Full unedited blot)

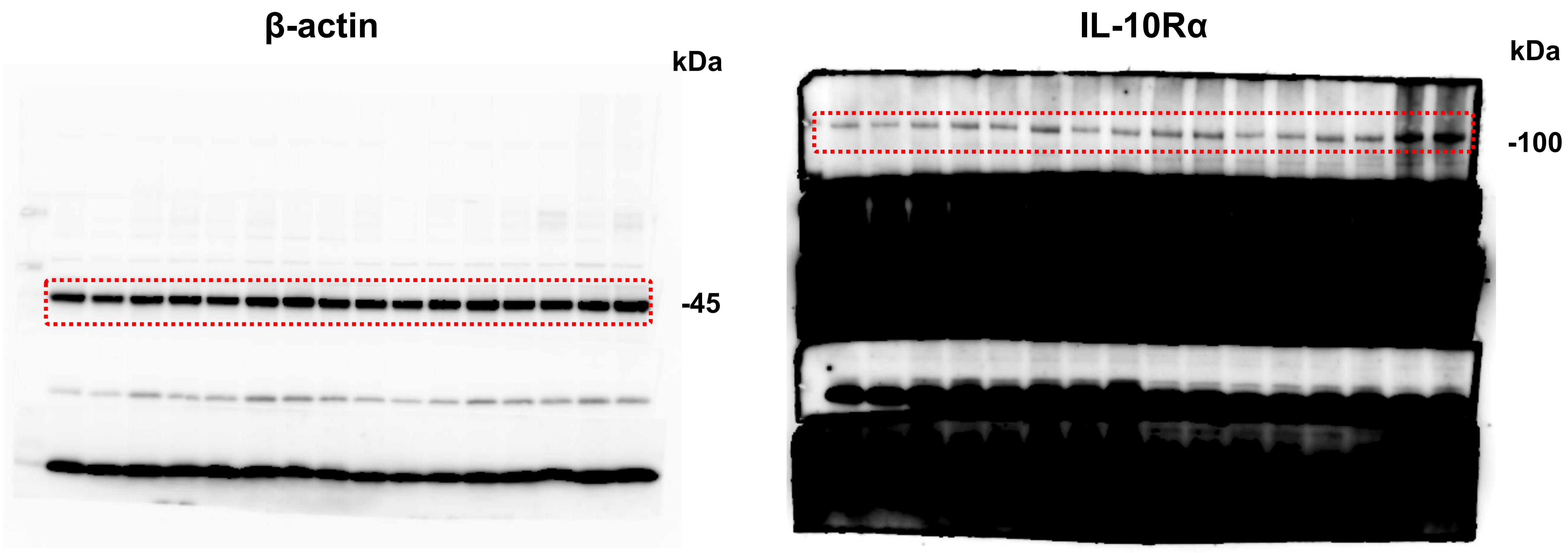

Note: 1. The same blot and different exposure times (short and long exposures shown) were used.
2. Bands used in Figures are indicated by boxes in red outlines.

Fig. 1H (FAS-Full unedited blot)

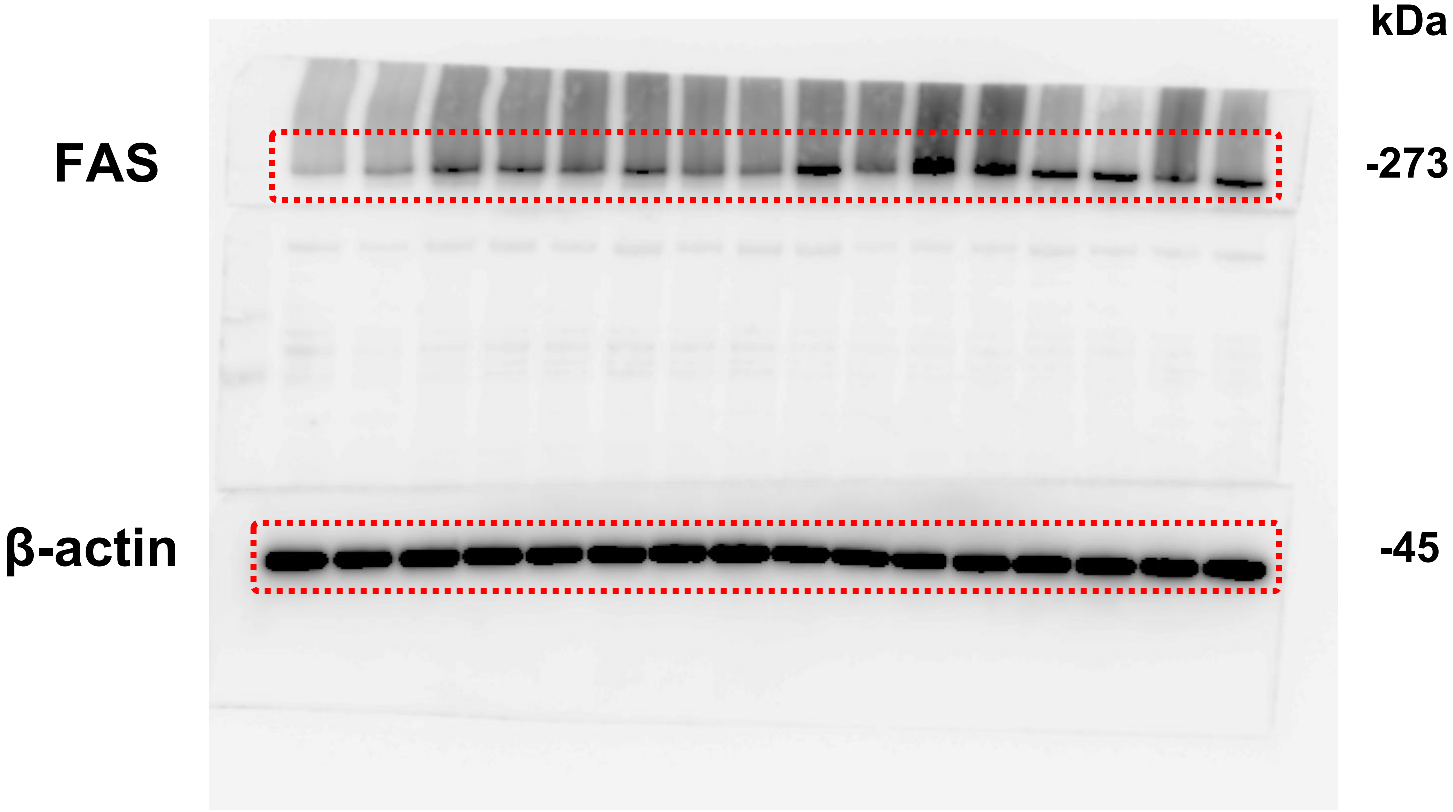

Note: 1. The same blot was used.
2. Bands used in Figures are indicated by boxes in red outlines.

Fig. 1H (CPT1-Full unedited blot)

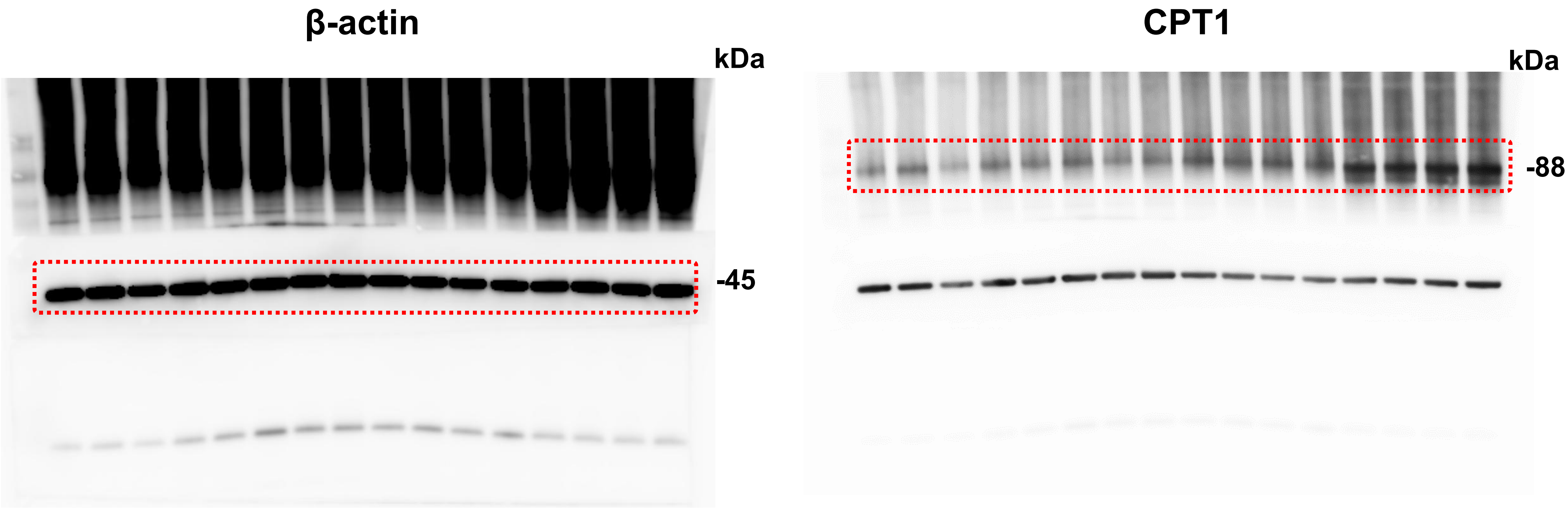

Note: 1. The same blot and different exposure times (short and long exposures shown) were used.
2. Bands used in Figures are indicated by boxes in red outlines.

Fig. 1H (CPT2-Full unedited blot)

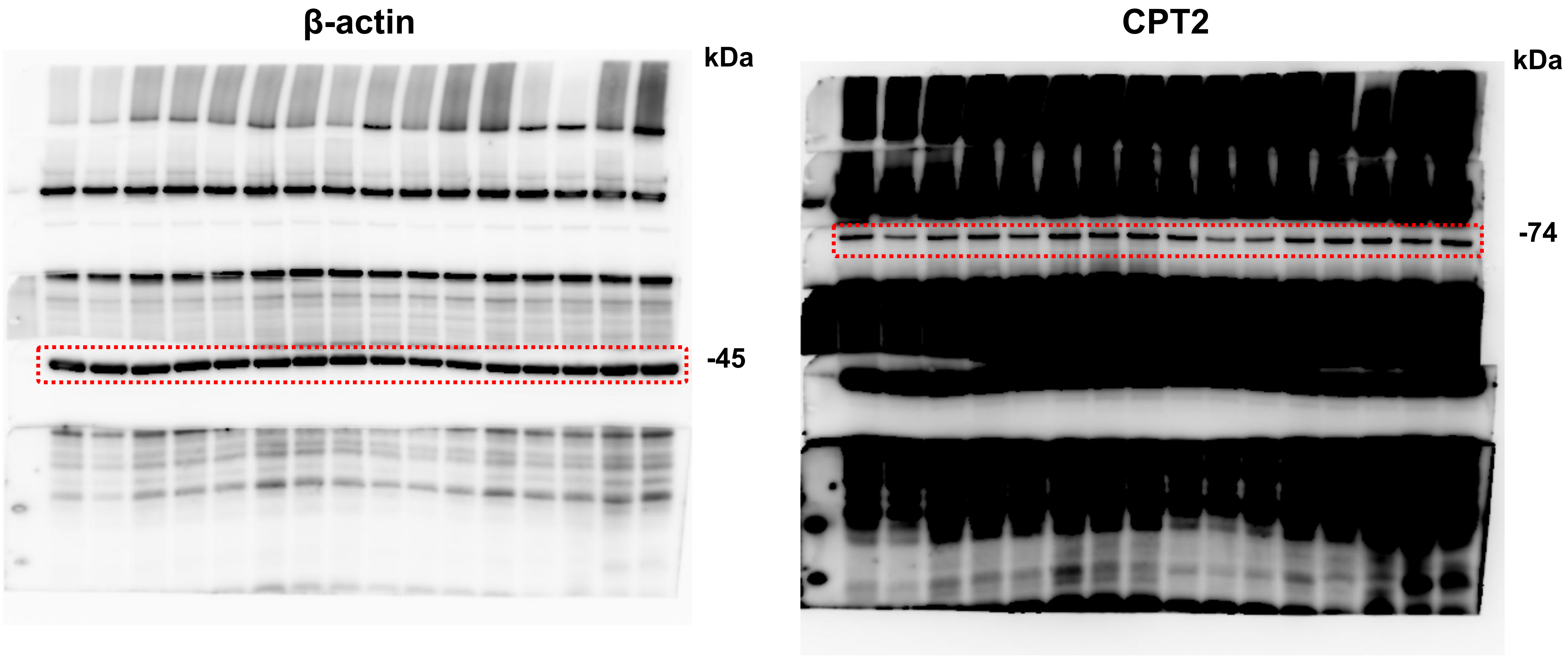

Note: 1. The same blot and different exposure times (short and long exposures shown) were used.
2. Bands used in Figures are indicated by boxes in red outlines.

Fig. 1H (PPARα-Full unedited blot)

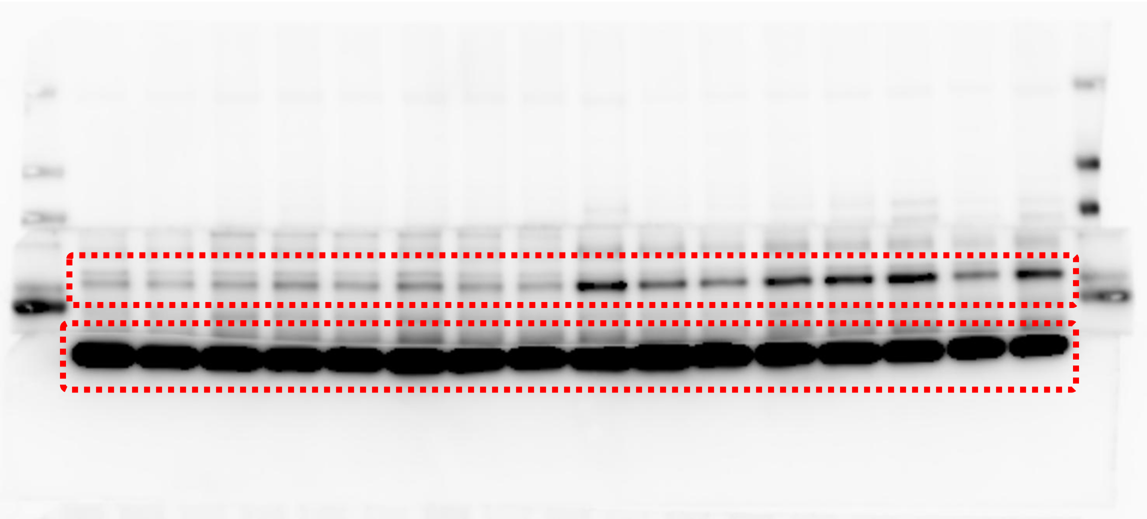

Note: 1. The same blot was used.
2. Bands used in Figures are indicated by boxes in red outlines.

Fig. 2C (FAS-Full unedited blot)

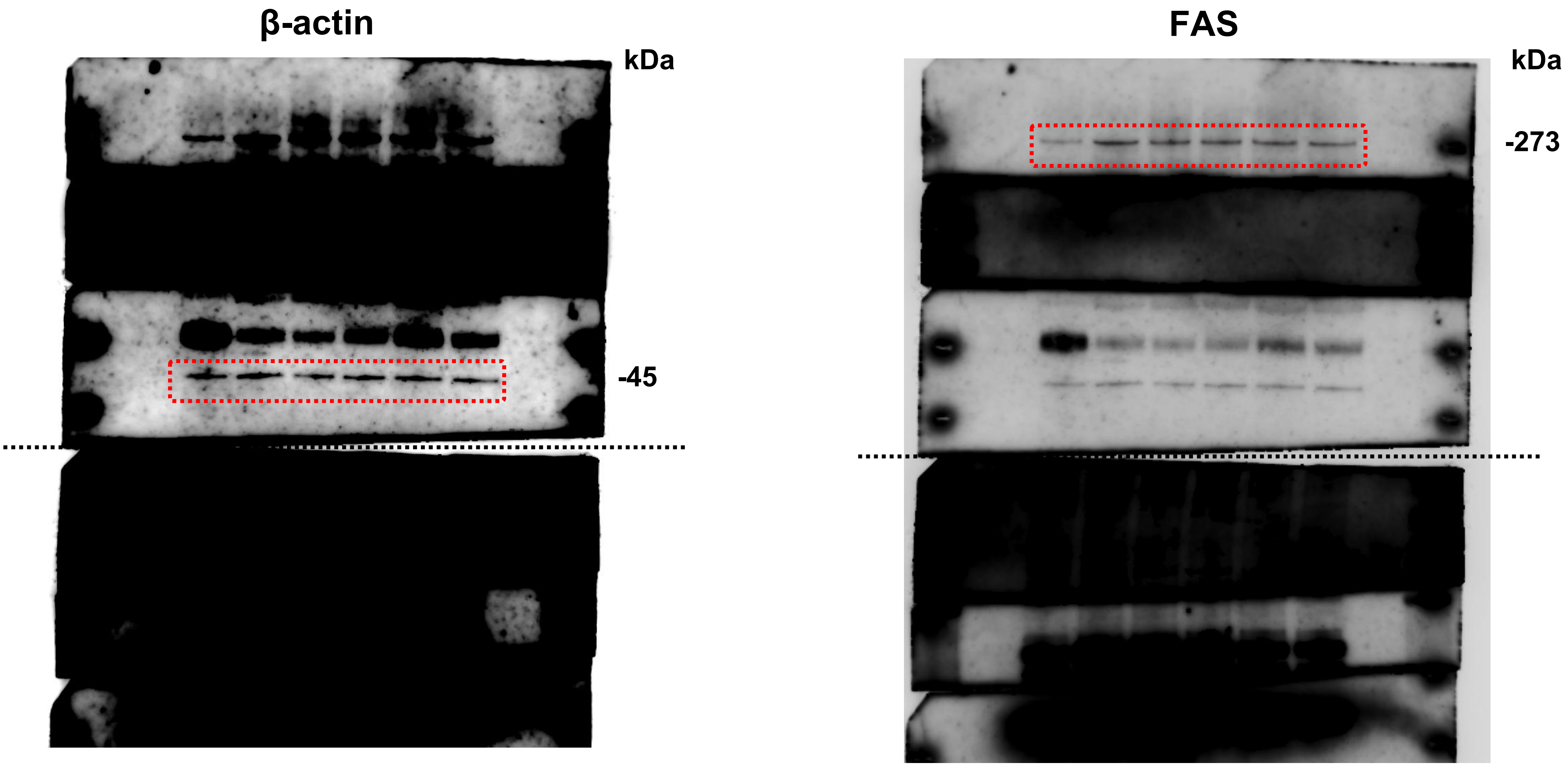

Note: 1. The same blot and different exposure times (short and long exposures shown) were used.
2. Bands used in Figures are indicated by boxes in red outlines.

Fig. 2C (CPT1-Full unedited blot)

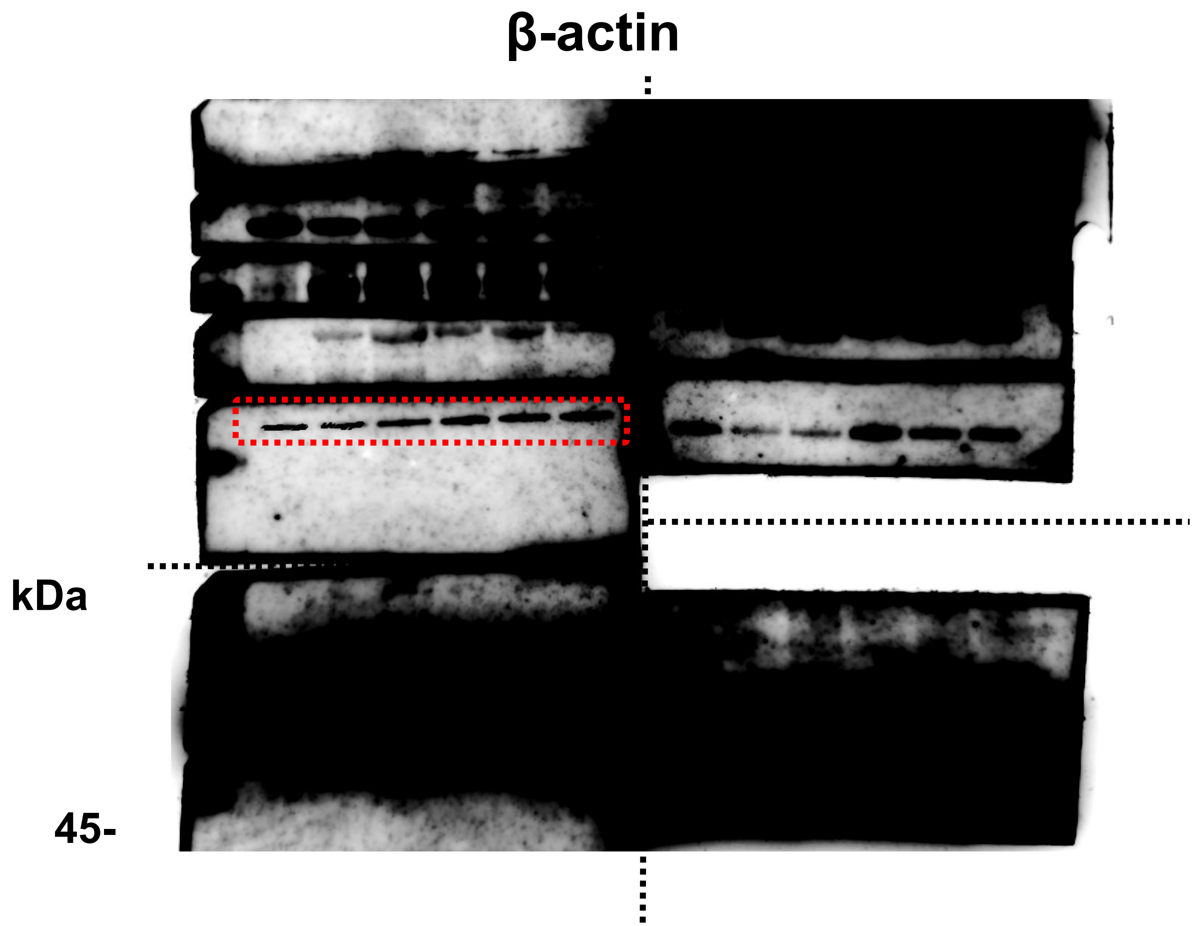

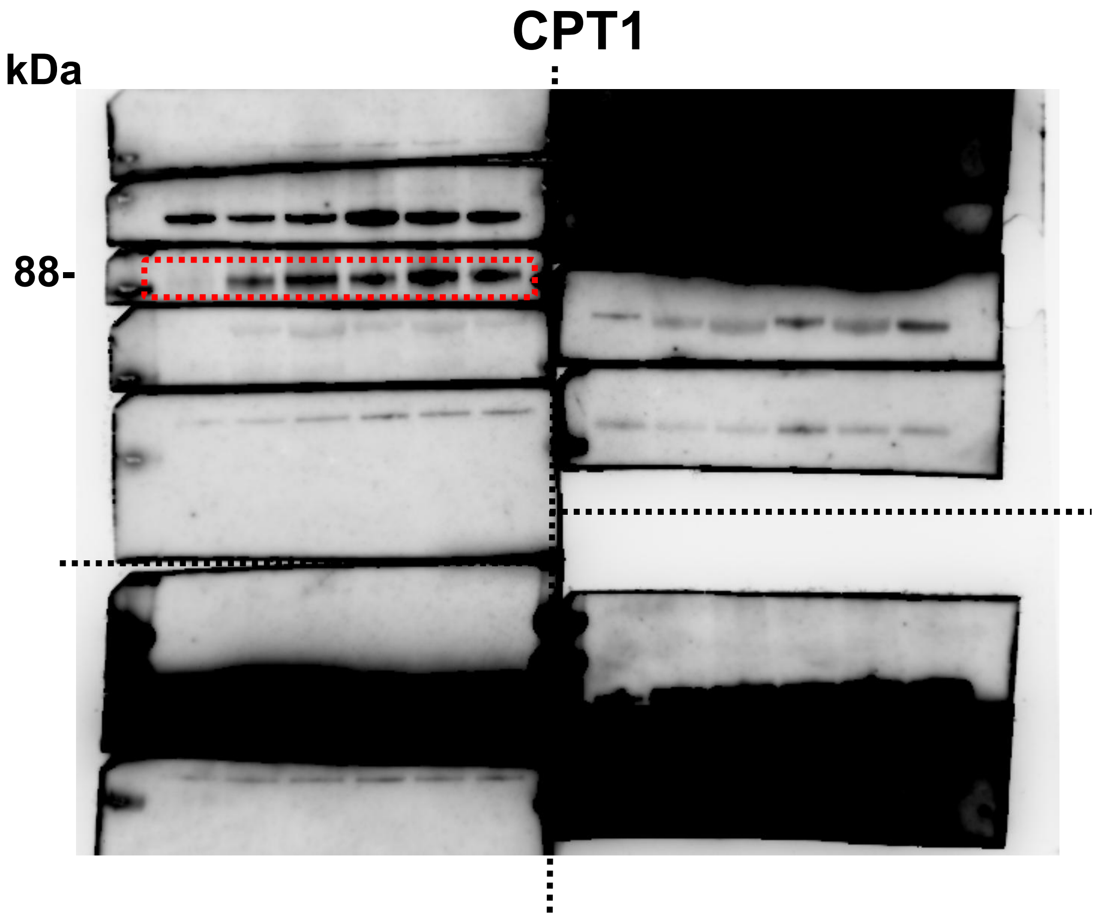

Note: 1. The same blot and different exposure times (short and long exposures shown) were used.
2. Bands used in Figures are indicated by boxes in red outlines.

Fig. 2C (CPT2-Full unedited blot)

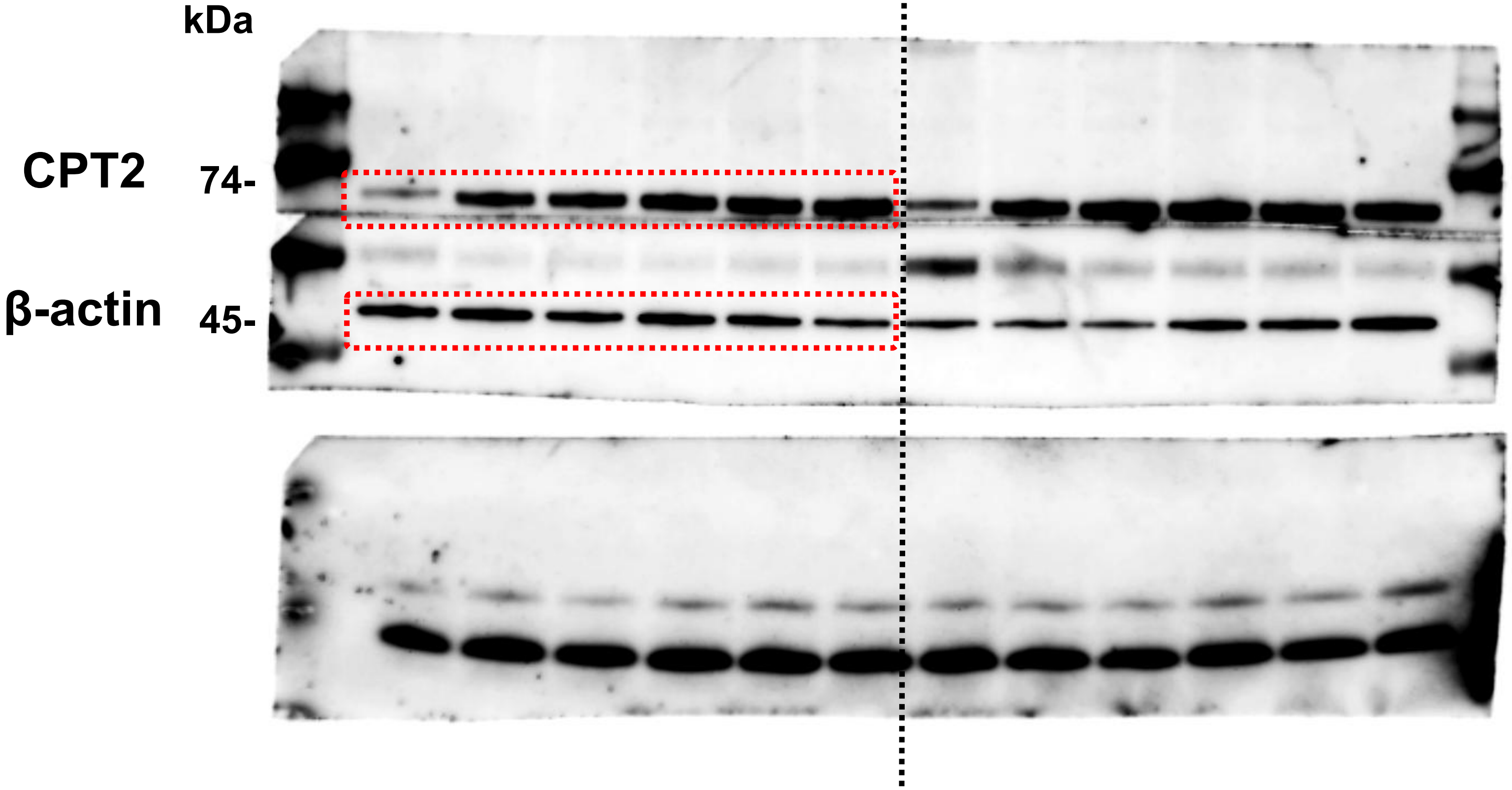

Note: 1. The same blot was used.
2. Bands used in Figures are indicated by boxes in red outlines.

Fig. 2C (PPARα-Full unedited blot)

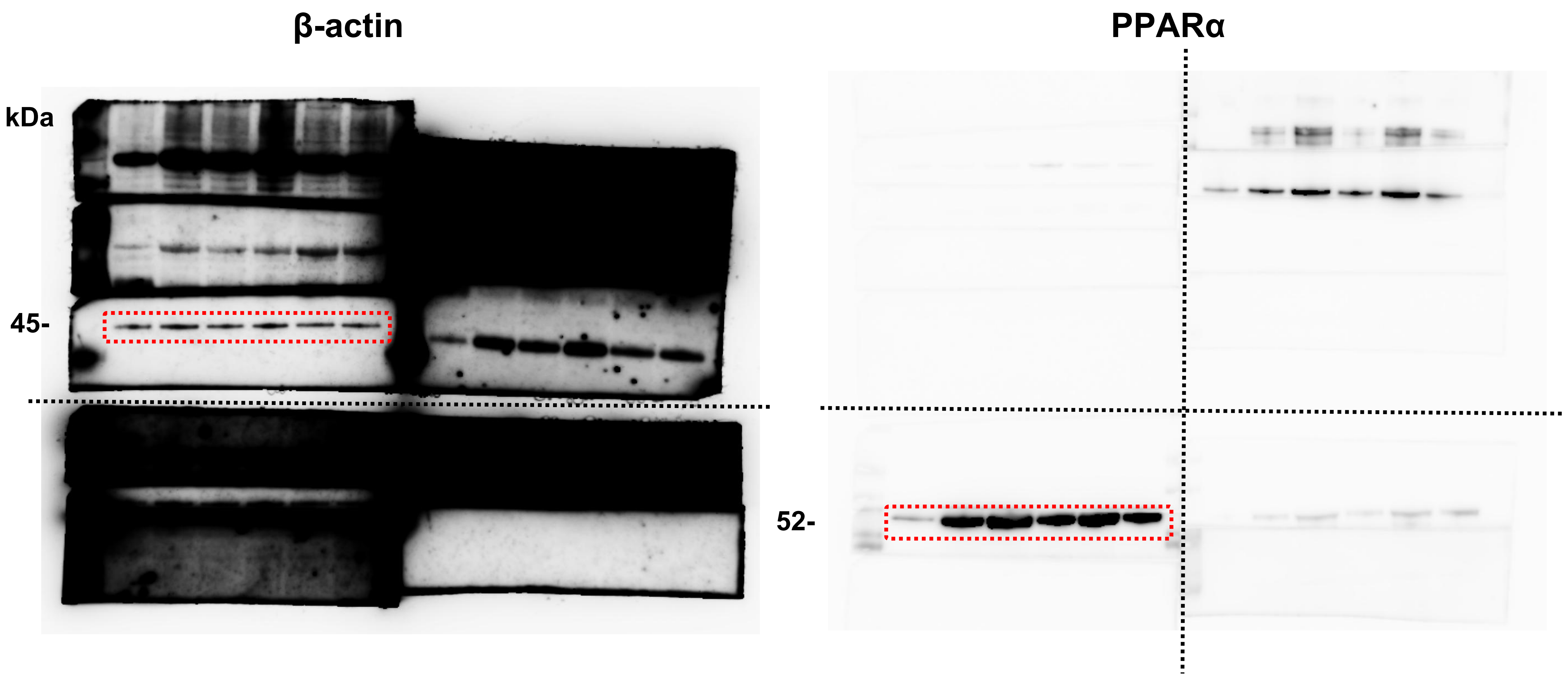

Note: 1. Separate, noncontiguous lanes from the same samples were used for each antibody.
2. Bands used in Figures are indicated by boxes in red outlines.

Fig. 4D (GLUT2-Full unedited blot)

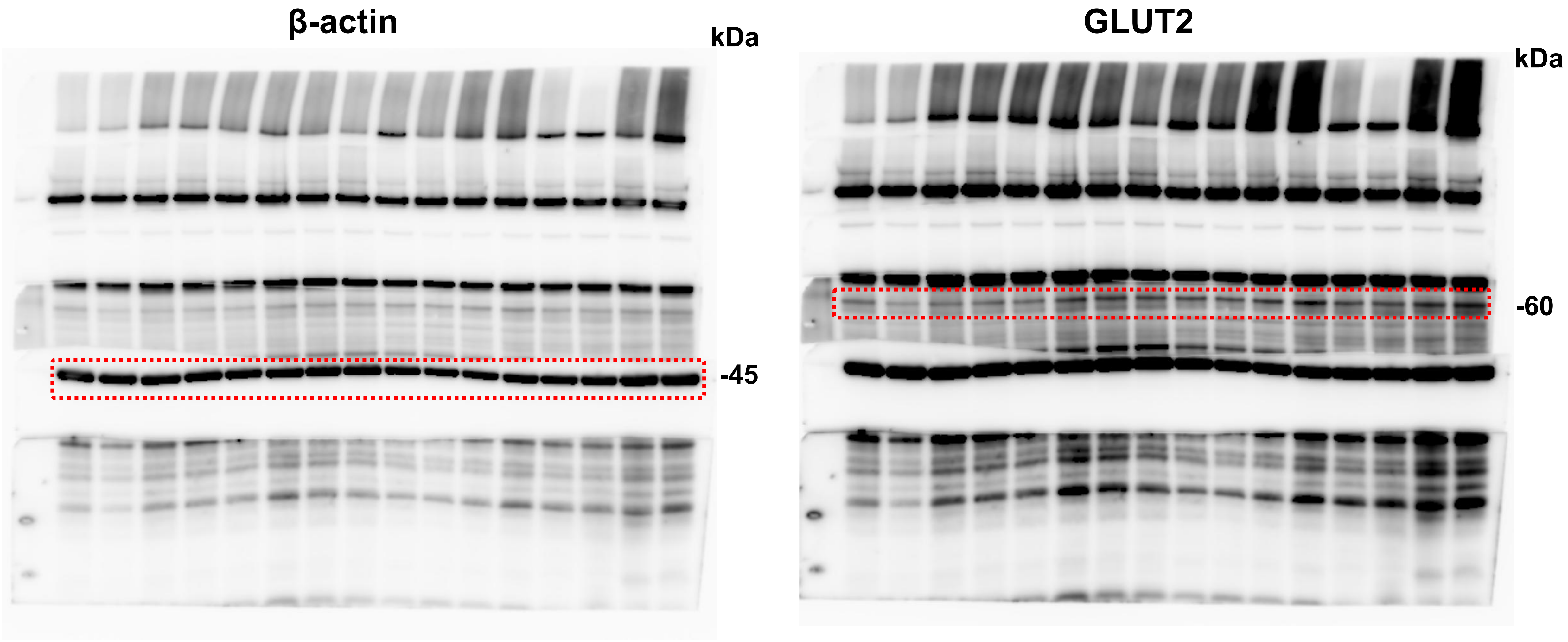

Note: 1. The same blot and different exposure times (short and long exposures shown) were used.
2. Bands used in Figures are indicated by boxes in red outlines.

Fig. 4D (GK-Full unedited blot)

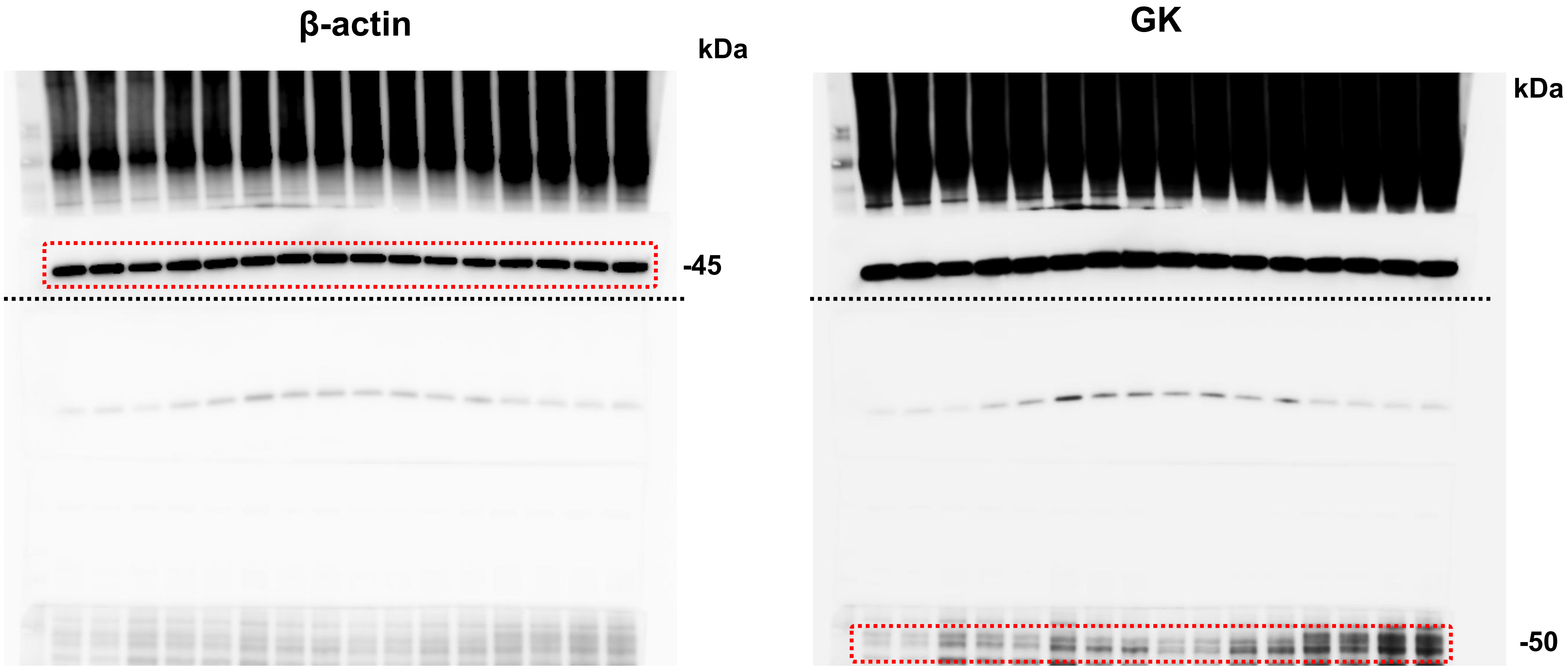

Note: 1. Separate, noncontiguous lanes from the same samples were used for each antibody.
2. Bands used in Figures are indicated by boxes in red outlines.

Fig. 4D (p-GS-GS-Full unedited blot)

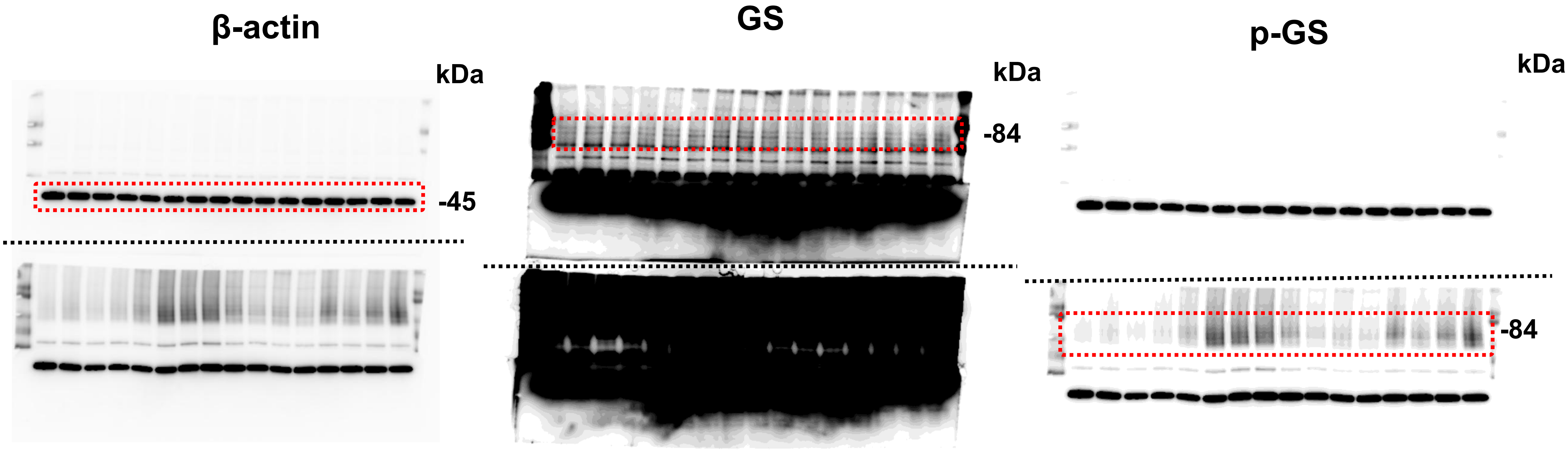

Note: 1. In GS and β-actin expression, the same blot and different exposure times (short and long exposures shown) were used. Between p-GS and GS expression, separate, noncontiguous lanes from the same samples were used for each antibody.
2. Bands used in Figures are indicated by boxes in red outlines.

Fig. 4D (p-GP-GP-Full unedited blot)

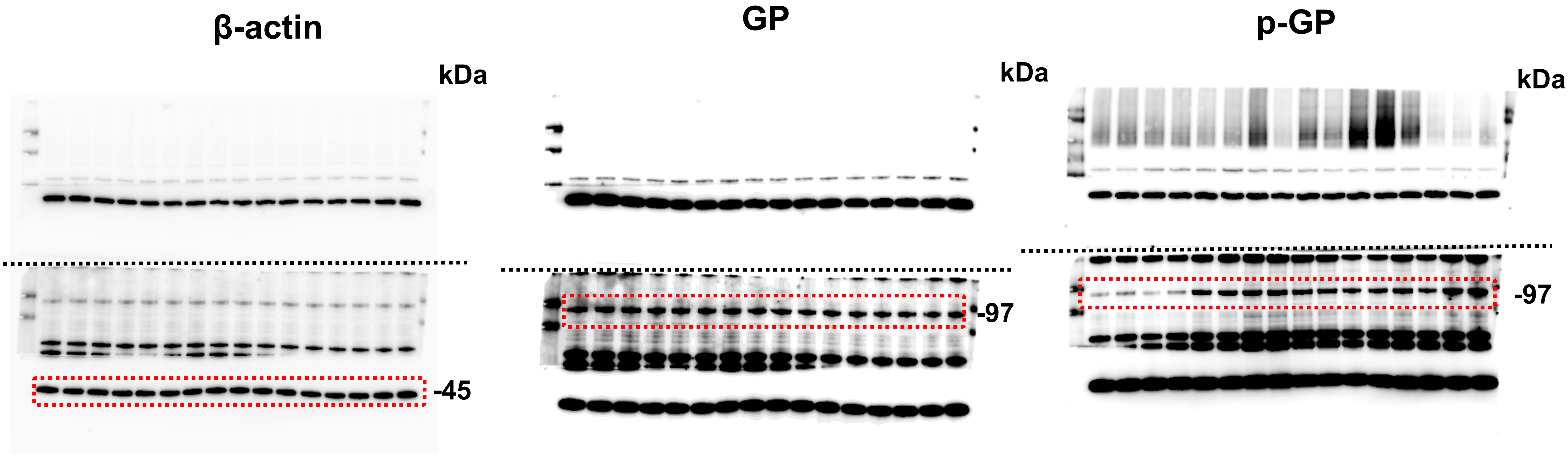

Note: 1. In GP and β-actin expression, the same blot and different exposure times (short and long exposures shown) were used. Between p-GP and GP expression, separate, noncontiguous lanes from the same samples were used for each antibody.
2. Bands used in Figures are indicated by boxes in red outlines.

Fig. 4D (PCK1-Full unedited blot)

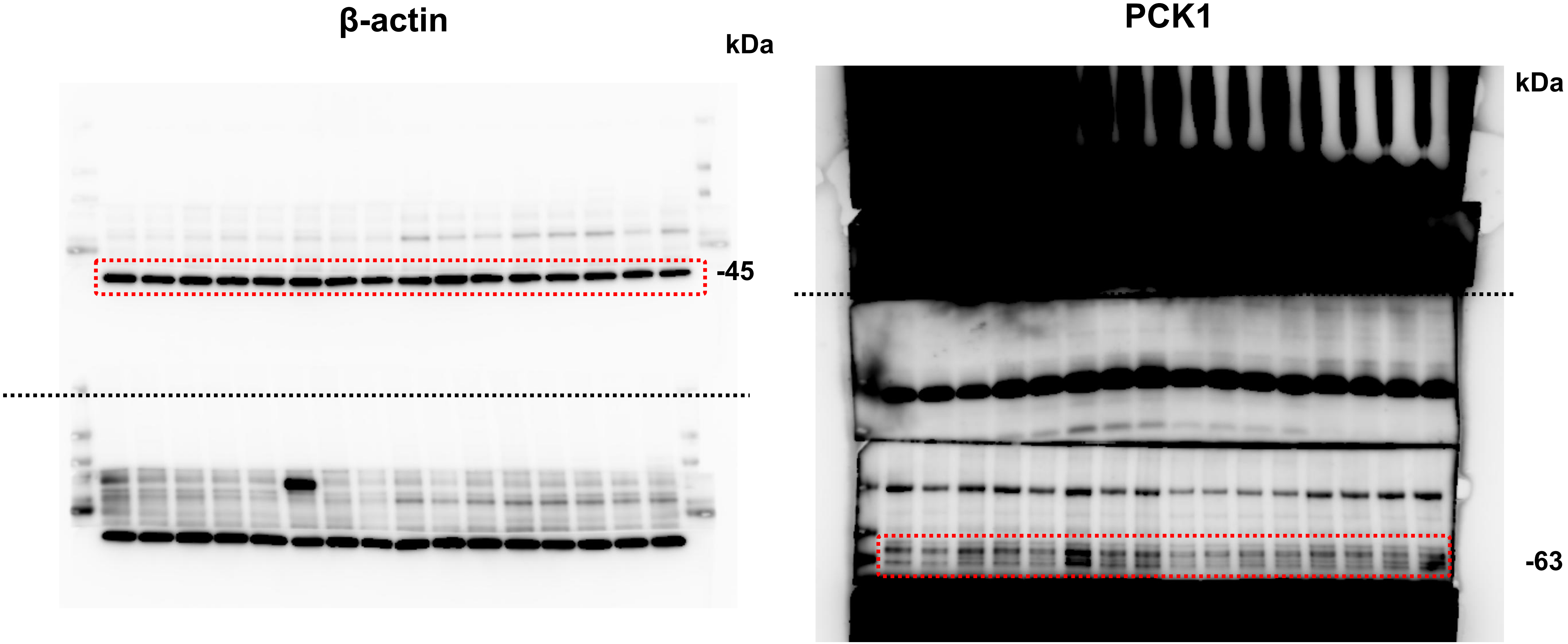

Note: 1. Separate, noncontiguous lanes from the same samples were used for each antibody.
2. Bands used in Figures are indicated by boxes in red outlines.

Fig. 4G (GLUT2-Full unedited blot)

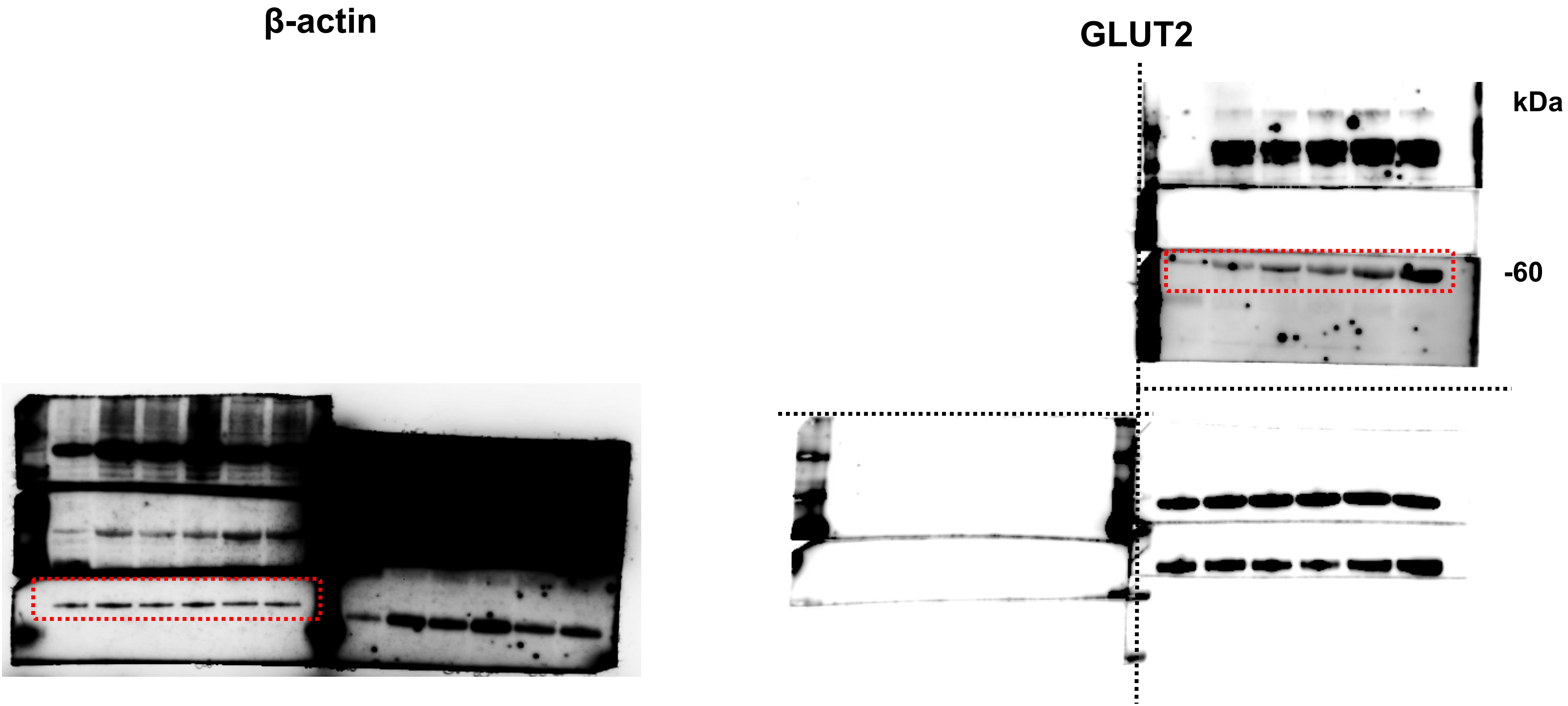

Note: 1. Separate, noncontiguous lanes from the same samples were used for each antibody.
2. Bands used in Figures are indicated by boxes in red outlines.

Fig. 4G (GK-Full unedited blot)

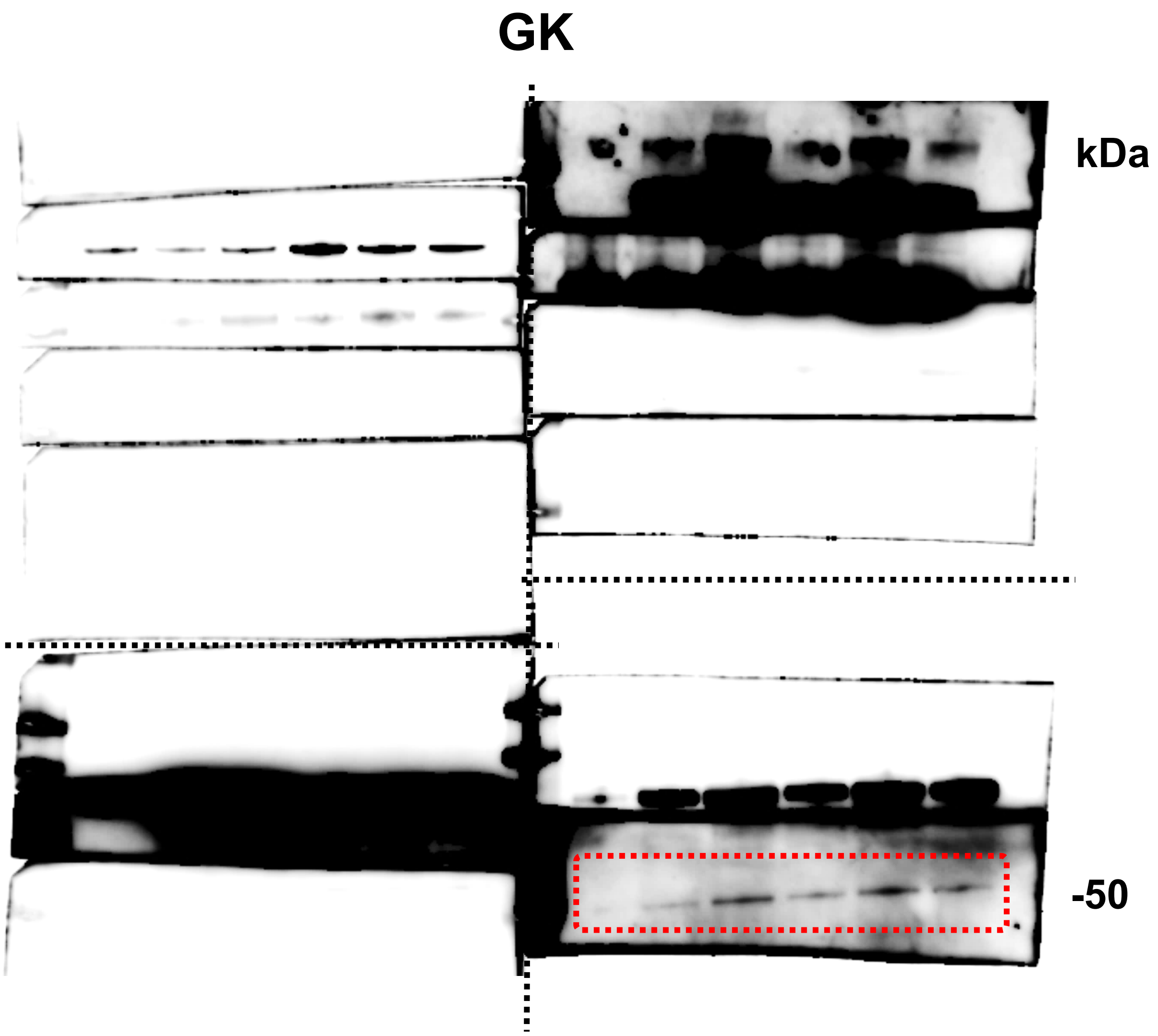

See Fig. 4G (GLUT2-Full unedited blot)

Note: 1. Separate, noncontiguous lanes from the same samples were used for each antibody.
β-actin loading control for GK was obtained from the same gel with GLUT2.
2. Bands used in Figures are indicated by boxes in red outlines.

Fig. 4G (p-GS-GS-Full unedited blot)

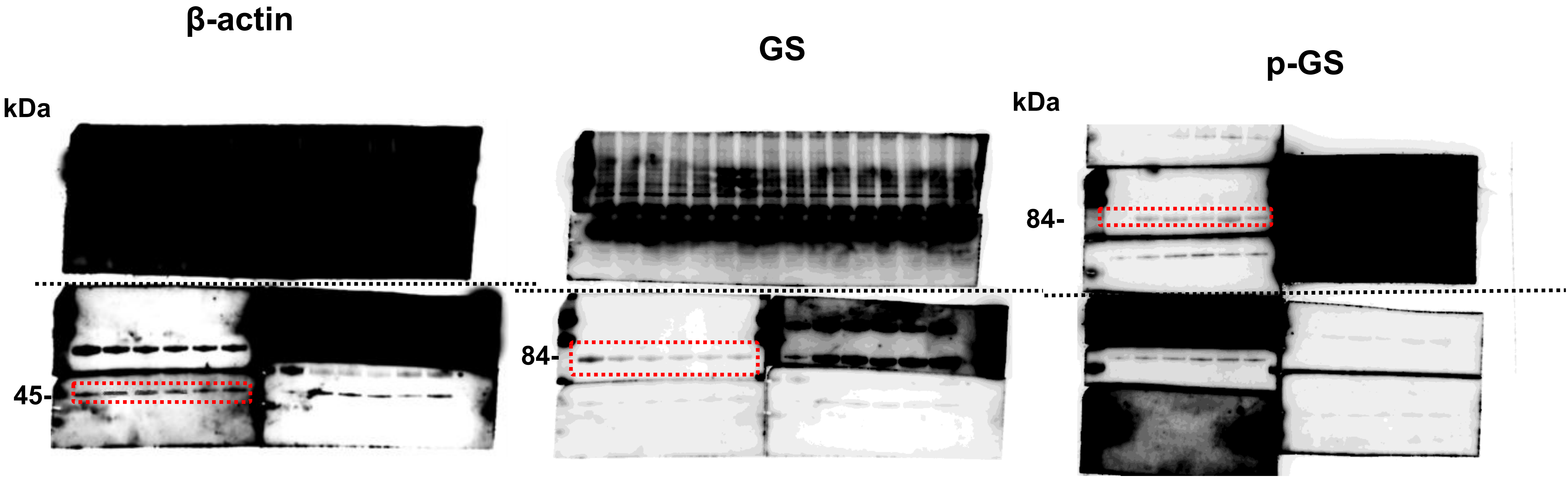

Note: 1. In GS and β-actin expression, the same blot and different exposure times (short and long exposures shown) were used. Between p-GS and GS expression, separate, noncontiguous lanes from the same samples were used for each antibody.
2. Bands used in Figures are indicated by boxes in red outlines.

Fig. 4G (p-GP-GP-Full unedited blot)

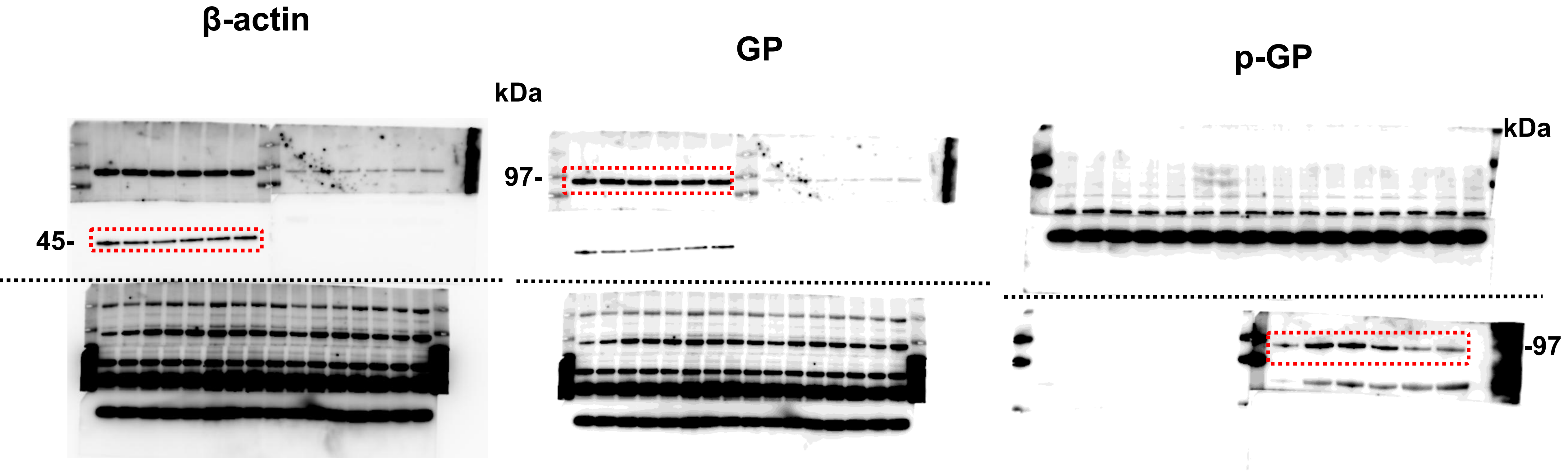

Note: 1. In GP and β-actin expression, the same blot and different exposure times (short and long exposures shown) were used. Between p-GP and GP expression, separate, noncontiguous lanes from the same samples were used for each antibody.
2. Bands used in Figures are indicated by boxes in red outlines.

Fig. 4G (PCK1-Full unedited blot)

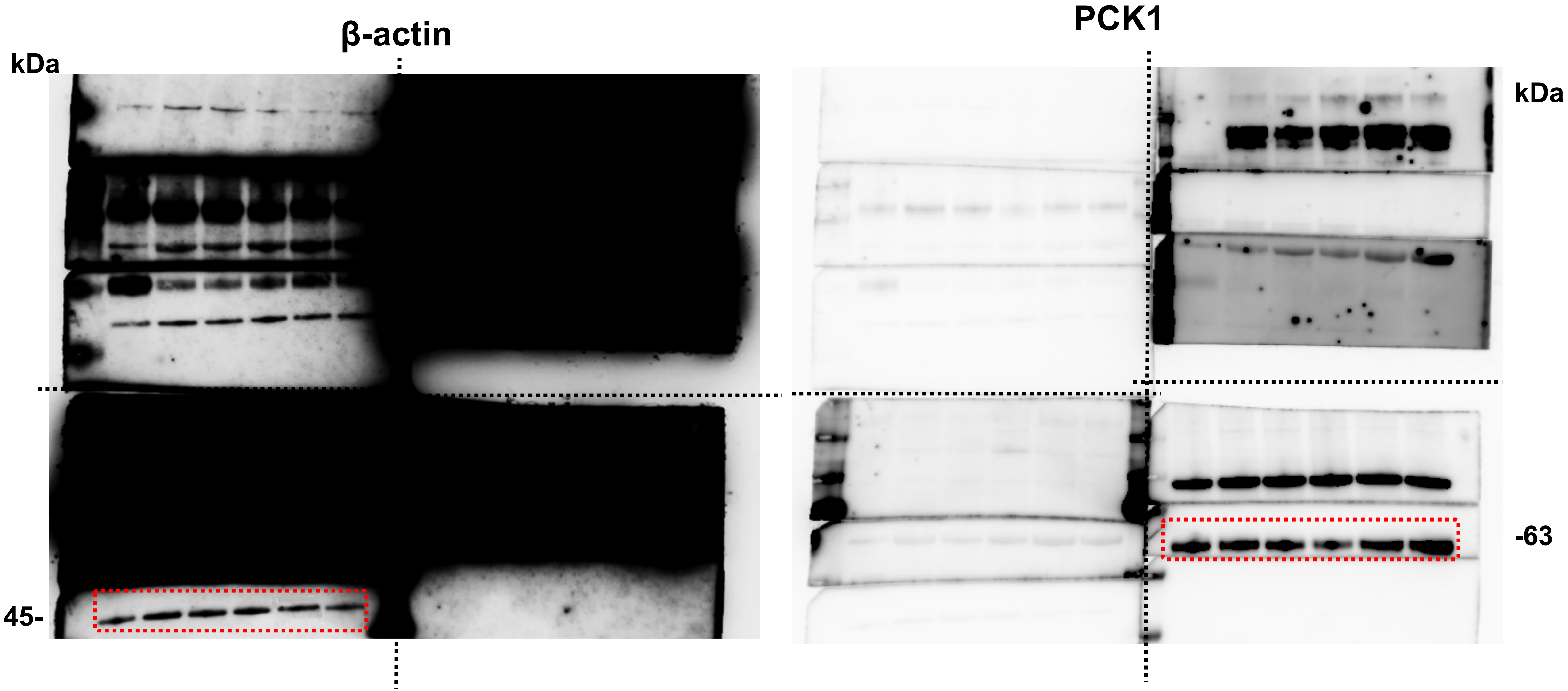

Note: 1. The same blot and different exposure times (short and long exposures shown) were used.
2. Bands used in Figures are indicated by boxes in red outlines.

Fig. 5D (SOD1-Full unedited blot)

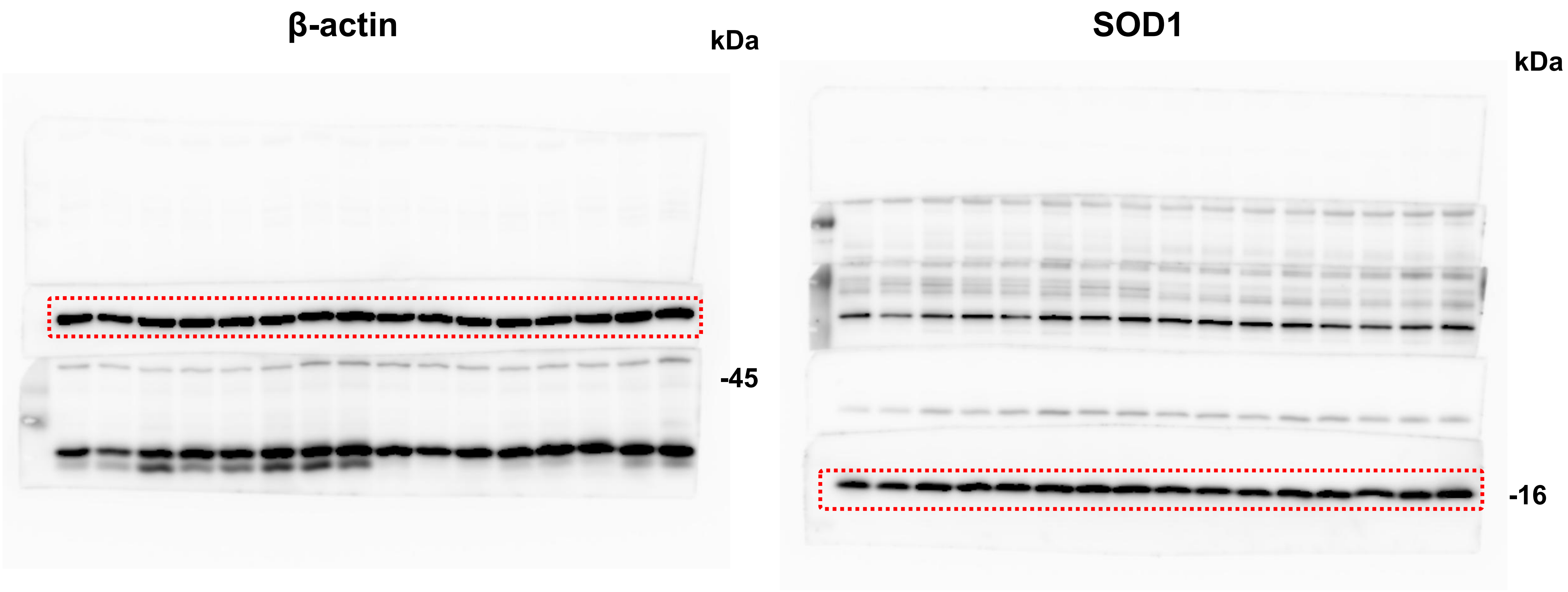

Note: 1. Separate, noncontiguous lanes from the same samples were used for each antibody.
2. Bands used in Figures are indicated by boxes in red outlines.

Fig. 5D (SOD2-Full unedited blot)

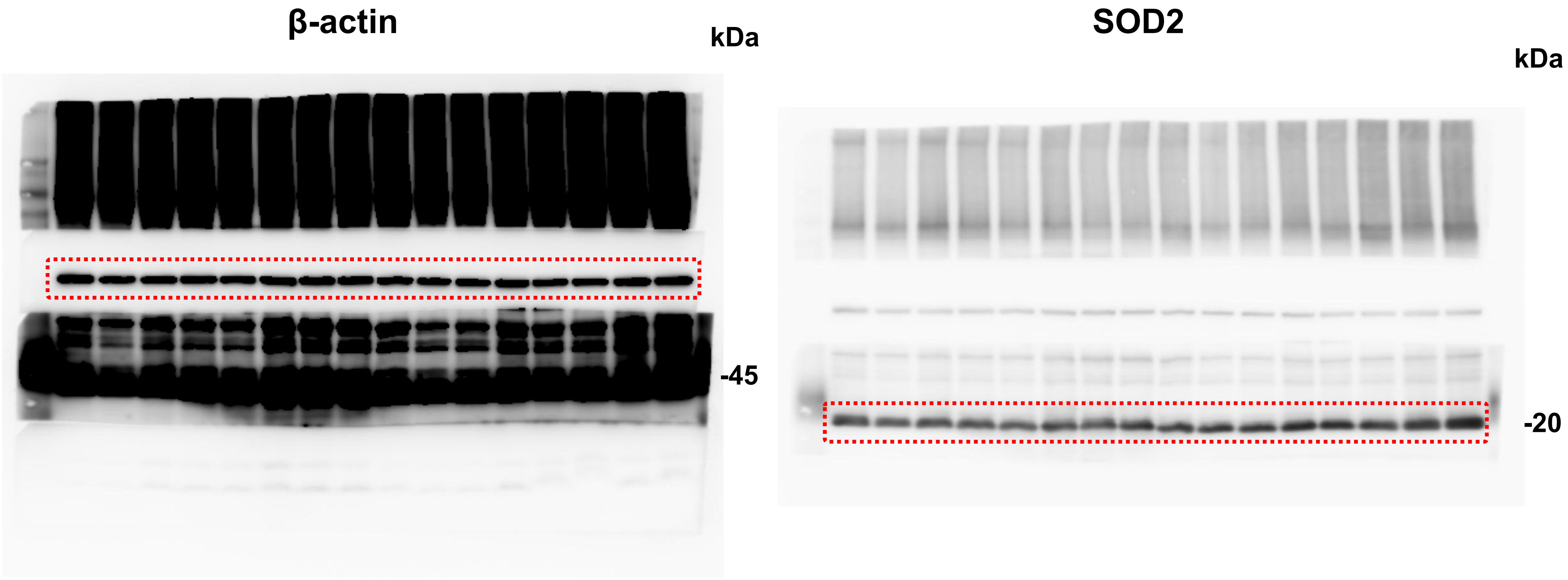

Note: 1. The same blot and different exposure times (short and long exposures shown) were used.
2. Bands used in Figures are indicated by boxes in red outlines.

Fig. 5D (CAT-Full unedited blot)

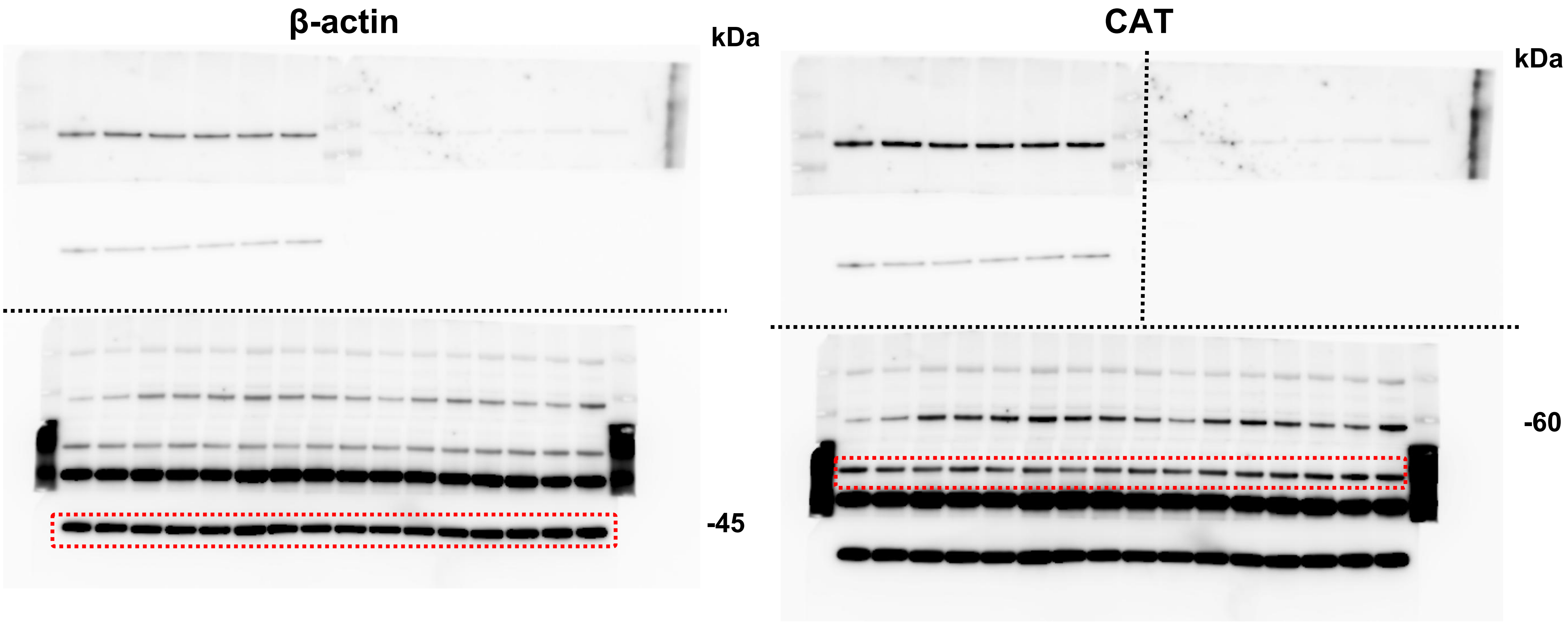

Note: 1. The same blot and different exposure times (short and long exposures shown) were used.
2. Bands used in Figures are indicated by boxes in red outlines.

Fig. 5D (GPX1-Full unedited blot)

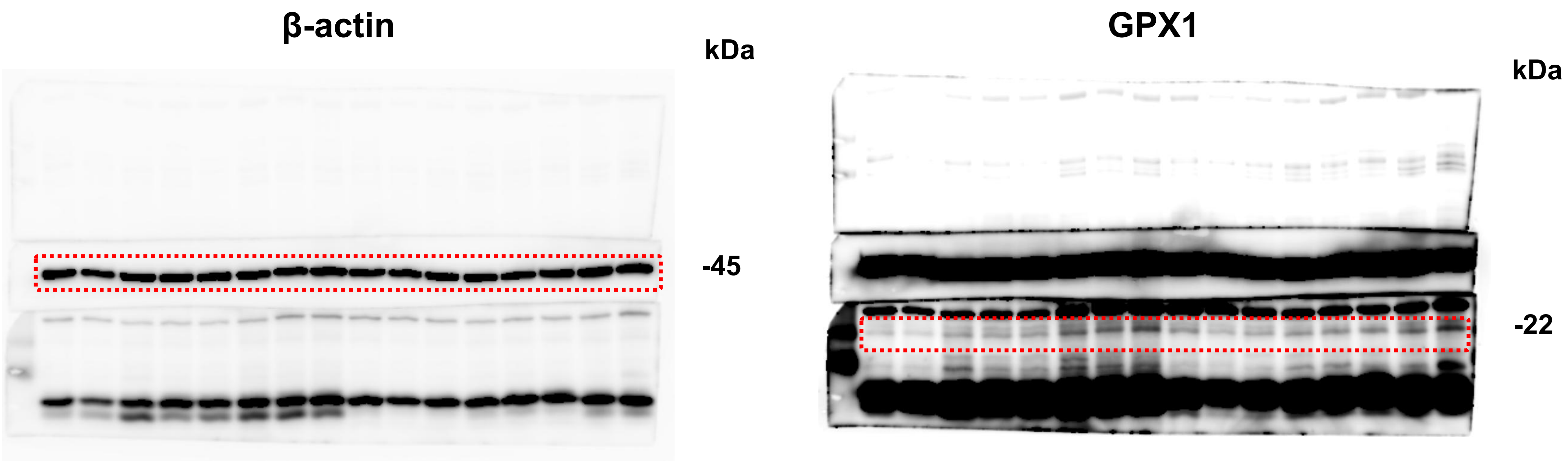

Note: 1. The same blot and different exposure times (short and long exposures shown) were used.
2. Bands used in Figures are indicated by boxes in red outlines.

Fig. 5F (SOD1-Full unedited blot)

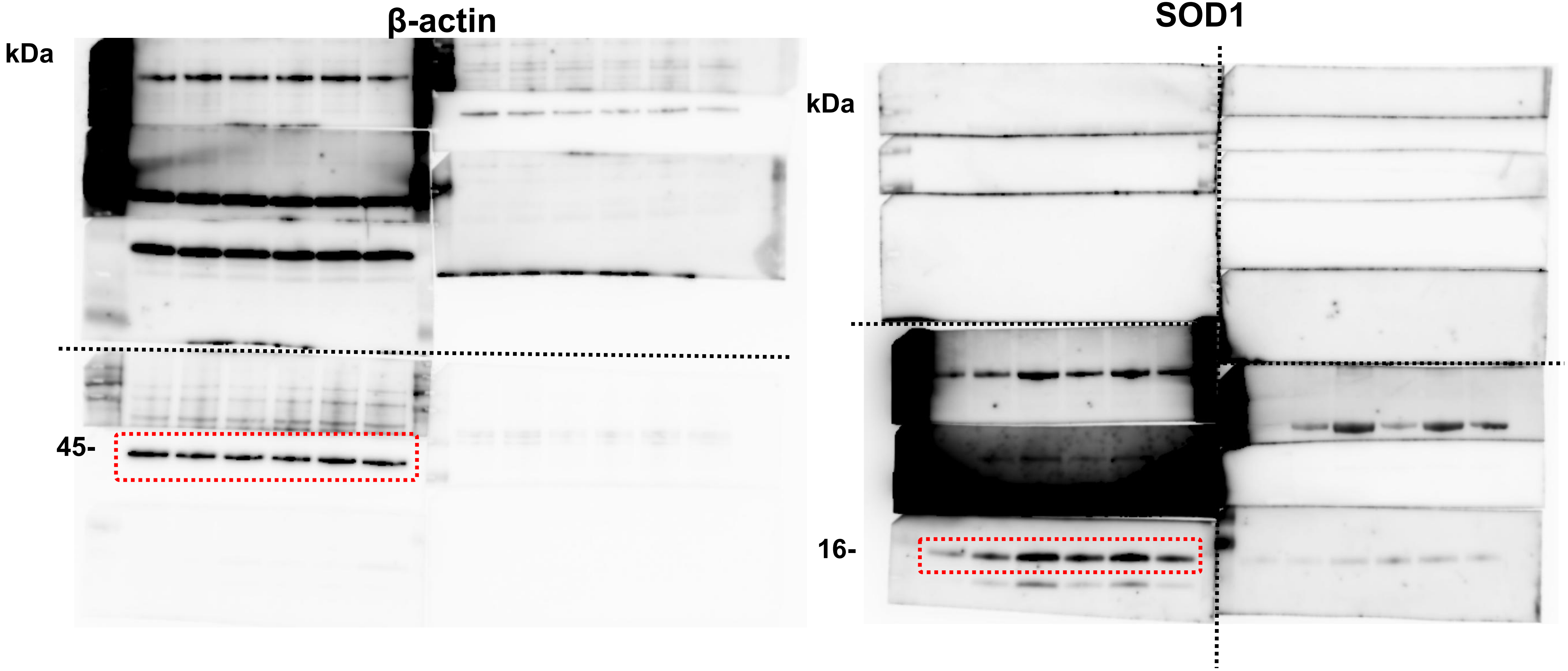

Note: 1. Separate, noncontiguous lanes from the same samples were used for each antibody.
2. Bands used in Figures are indicated by boxes in red outlines.

Fig. 5F (SOD2-Full unedited blot)

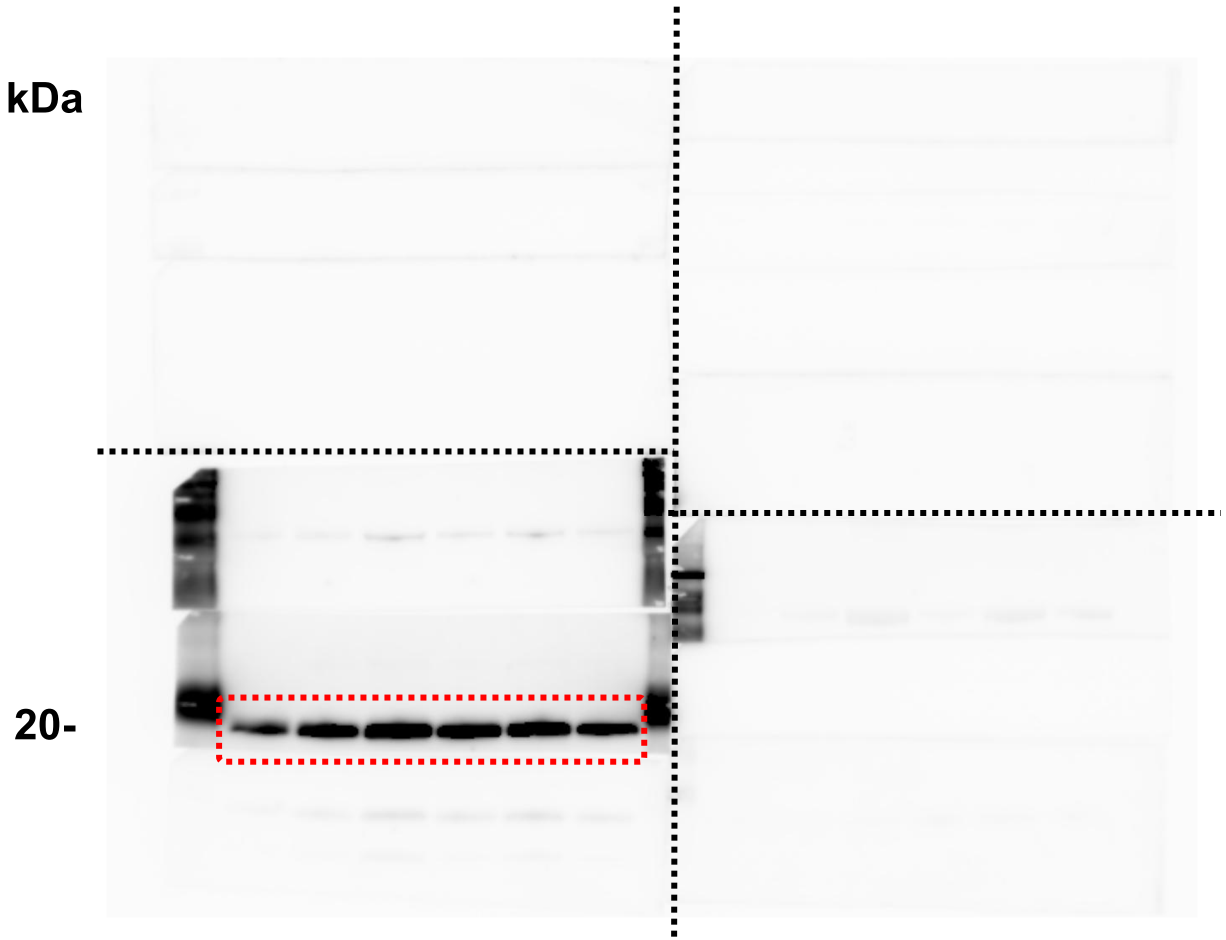

See Fig. 5F (SOD1-Full unedited blot)

Note: 1. Separate, noncontiguous lanes from the same samples were used for each antibody.
2. Bands used in Figures are indicated by boxes in red outlines.

Fig. 5F (CAT-Full unedited blot)

β-actin

See Fig. 5F (SOD1-Full unedited blot)

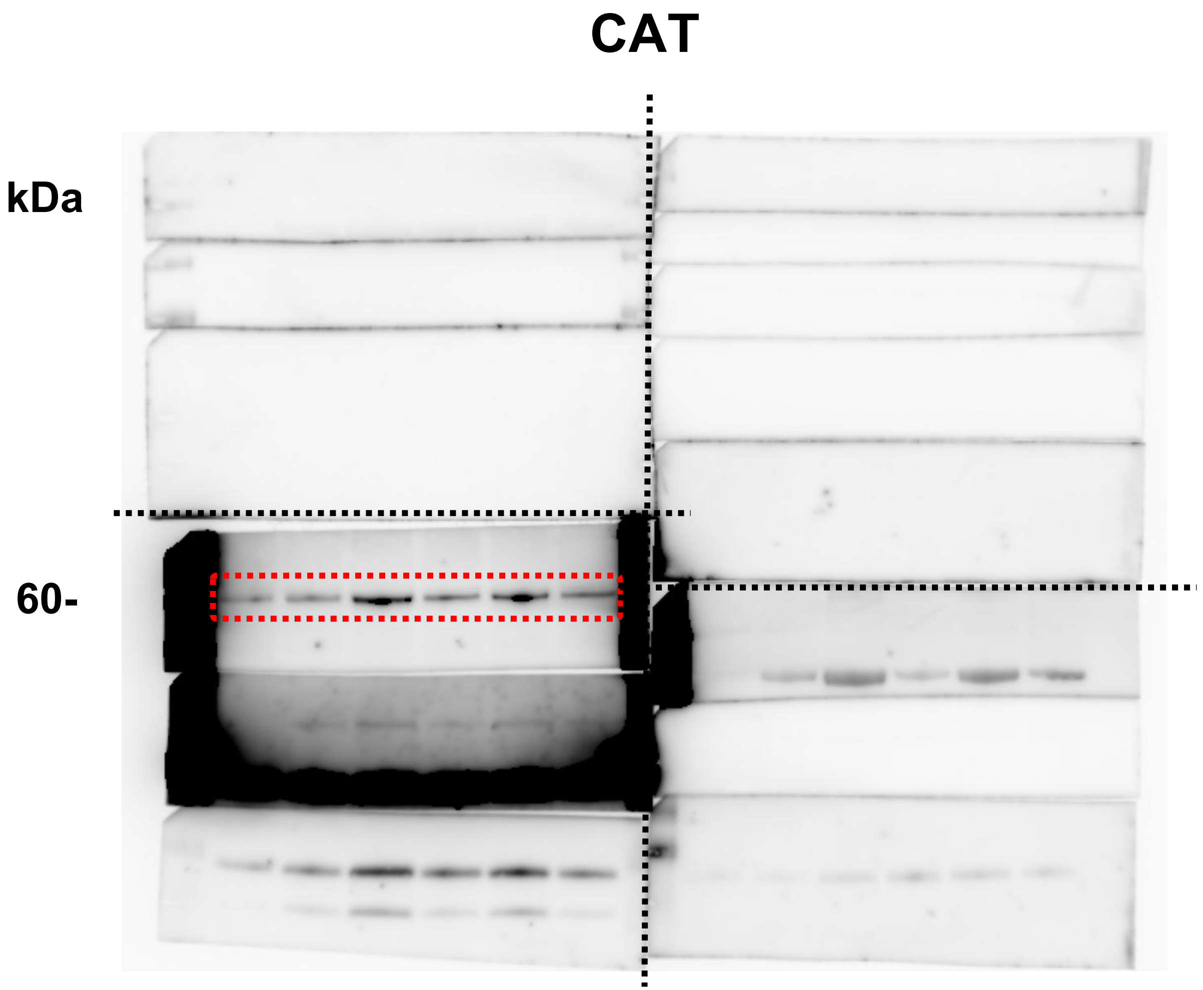

Note: 1. Separate, noncontiguous lanes from the same samples were used for each antibody.
2. Bands used in Figures are indicated by boxes in red outlines.

Fig. 5F (GPX1-Full unedited blot)

β-actin

See Fig. 5F (SOD1-Full unedited blot)

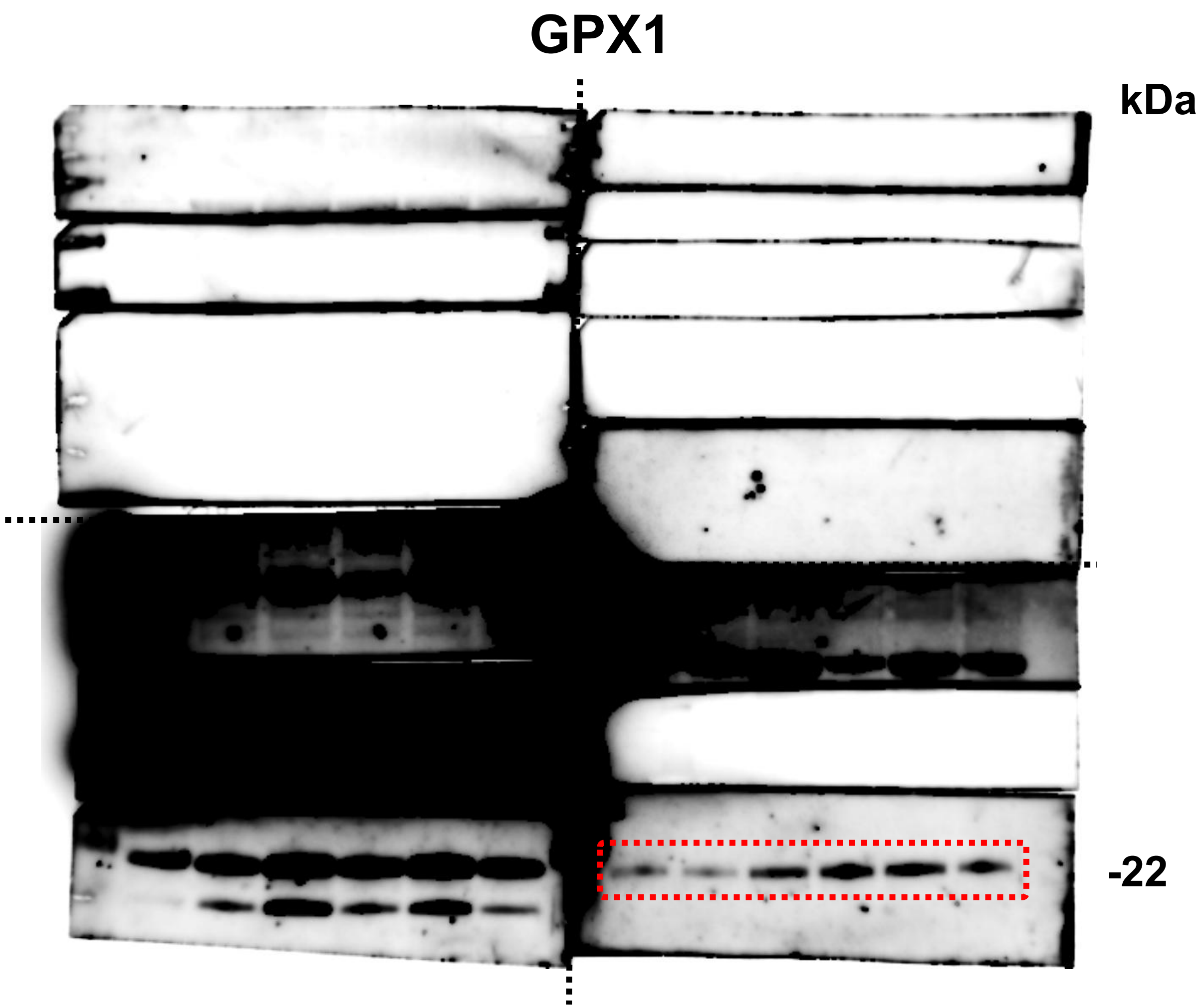

Note: 1. Separate, noncontiguous lanes from the same samples were used for each antibody.
2. Bands used in Figures are indicated by boxes in red outlines.

Fig. 6C (Cleaved-caspase-3/Caspase-3-Full unedited blot)

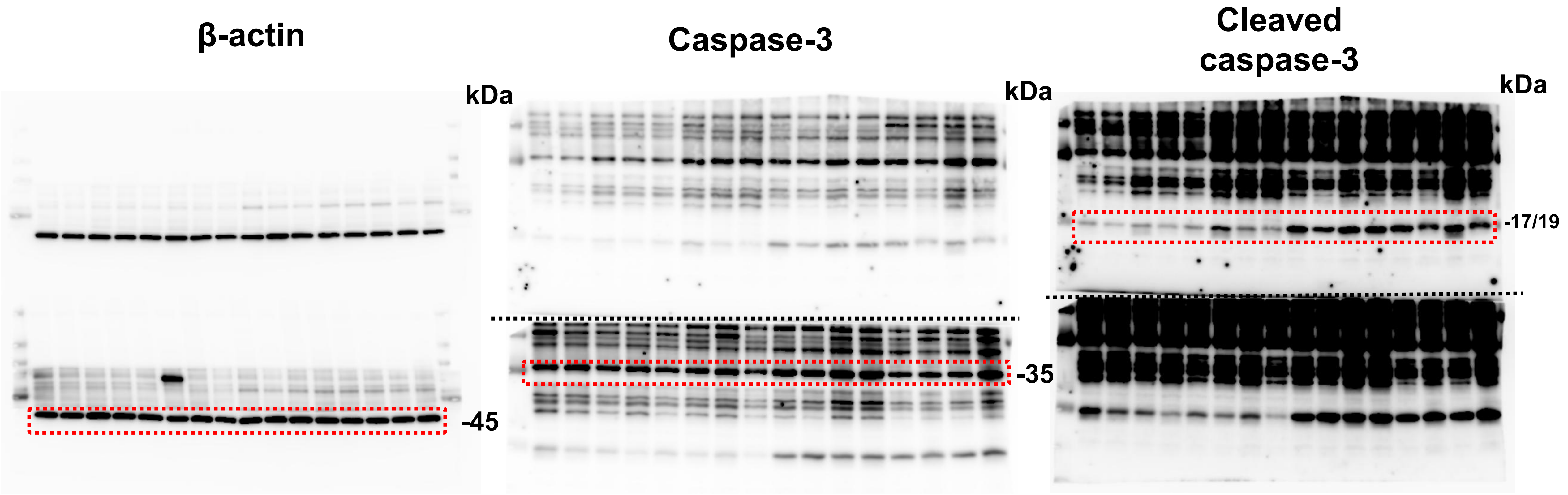

Note: 1. In Cleaved caspase-3 and Caspase-3 expression, the same blot and different exposure times (short and long exposures shown) were used. Between Caspase-3 and β-actin expression, separate, noncontiguous lanes from the same samples were used for each antibody.
2. Bands used in Figures are indicated by boxes in red outlines.

Fig. 6C (BAK-Full unedited blot)

β-actin

See Fig. 1H (CPT1-Full unedited blot)

BAK

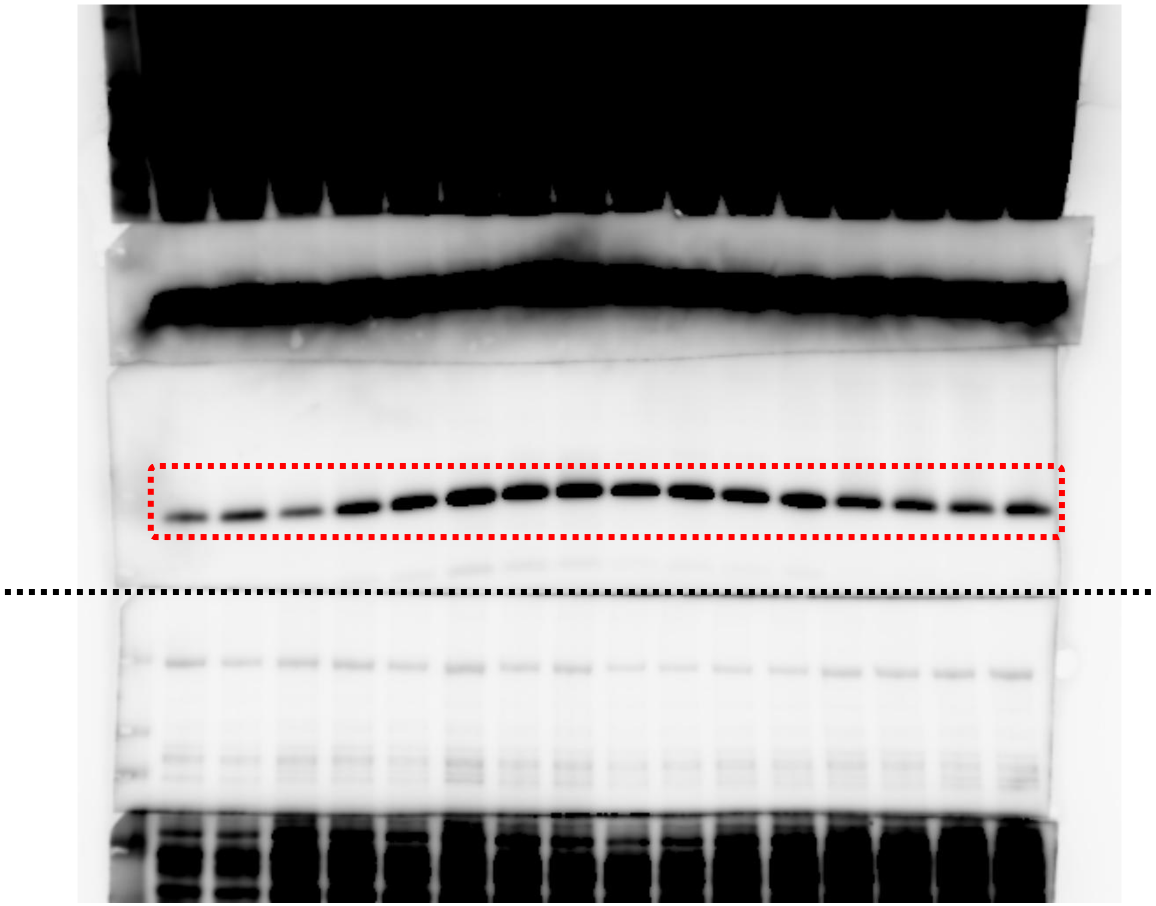

Note: 1. The same blot and different exposure times (short and long exposures shown) were used.
β-actin loading control for BAK was obtained from the same gel with CPT1.
2. Bands used in Figures are indicated by boxes in red outlines.

Fig. 6C (BAX-Full unedited blot)

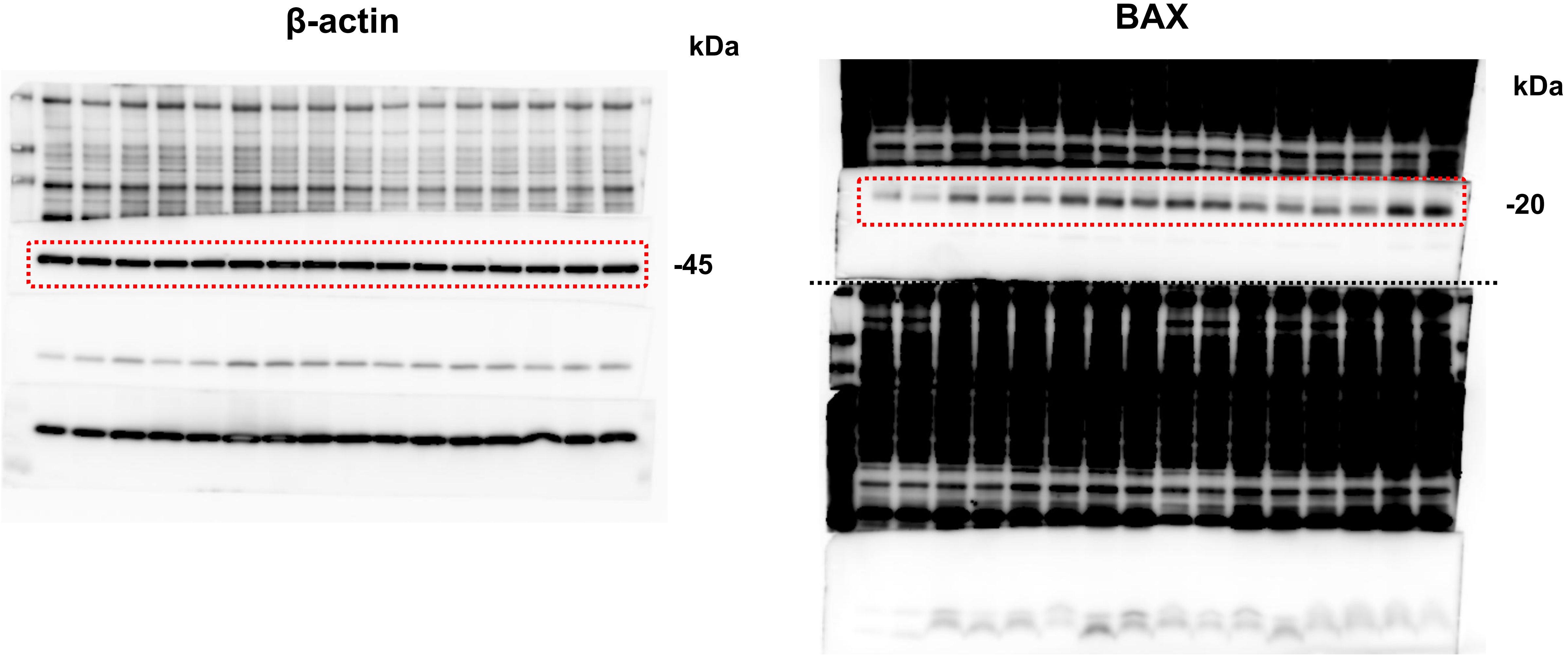

Note: 1. Separate, noncontiguous lanes from the same samples were used for each antibody.
2. Bands used in Figures are indicated by boxes in red outlines.

Fig. 6C (Cleaved-caspase-8/Caspase-8-Full unedited blot)

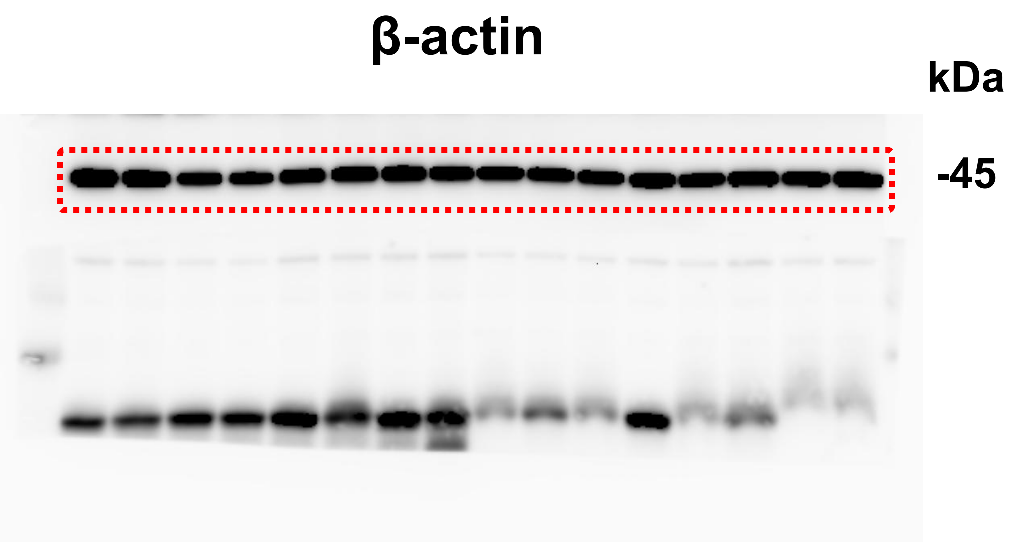

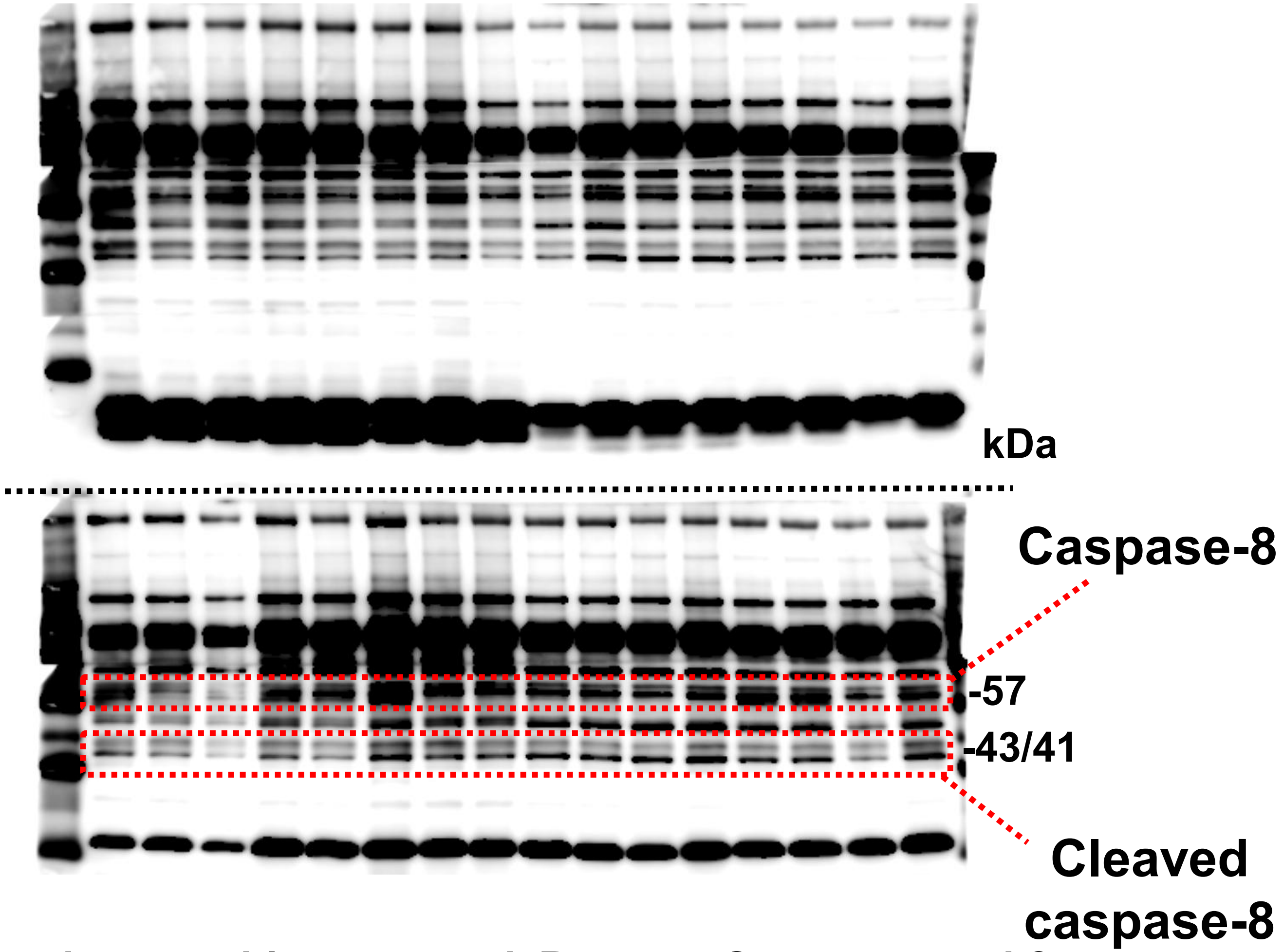

Note: 1. In Cleaved-caspase-8 and Caspase-8 expression, the same blot was used. Between Caspase-e and β-actin expression, separate, noncontiguous lanes from the same samples were used for each antibody.
2. Bands used in Figures are indicated by boxes in red outlines.

Fig. 6E (BAK-Full unedited blot)

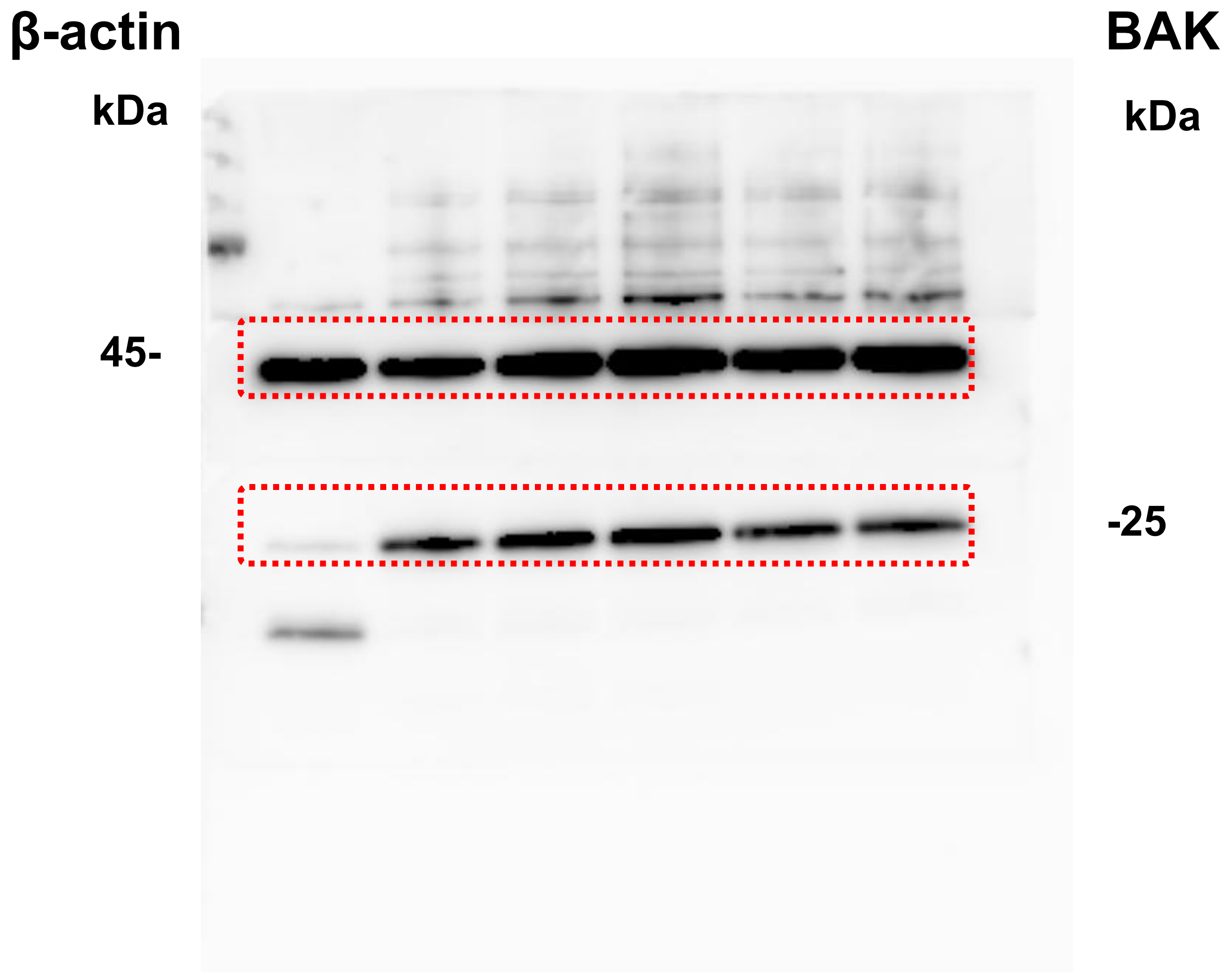

Note: 1. The same blot was used.
2. Bands used in Figures are indicated by boxes in red outlines.

Fig. 6E (Cleaved-caspase-8/Caspase-8-Full unedited blot)

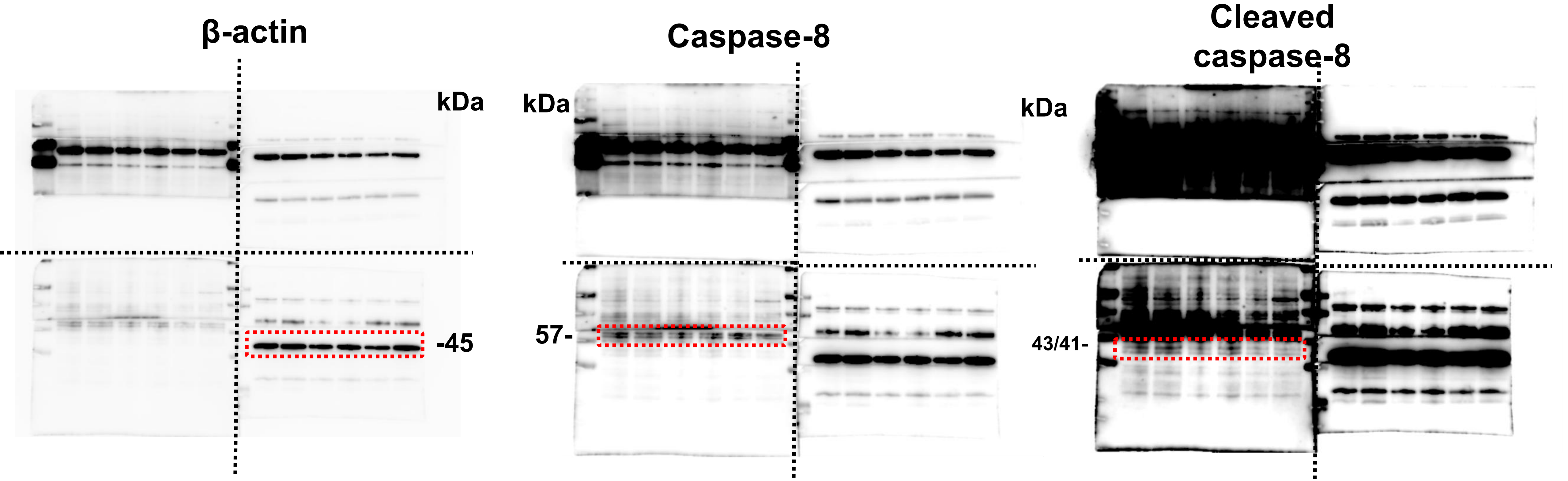

Note: 1. In Cleaved caspase-8 and Caspase-8 expression, the same blot and different exposure times (short and long exposures shown) were used. Between Caspase-8 and β-actin expression, separate, noncontiguous lanes from the same samples were used for each antibody.
2. Bands used in Figures are indicated by boxes in red outlines.

Fig. 7A (p-STAT3-STAT3-Full unedited blot)

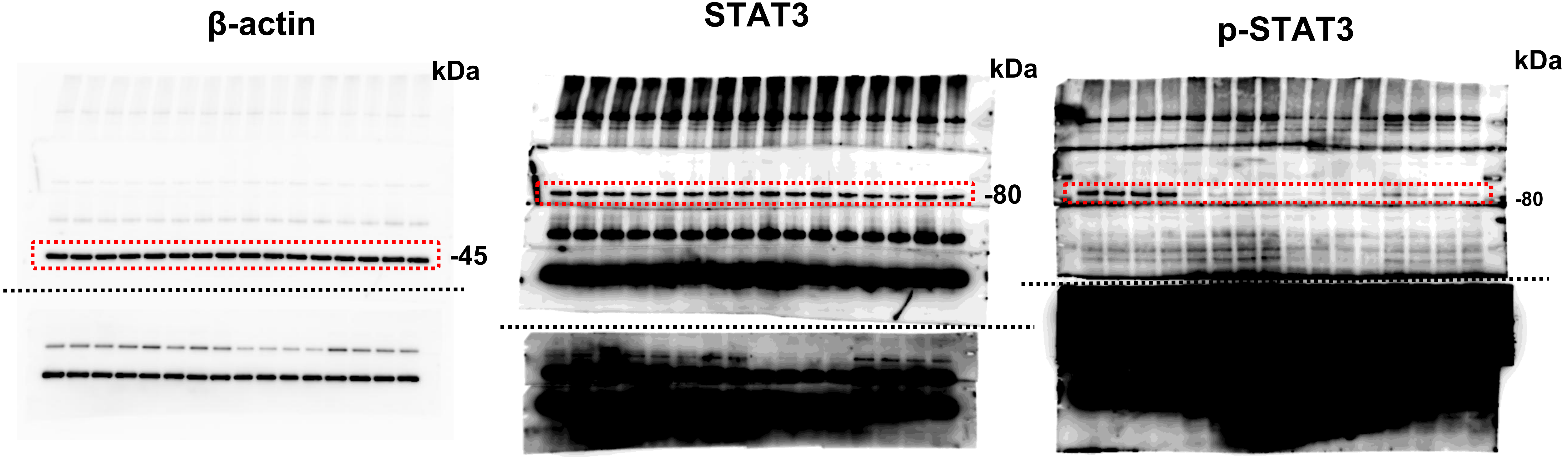

Note: 1. In STAT3 and β-acti expression, the same blot and different exposure times (short and long exposures shown) were used. Between p-STAT3 and STAT3 expression, separate, noncontiguous lanes from the same samples were used for each antibody.
2. Bands used in Figures are indicated by boxes in red outlines.

Fig. 7A (p-AKT-AKT-Full unedited blot)

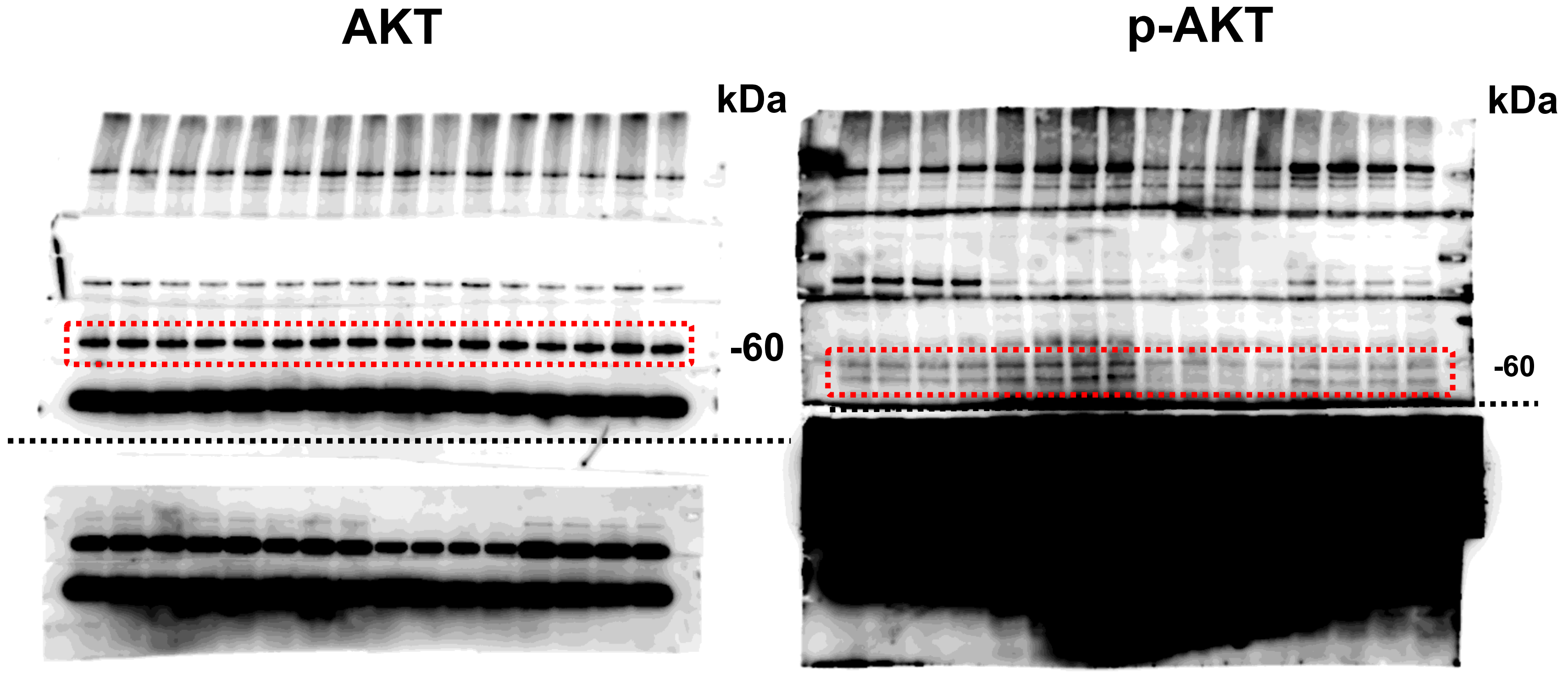

See Fig. 7A (p-STAT3-STAT3-Full unedited blot)

Note: 1. In AKT and β-acti expression, the same blot and different exposure times (short and long exposures shown) were used. Between p-AKT and AKT expression, separate, noncontiguous lanes from the same samples were used for each antibody.
2. Bands used in Figures are indicated by boxes in red outlines.

Fig. 7A (p-mTOR-,TOR-Full unedited blot)

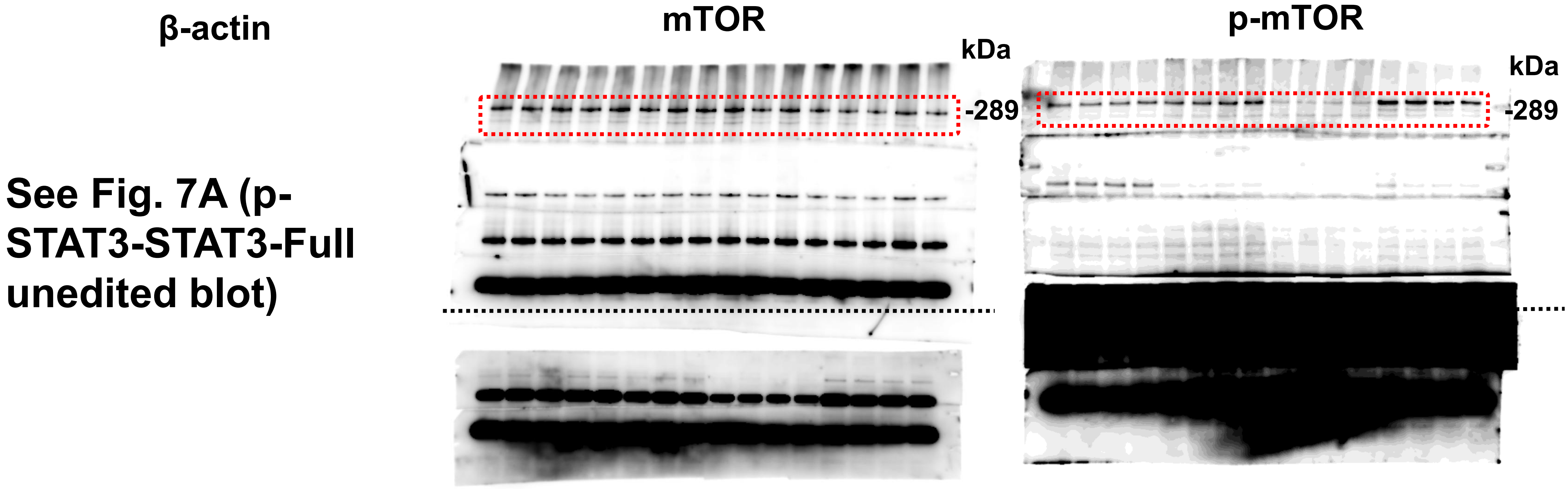

Note: 1. In mTOR and β-acti expression, the same blot and different exposure times (short and long exposures shown) were used. Between p-mTOR and mTOR expression, separate, noncontiguous lanes from the same samples were used for each antibody.
2. Bands used in Figures are indicated by boxes in red outlines.

Fig. 7B (p-STAT3-STAT3-Full unedited blot)

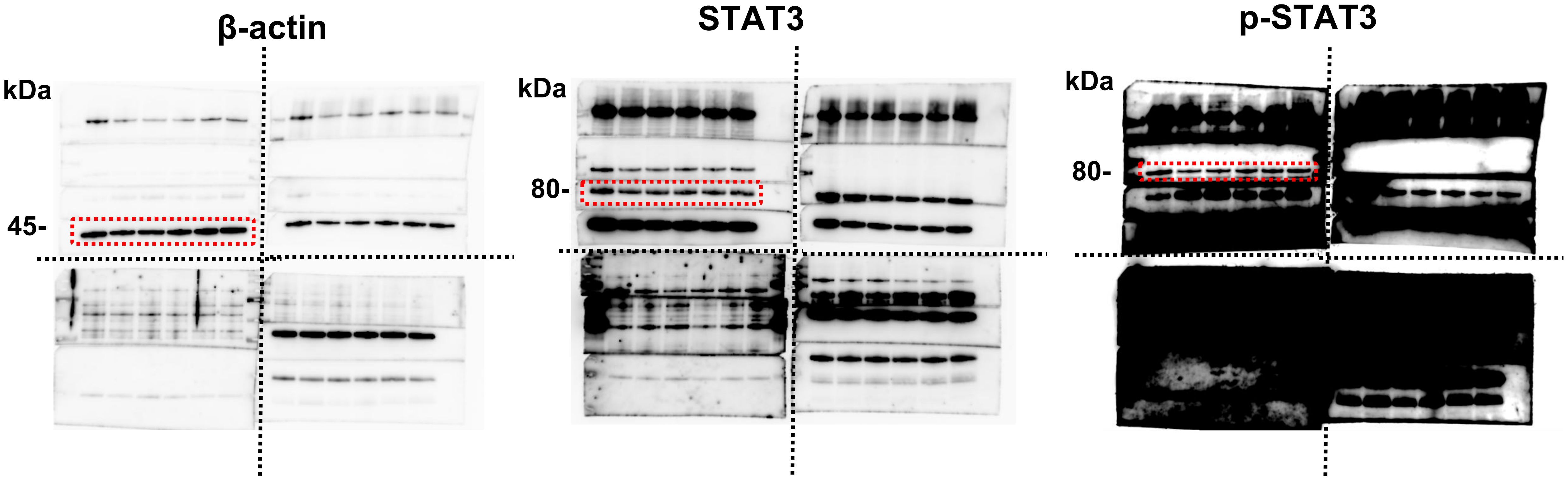

Note: 1. In STAT3 and β-actin expression, the same blot and different exposure times (short and long exposures shown) were used. Between p-STAT3 and STAT3 expression, separate, noncontiguous lanes from the same samples were used for each antibody.
2. Bands used in Figures are indicated by boxes in red outlines.

Fig. 7B (p-AKT-AKT-Full unedited blot)

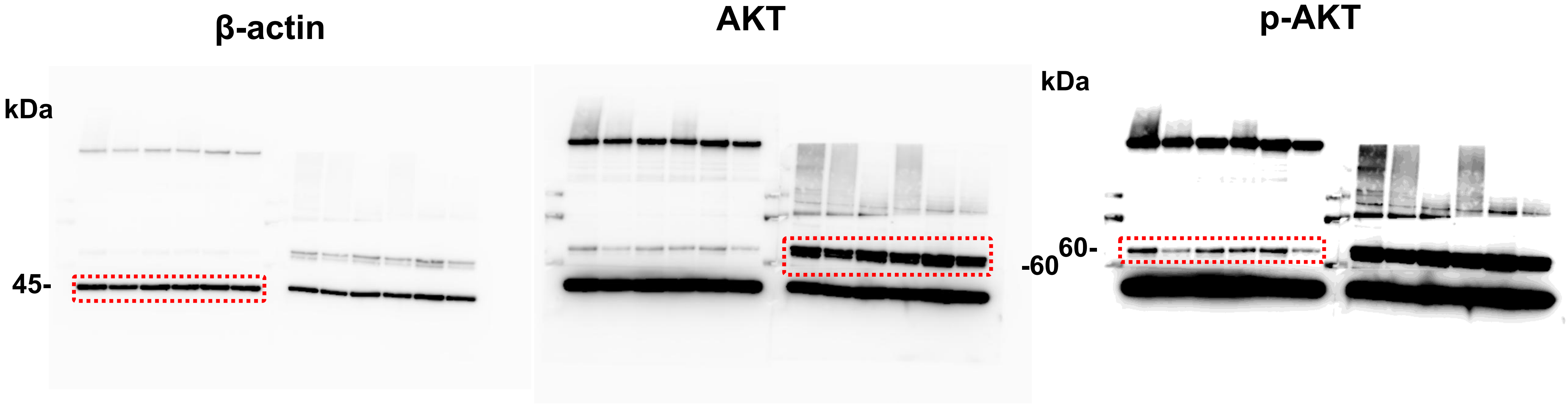

Note: 1. The same blot and different exposure times (short and long exposures shown) were used. 2. Bands used in Figures are indicated by boxes in red outlines.

Fig. 7B (p-mTOR-mTOR-Full unedited blot)

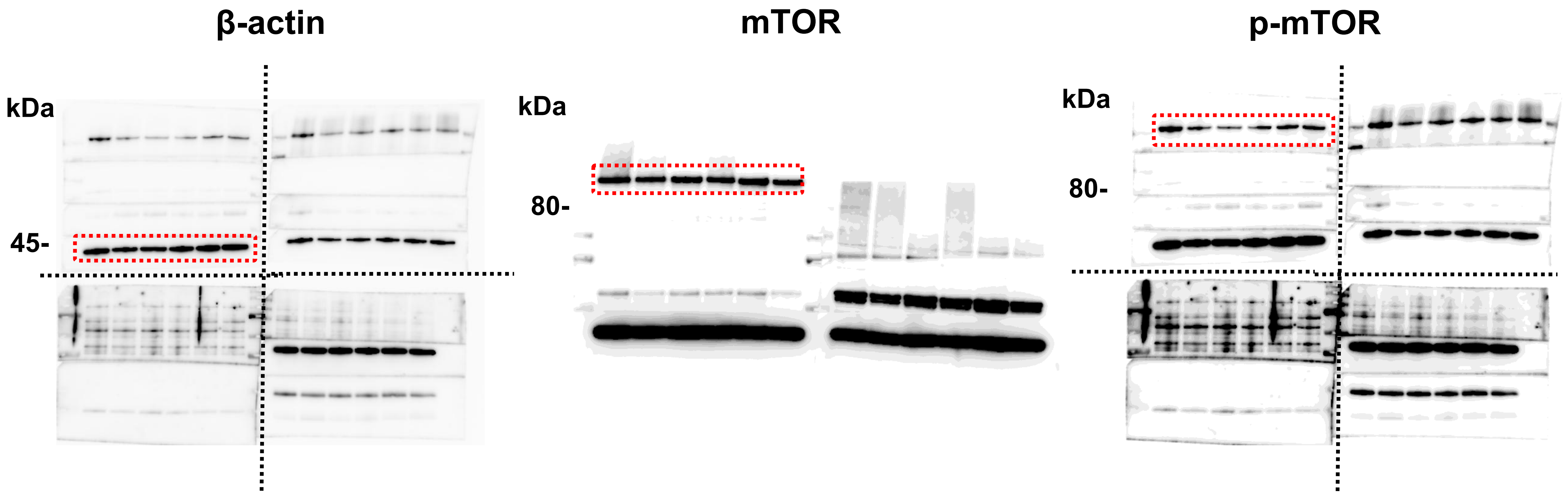

Note: 1. In p-mTOR and β-actin expression, the same blot and different exposure times (short and long exposures shown) were used. Between p-mTOR and mTOR expression, separate, noncontiguous lanes from the same samples were used for each antibody.
2. Bands used in Figures are indicated by boxes in red outlines.

Fig. 7C (p-STAT3-STAT3-Full unedited blot)

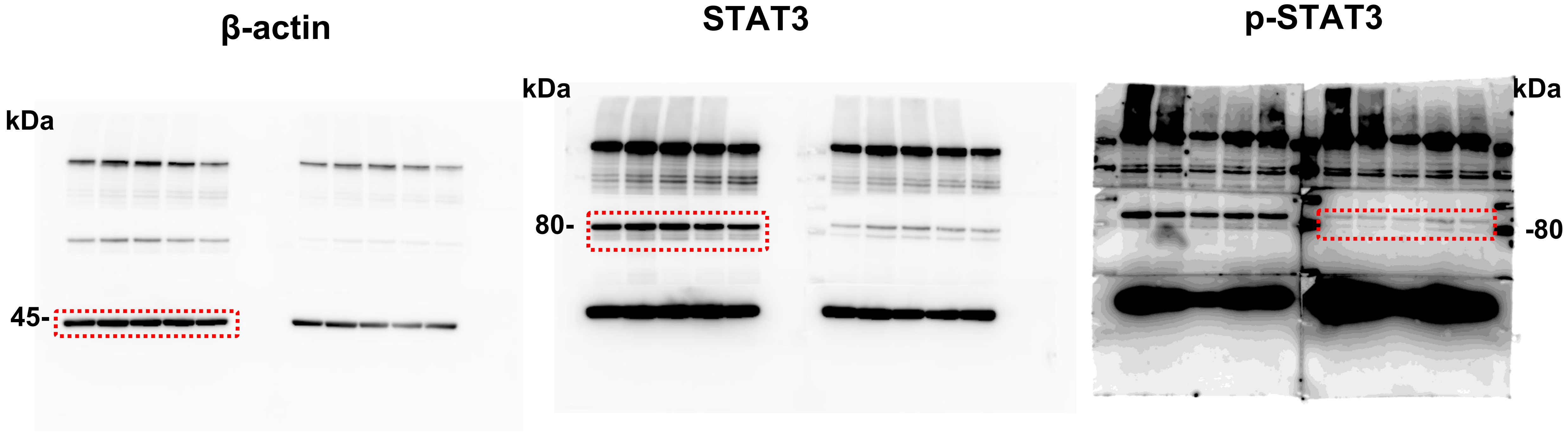

Note: 1. In STAT3 and β-actin expression, the same blot and different exposure times (short and long exposures shown) were used. Between p-STAT3 and STAT3 expression, separate, noncontiguous lanes from the same samples were used for each antibody.

2. Bands used in Figures are indicated by boxes in red outlines.

Fig. 7C (p-AKT-AKT-Full unedited blot)

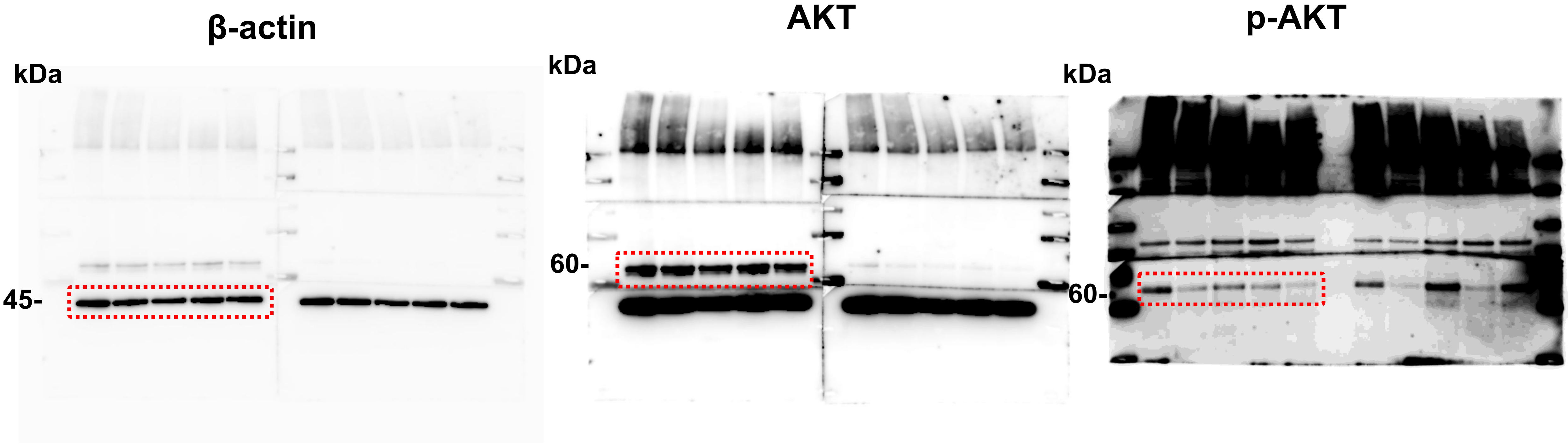

Note: 1. In AKT and β-actin expression, the same blot and different exposure times (short and long exposures shown) were used. Between p-AKT and AKT expression, separate, noncontiguous lanes from the same samples were used for each antibody.
2. Bands used in Figures are indicated by boxes in red outlines.

Fig. 7C (p-mTOR-mTOR-Full unedited blot)

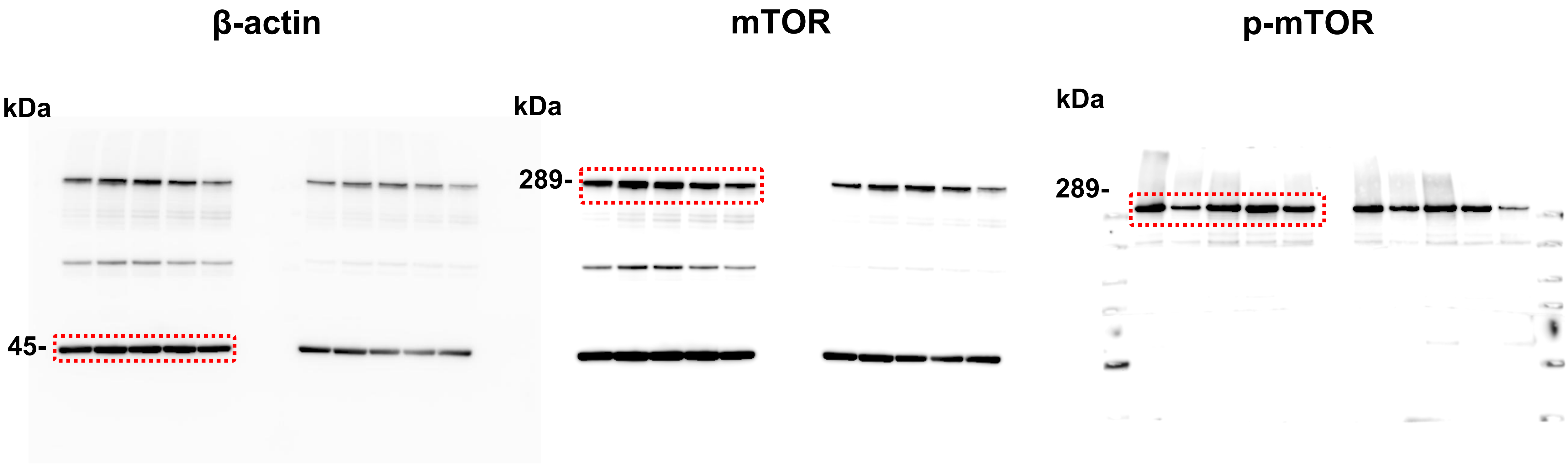

Note: 1. In p-mTOR and β-actin expression, the same blot and different exposure times (short and long exposures shown) were used. Between p-mTOR and mTOR expression, separate, noncontiguous lanes from the same samples were used for each antibody.
2. Bands used in Figures are indicated by boxes in red outlines.

Fig. 7D (p-AKT-AKT-Full unedited blot)

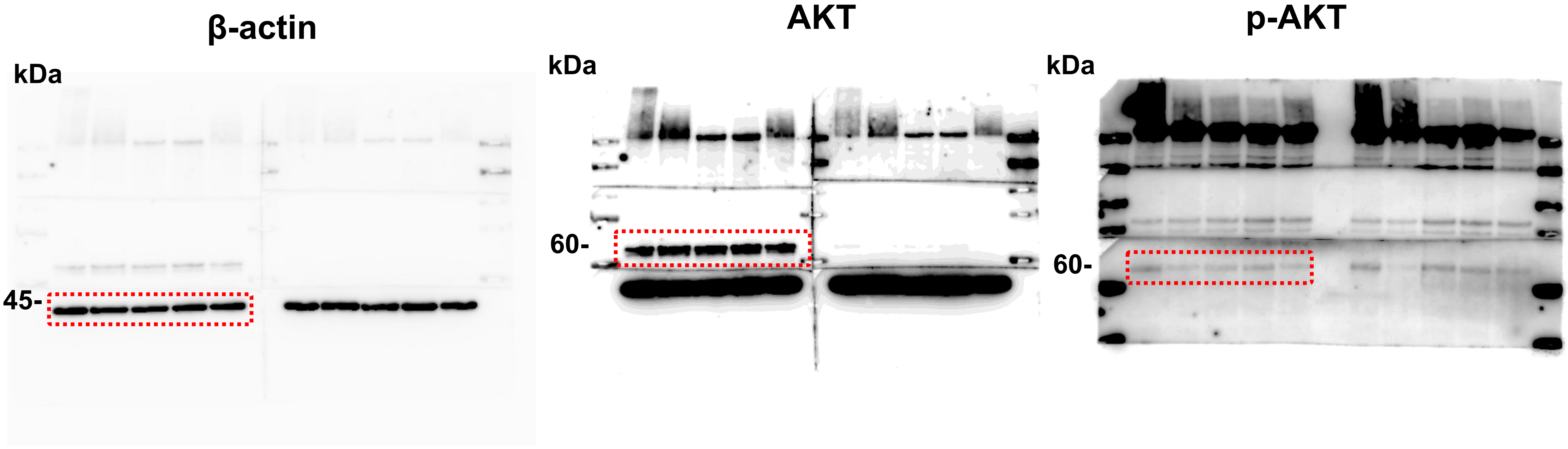

Note: 1. In AKT and β-actin expression, the same blot and different exposure times (short and long exposures shown) were used. Between p-AKT and AKT expression, separate, noncontiguous lanes from the same samples were used for each antibody.
2. Bands used in Figures are indicated by boxes in red outlines.

Fig. 7D (p-mTOR-mTOR-Full unedited blot)

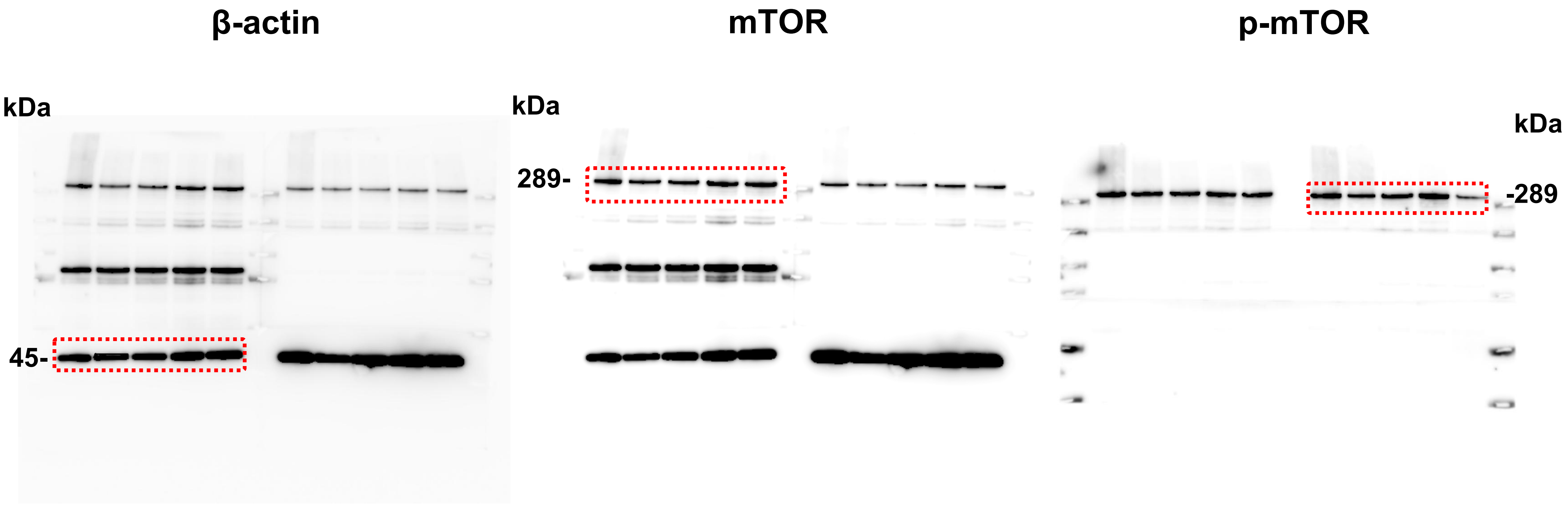

Note: 1. In p-mTOR and β-actin expression, the same blot and different exposure times (short and long exposures shown) were used. Between p-mTOR and mTOR expression, separate, noncontiguous lanes from the same samples were used for each antibody.
2. Bands used in Figures are indicated by boxes in red outlines.

Fig. 7E (p-STAT3-STAT3-Full unedited blot)

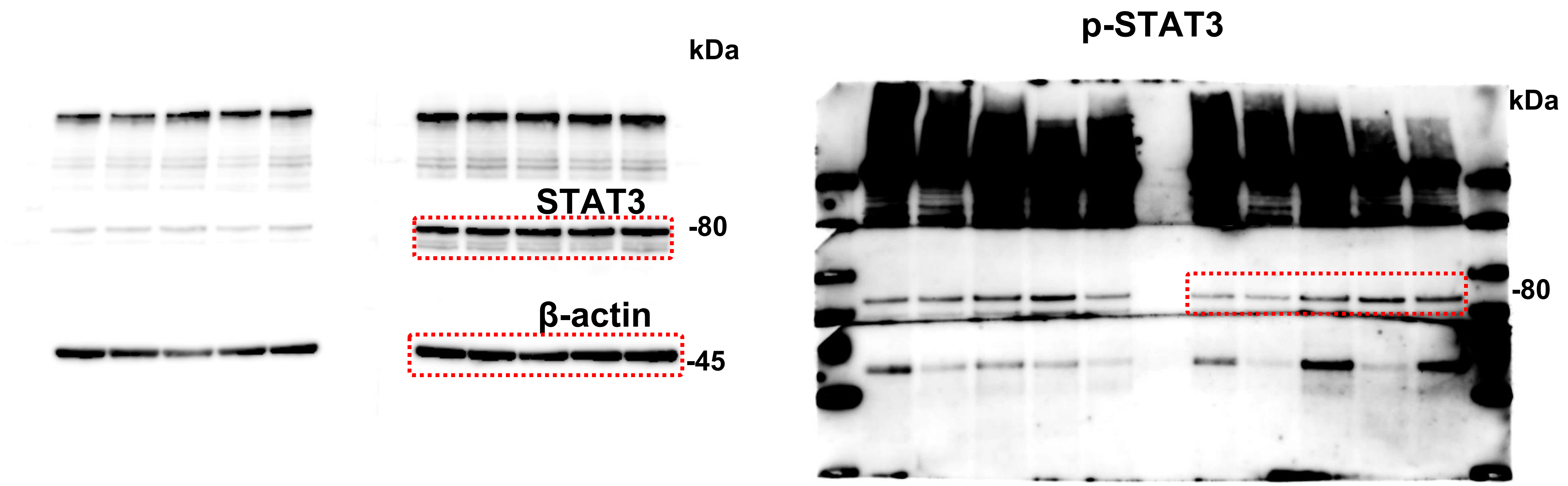

Note: 1. In STAT3 and β-actin expression, the same blot was used. Between p-STAT3 and STAT3 expression, separate, noncontiguous lanes from the same samples were used for each antibody.
2. Bands used in Figures are indicated by boxes in red outlines.

Fig. 7E (p-mTOR-mTOR-Full unedited blot)

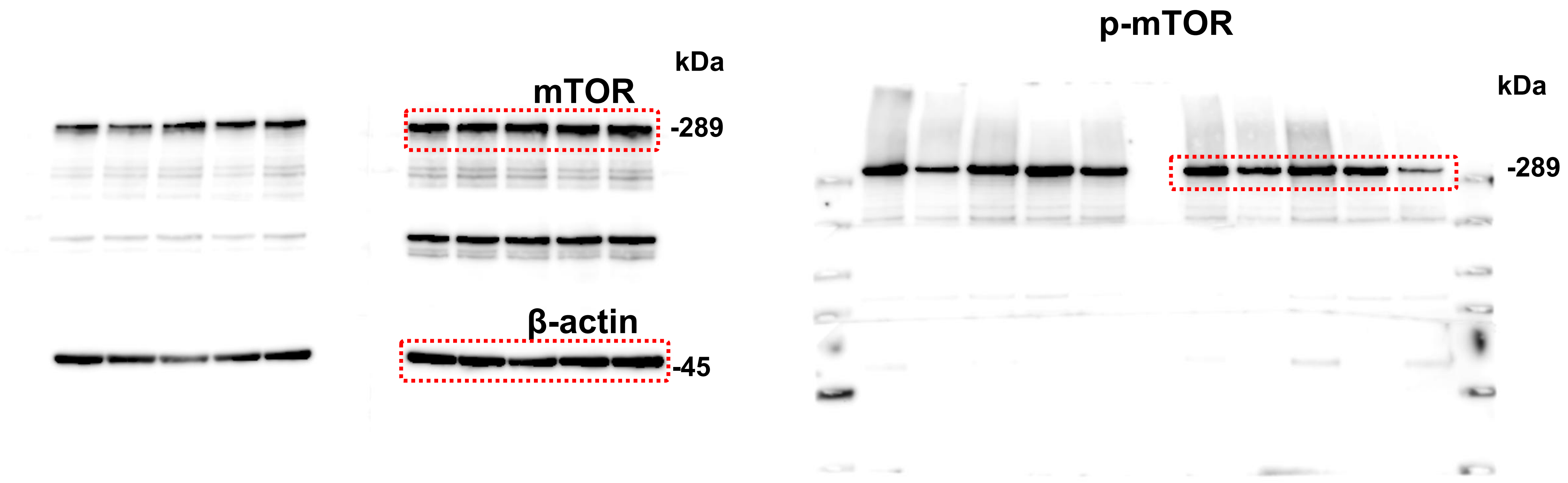

Note: 1. In p-mTOR and β-actin expression, the same blot was used. Between p-mTOR and mTOR expression, separate, noncontiguous lanes from the same samples were used for each antibody.
2. Bands used in Figures are indicated by boxes in red outlines.

Fig. 7F (p-STAT3-STAT3-Full unedited blot)

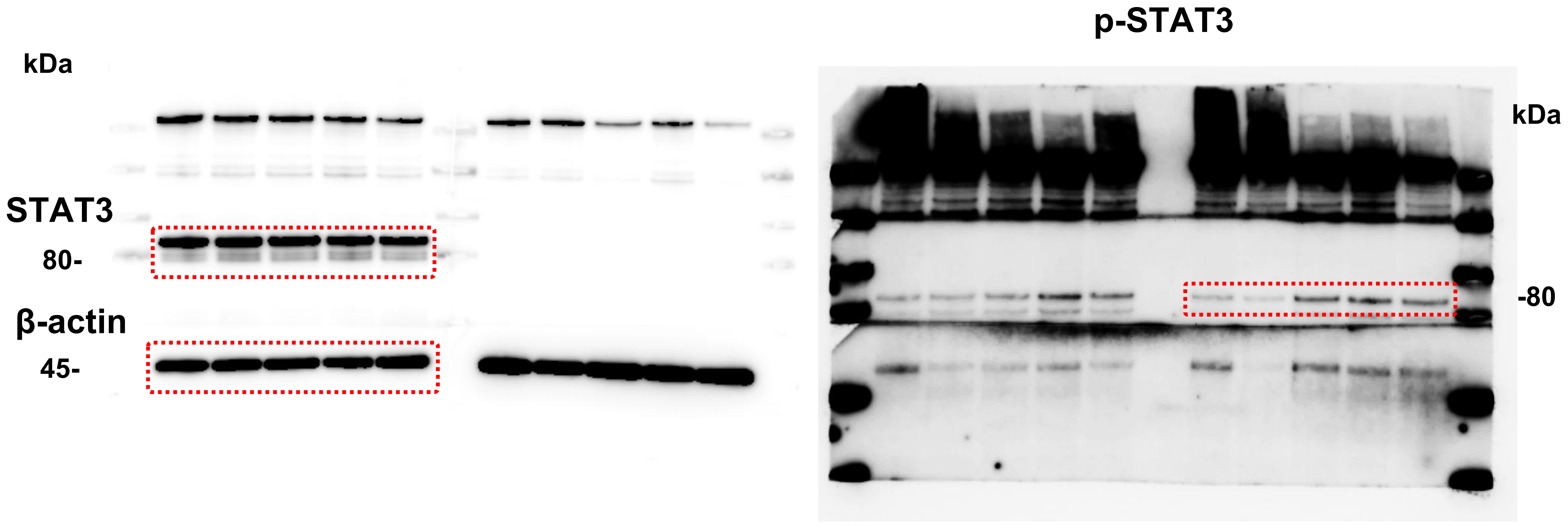

Note: 1. In STAT3 and β-actin expression, the same blot and different exposure times (short and long exposures shown) were used. Between p-STAT3 and STAT3 expression, separate, noncontiguous lanes from the same samples were used for each antibody.
2. Bands used in Figures are indicated by boxes in red outlines.

Fig. 7F (p-AKT-AKT-Full unedited blot)

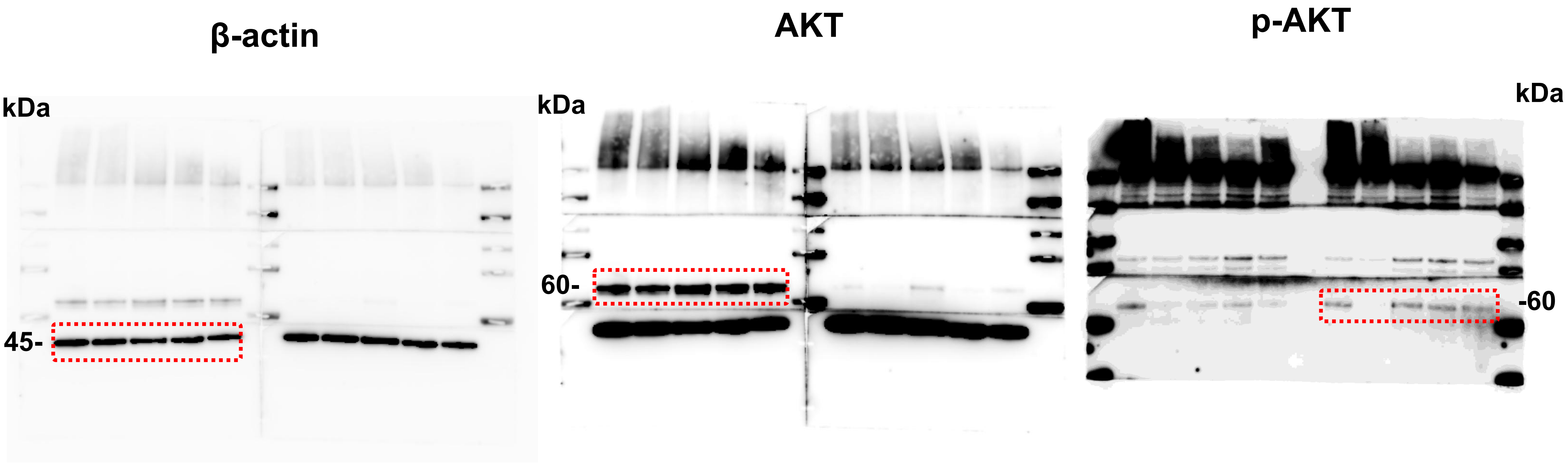

Note: 1. In AKT and β-actin expression, the same blot and different exposure times (short and long exposures shown) were used. Between p-AKT and AKT expression, separate, noncontiguous lanes from the same samples were used for each antibody.
2. Bands used in Figures are indicated by boxes in red outlines.

Fig. S2C (FAS-Full unedited blot)

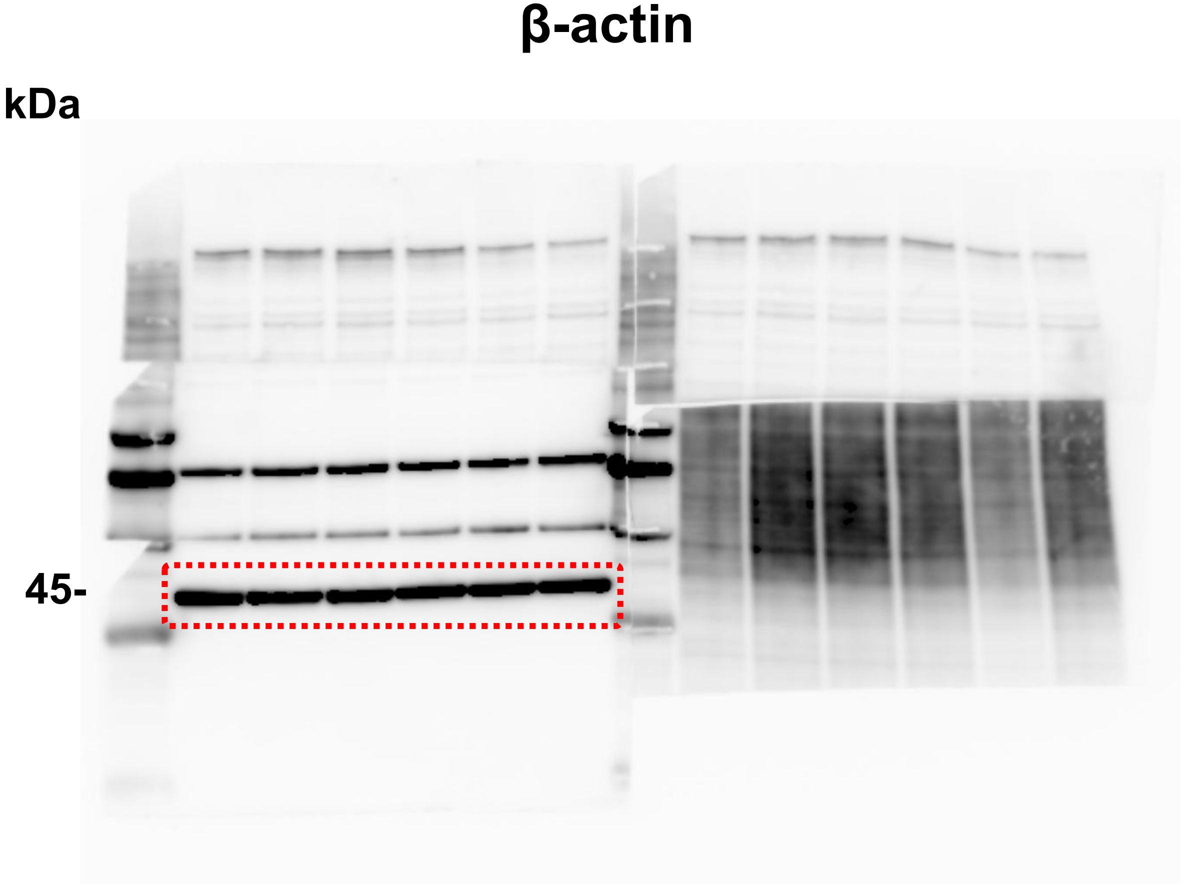

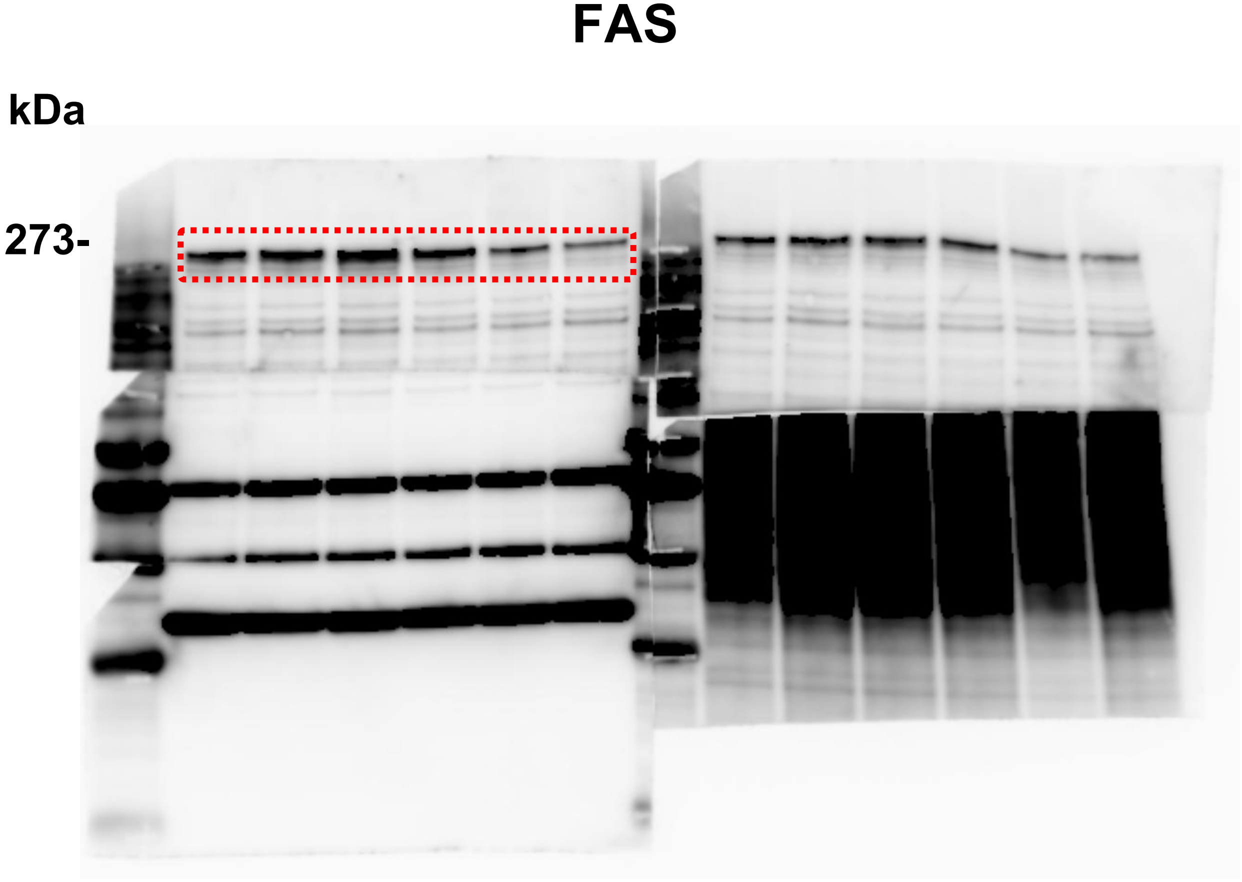

Note: 1. The same blot and different exposure times (short and long exposures shown) were used.
2. Bands used in Figures are indicated by boxes in red outlines.

Fig. S2C (CPT1-Full unedited blot)

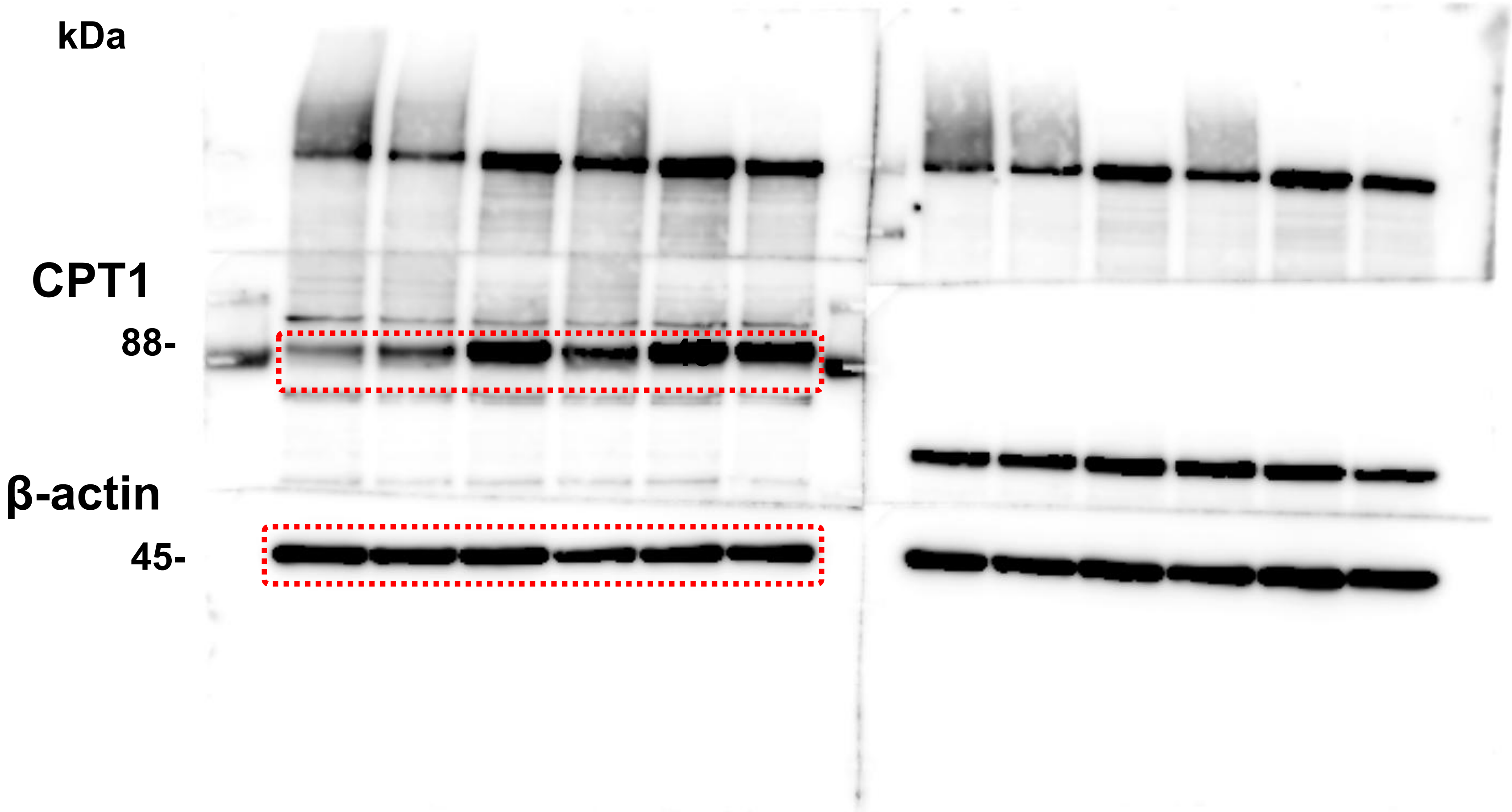

Note: 1. The same blot was used.
2. Bands used in Figures are indicated by boxes in red outlines.

Fig. S2C (CPT2-Full unedited blot)

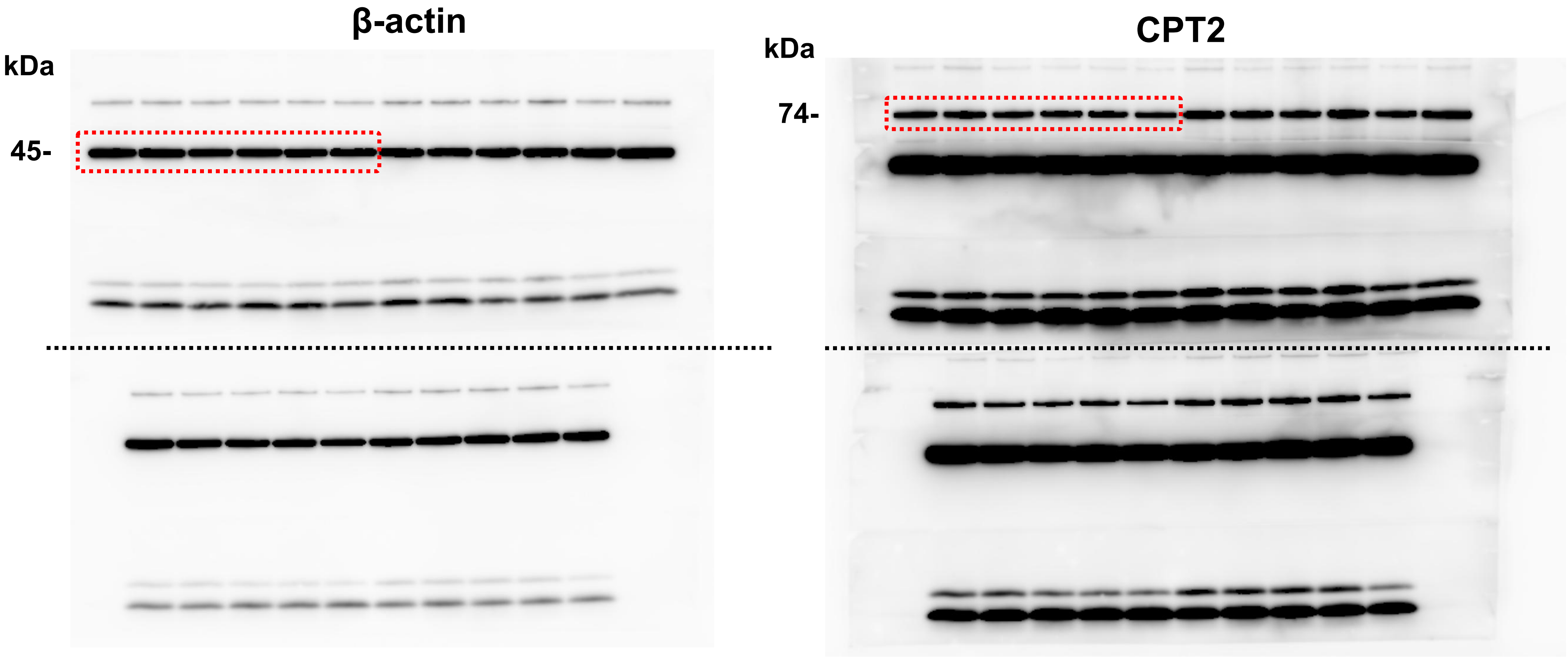

Note: 1. The same blot and different exposure times (short and long exposures shown) were used.
2. Bands used in Figures are indicated by boxes in red outlines.

Fig. S2C (PPARα-Full unedited blot)

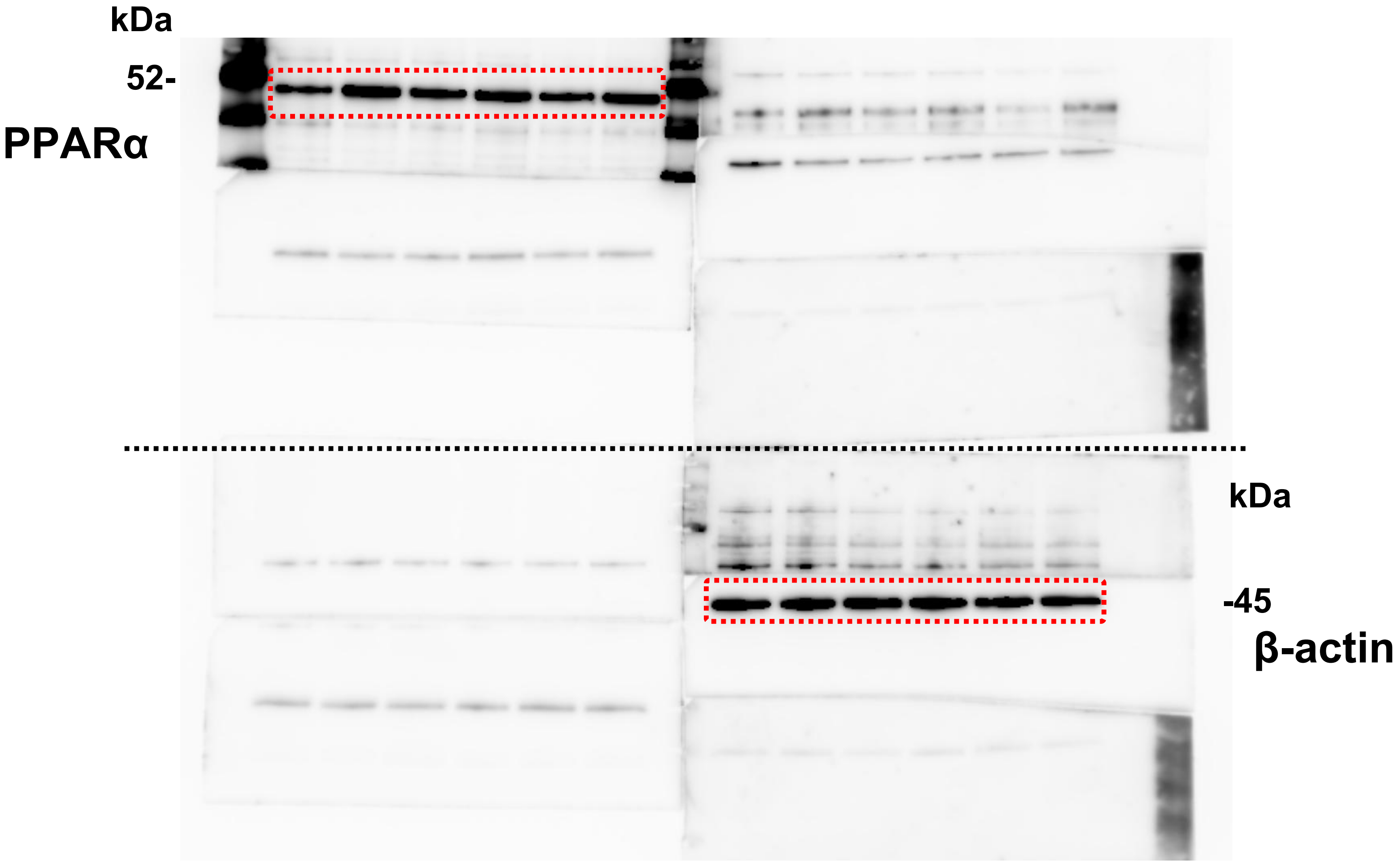

Note: 1. The same blot was used.
2. Bands used in Figures are indicated by boxes in red outlines.

Fig. S4B (IL-10Rα Full unedited blot)

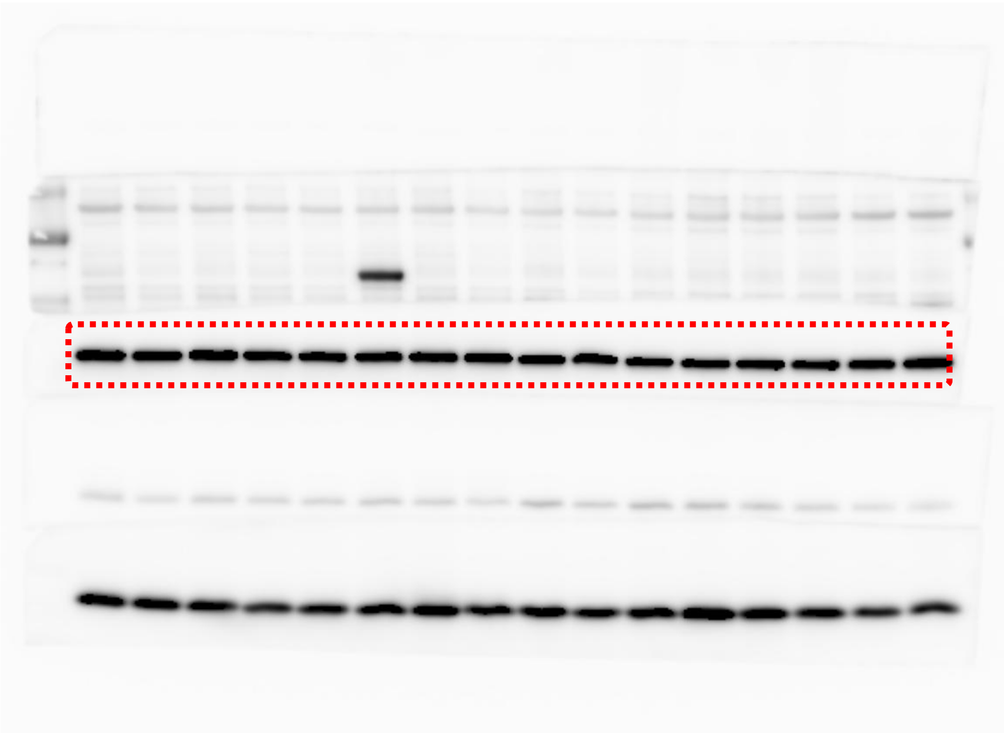

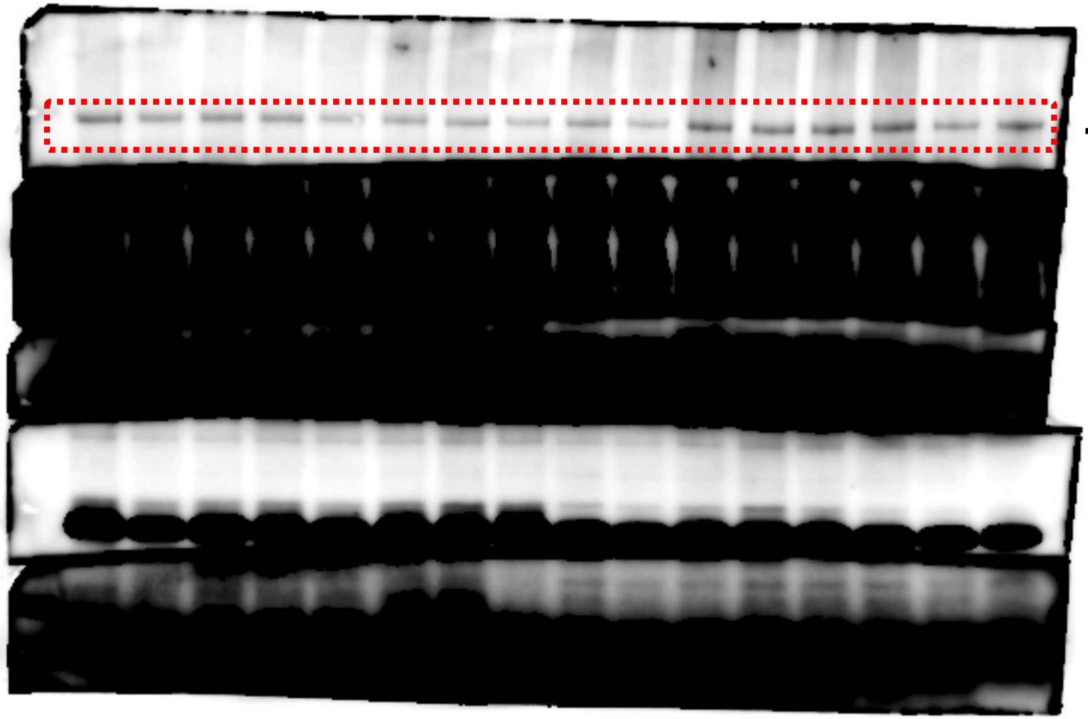

Note: 1. The same blot and different exposure times (short and long exposures shown) were used.
2. Bands used in Figures are indicated by boxes in red outlines.

Fig. S4D (FAS-Full unedited blot)

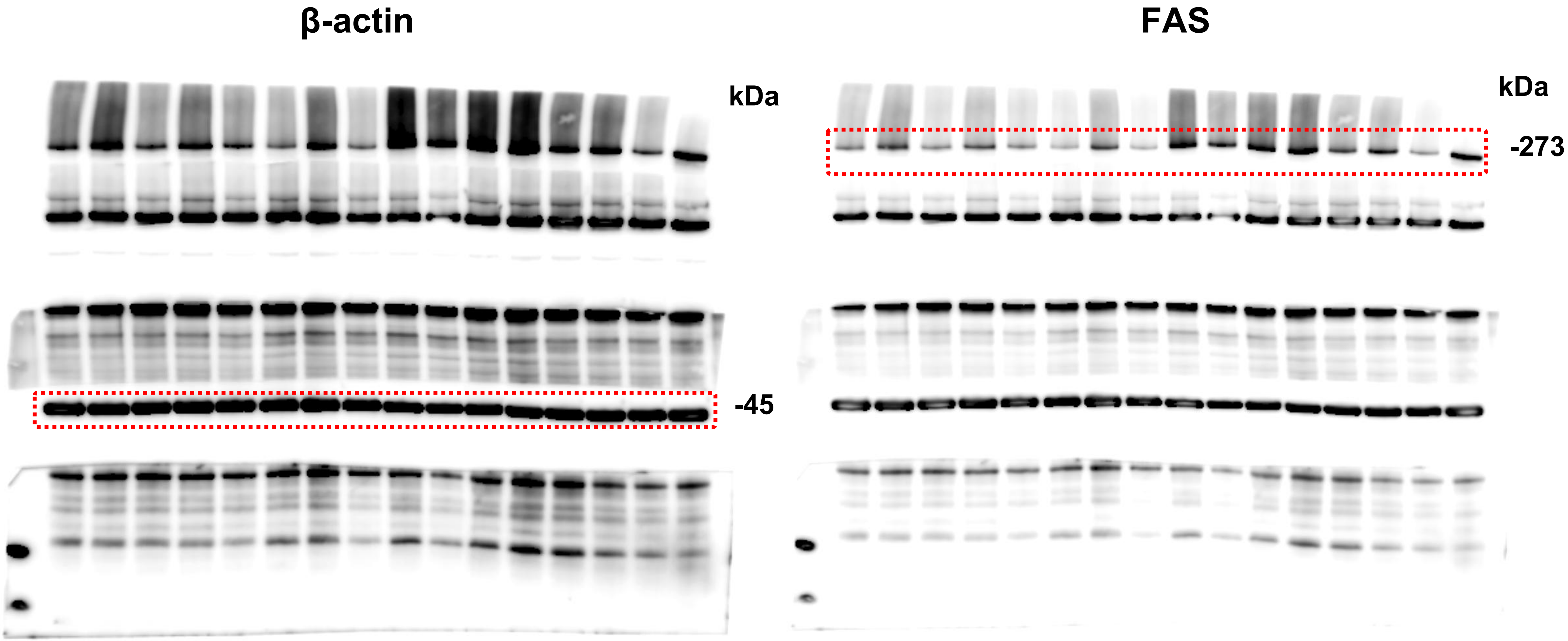

Note: 1. The same blot and different exposure times (short and long exposures shown) were used..
2. Bands used in Figures are indicated by boxes in red outlines.

Fig. S4D (CPT1-Full unedited blot)

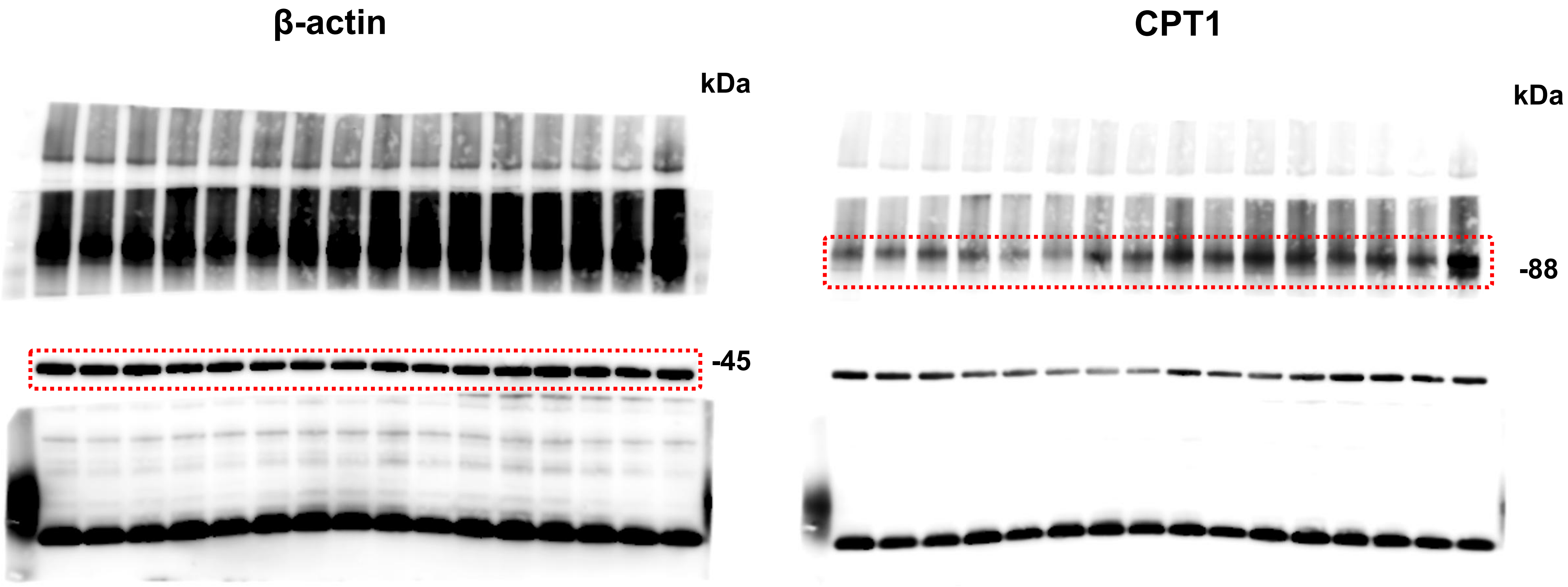

Note: 1. The same blot and different exposure times (short and long exposures shown) were used.
2. Bands used in Figures are indicated by boxes in red outlines.

Fig. S4D (CPT2-Full unedited blot)

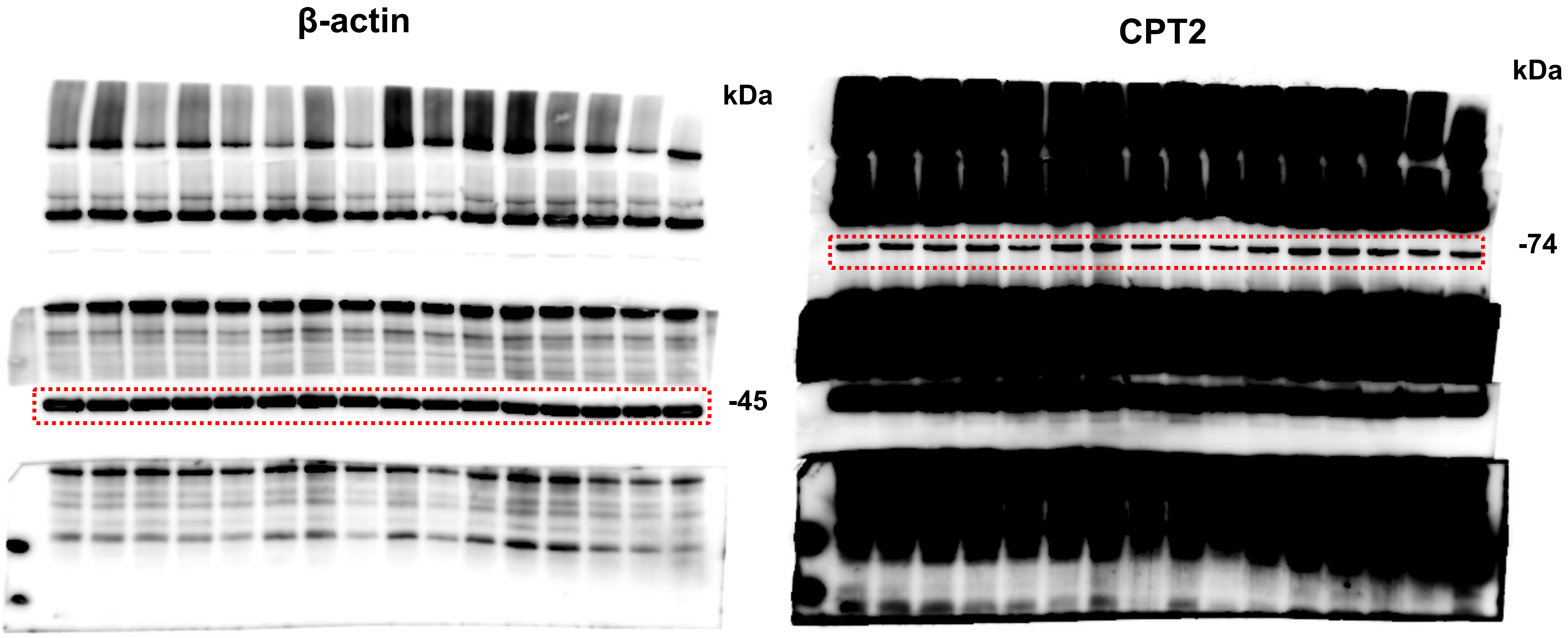

Note: 1. The same blot and different exposure times (short and long exposures shown) were used.
2. Bands used in Figures are indicated by boxes in red outlines.

Fig. S4D (PPARα-Full unedited blot)

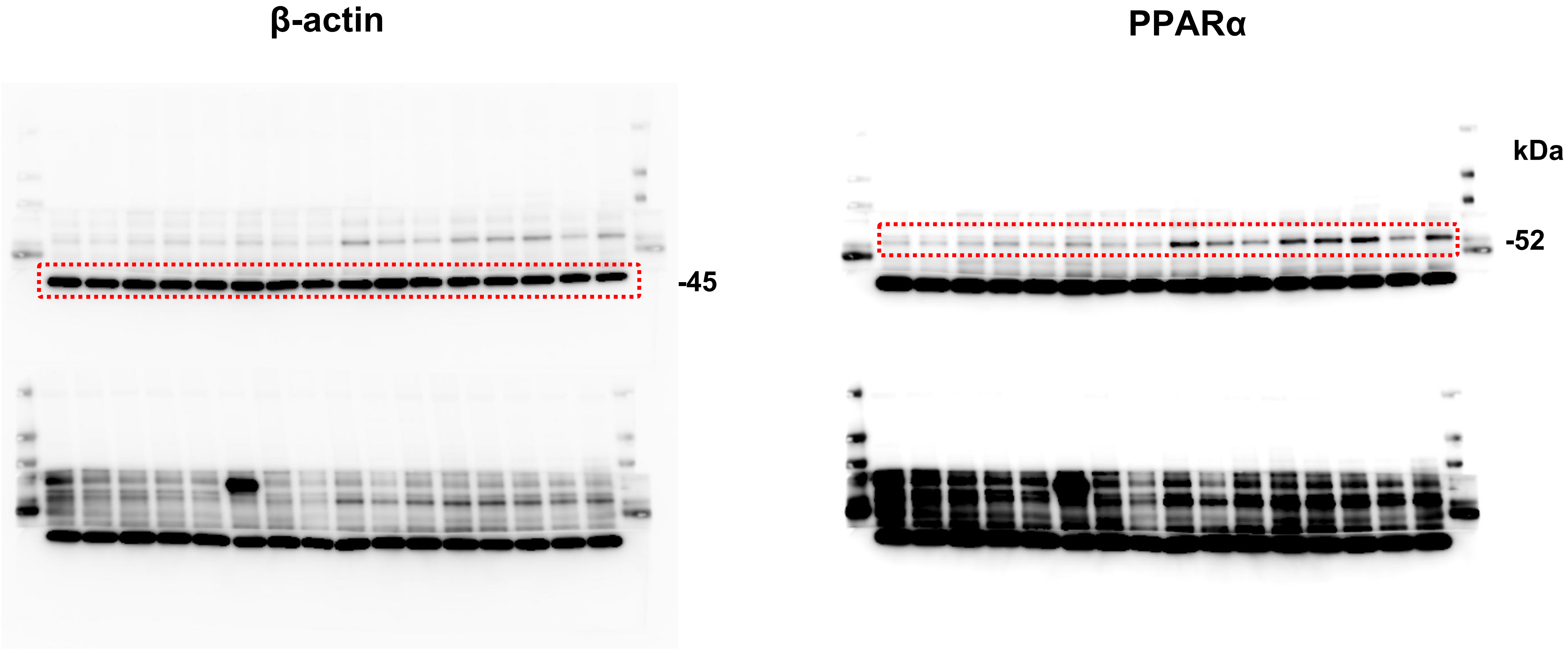

Note: 1. The same blot was used.
2. Bands used in Figures are indicated by boxes in red outlines.

Fig. S7D (GLUT2-Full unedited blot)

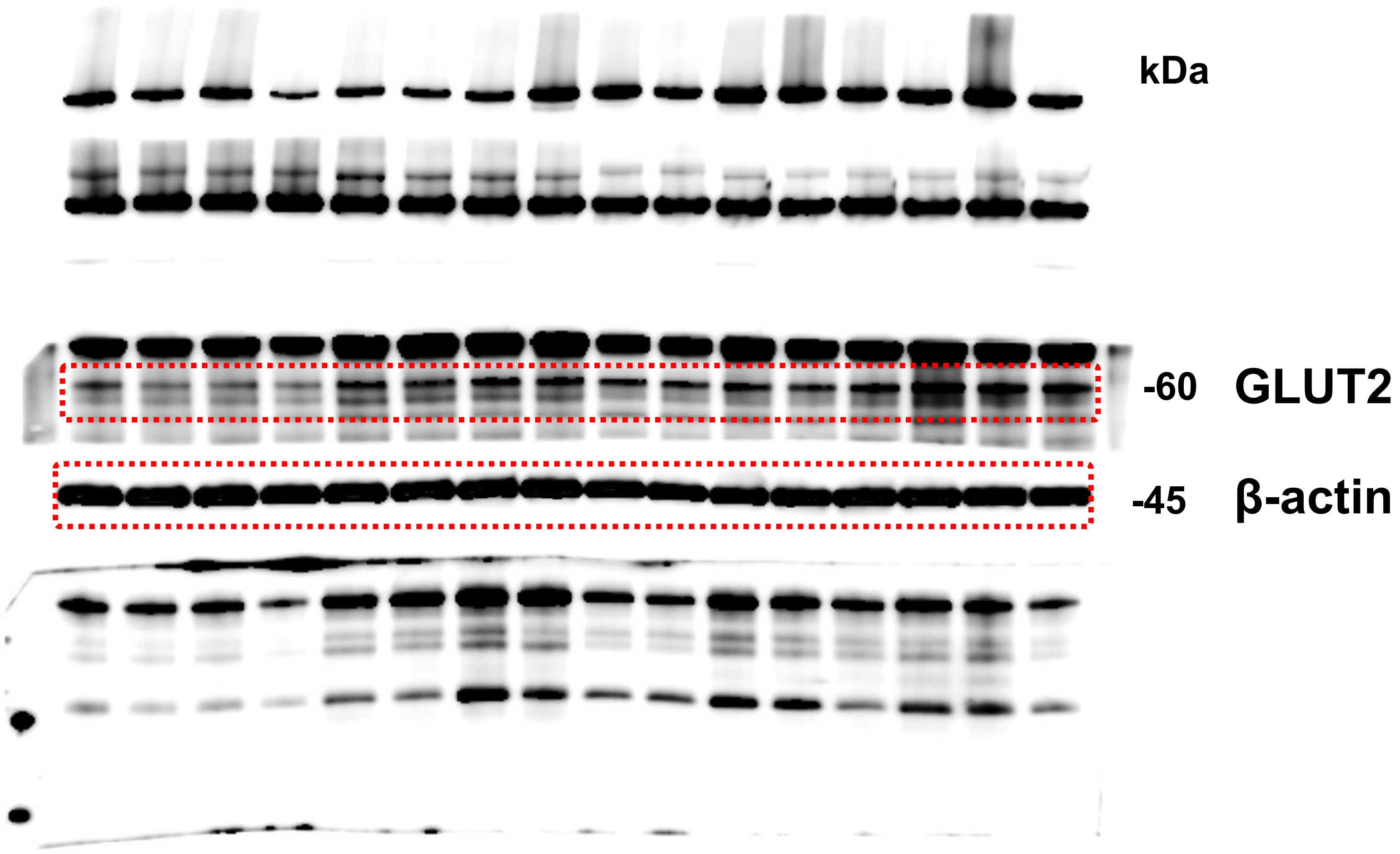

Note: 1. The same blot was used.
2. Bands used in Figures are indicated by boxes in red outlines.

Fig. S7D (GK-Full unedited blot)

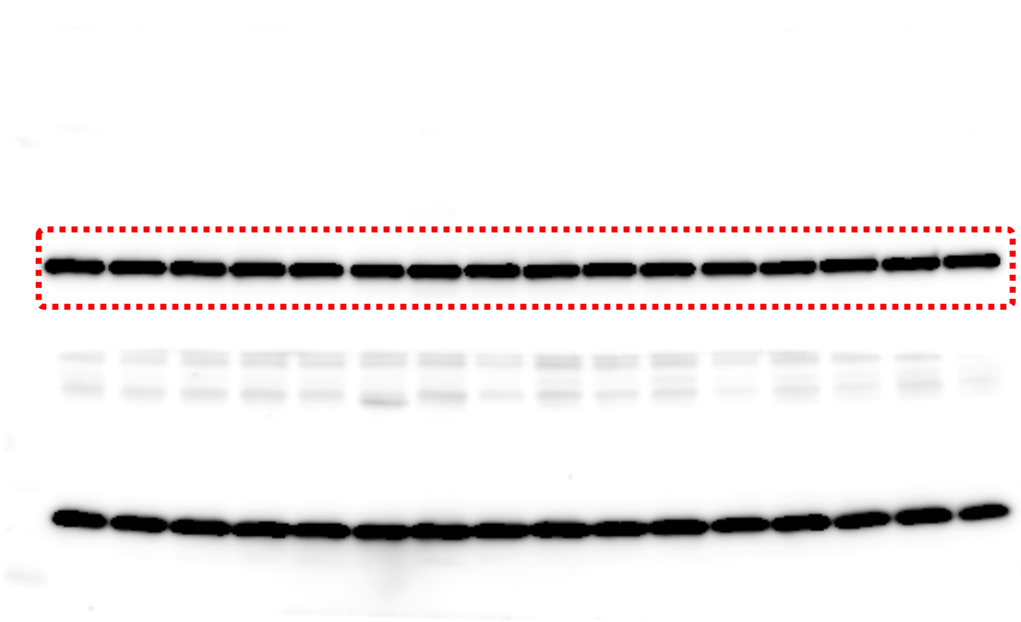

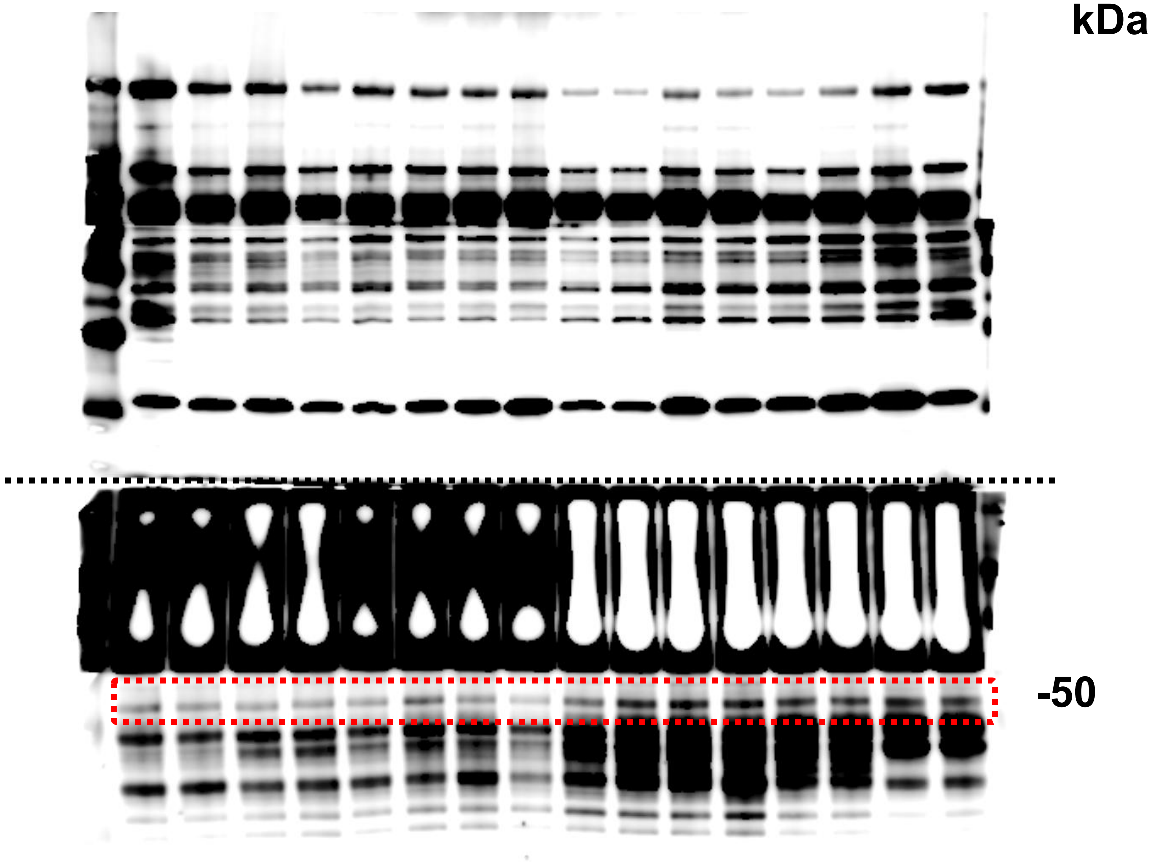

Note: 1. Separate, noncontiguous lanes from the same samples were used for each antibody.
2. Bands used in Figures are indicated by boxes in red outlines.

Fig. S7D (p-GS-GS-Full unedited blot)

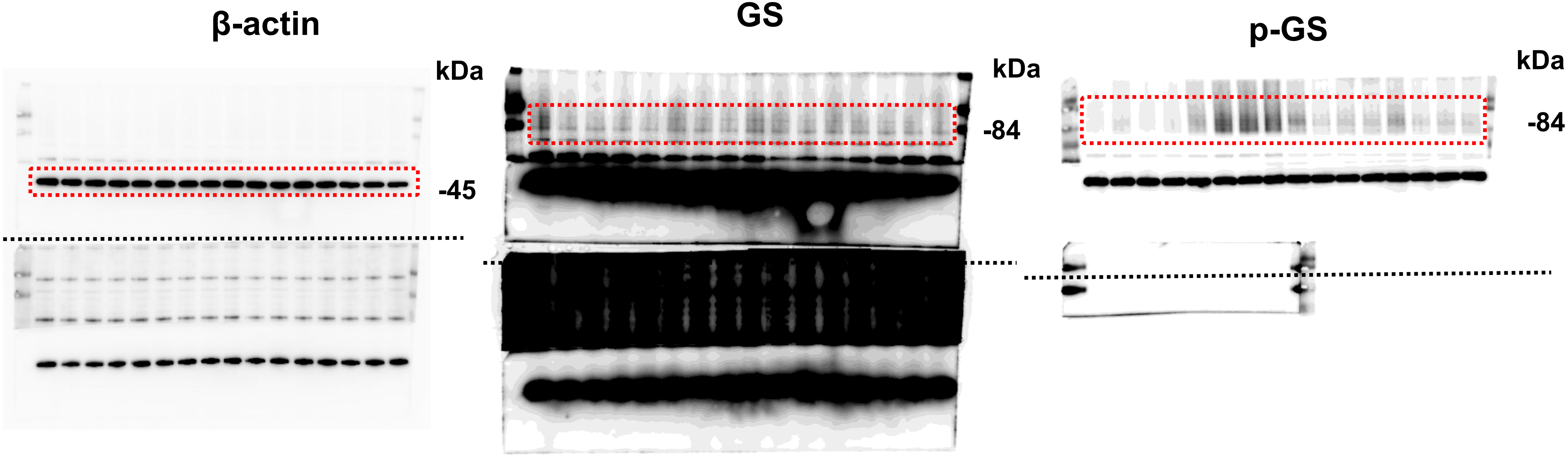

Note: 1. In GS and β-acti expression, the same blot and different exposure times (short and long exposures shown) were used. Between p-GS and GS expression, separate, noncontiguous lanes from the same samples were used for each antibody.
2. Bands used in Figures are indicated by boxes in red outlines.

Fig. S7D (p-GP-GP-Full unedited blot)

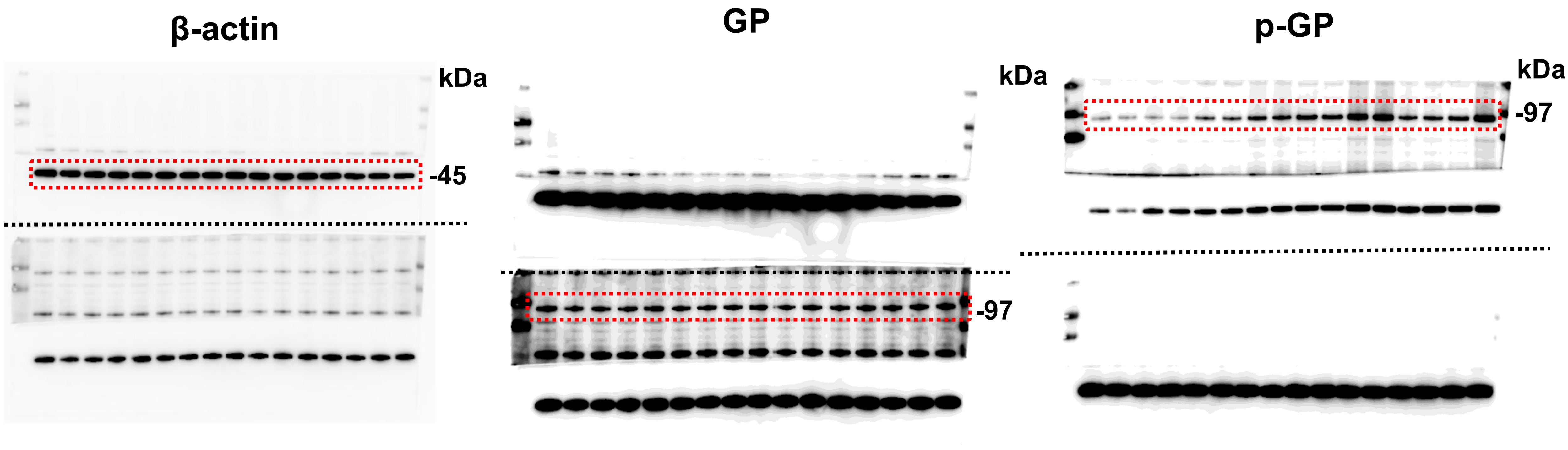

Note: 1. In GP and β-acti expression, the same blot and different exposure times (short and long exposures shown) were used. Between p-GP and GP expression, separate, noncontiguous lanes from the same samples were used for each antibody.
2. Bands used in Figures are indicated by boxes in red outlines.

Fig. S7D (PCK1-Full unedited blot)

β-actin

See Fig. S7D (GK-Full unedited blot)

PCK1

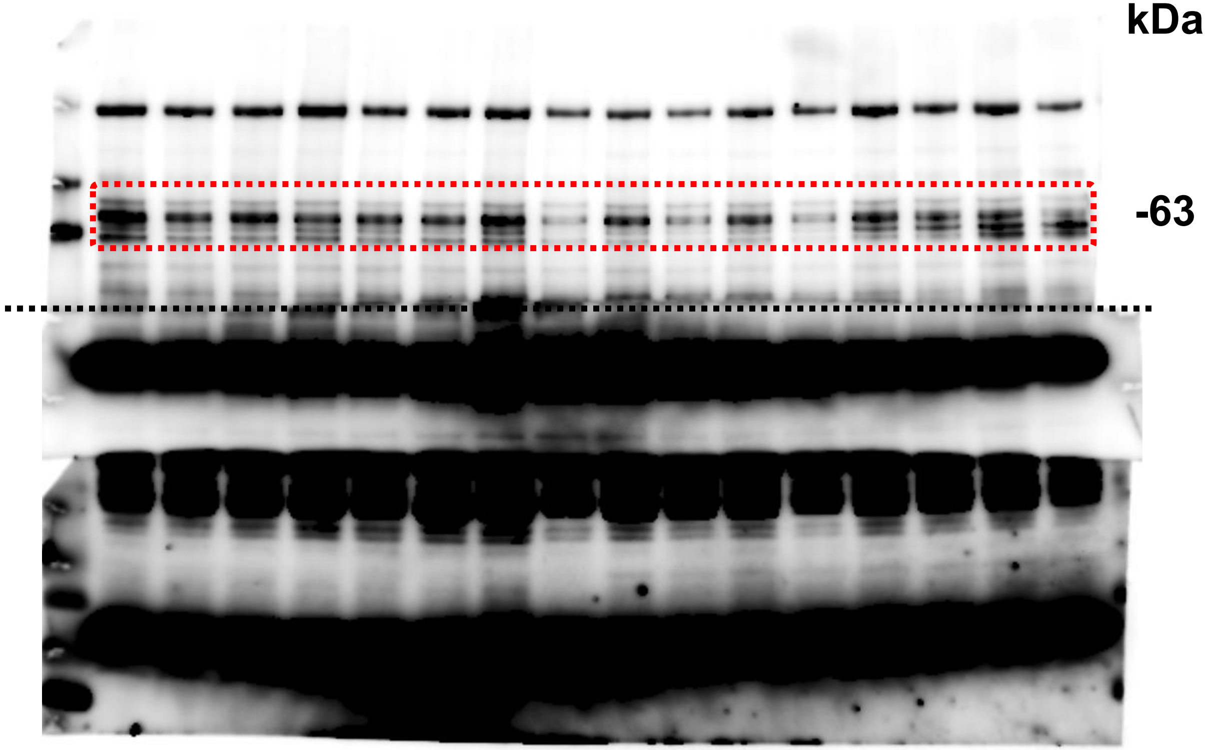

Note: 1. The same blot and different exposure times (short and long exposures shown) were used.
2. Bands used in Figures are indicated by boxes in red outlines.

Fig. S8F (SOD1-Full unedited blot)

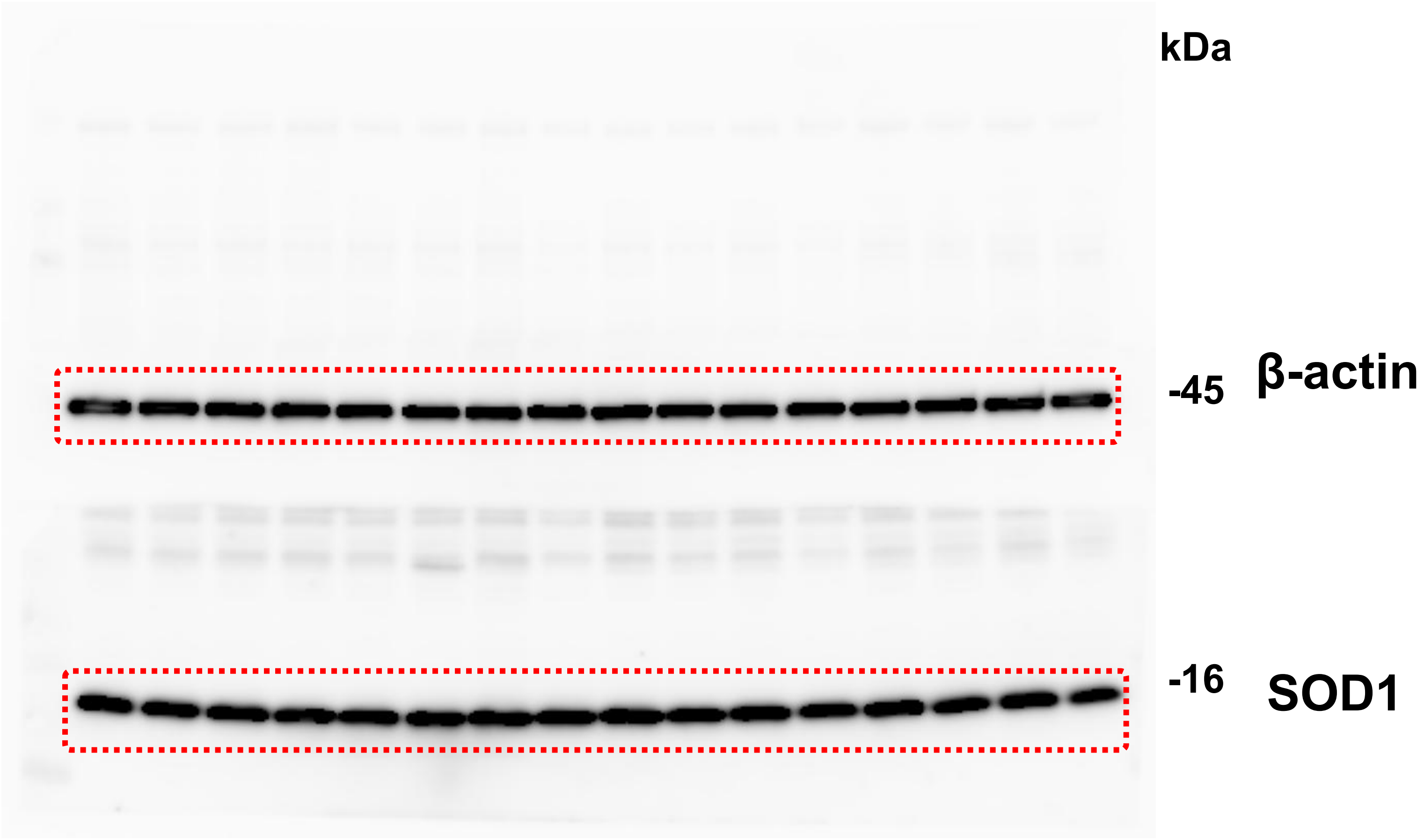

Note: 1. The same blot was used.
2. Bands used in Figures are indicated by boxes in red outlines.

Fig. S8F (SOD2-Full unedited blot)

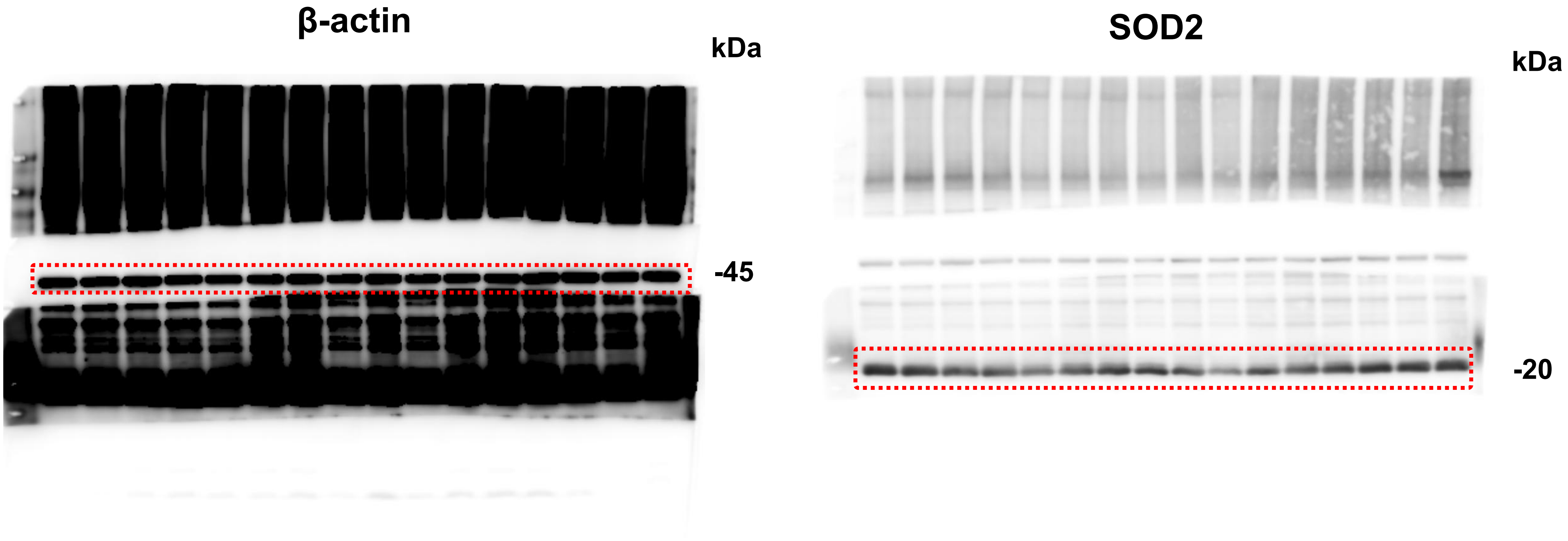

Note: 1. The same blot and different exposure times (short and long exposures shown) were used.
2. Bands used in Figures are indicated by boxes in red outlines.

Fig. S8F (CAT-Full unedited blot)

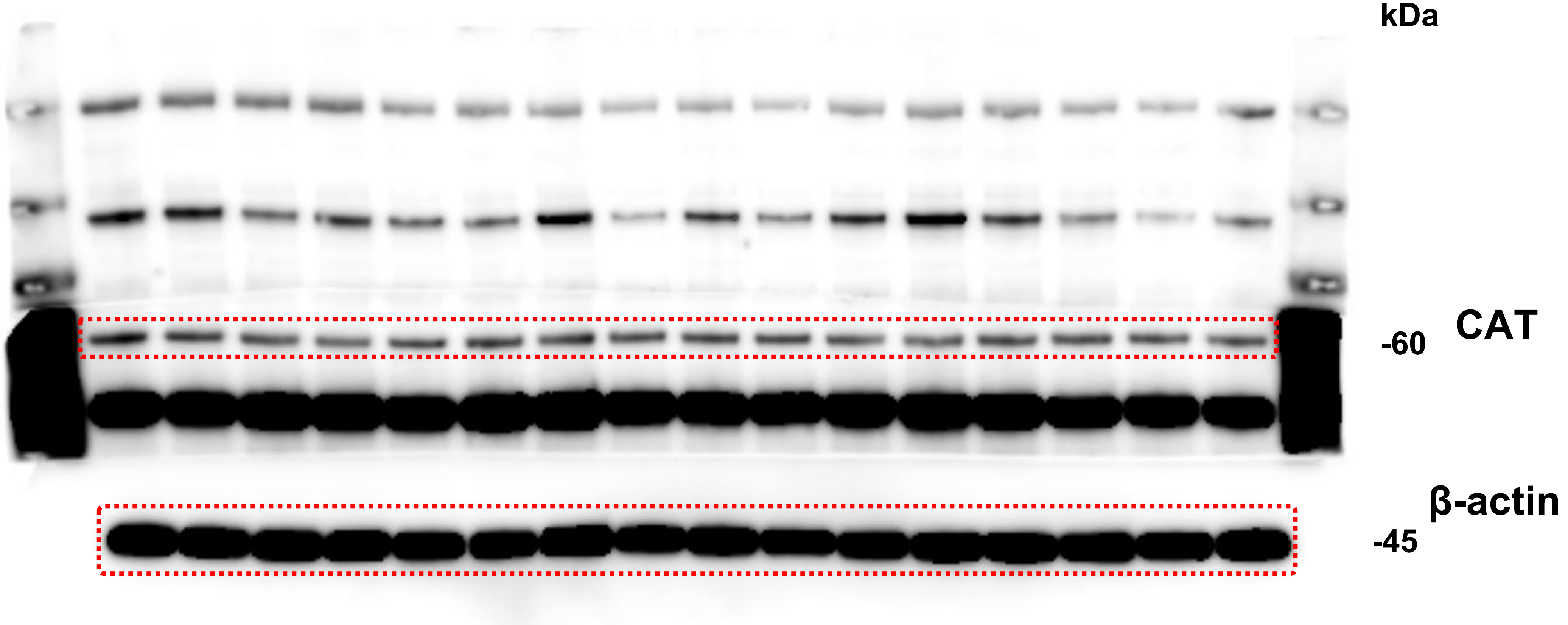

Note: 1. The same blot was used.
2. Bands used in Figures are indicated by boxes in red outlines.

Fig. S8F (GPX1-Full unedited blot)

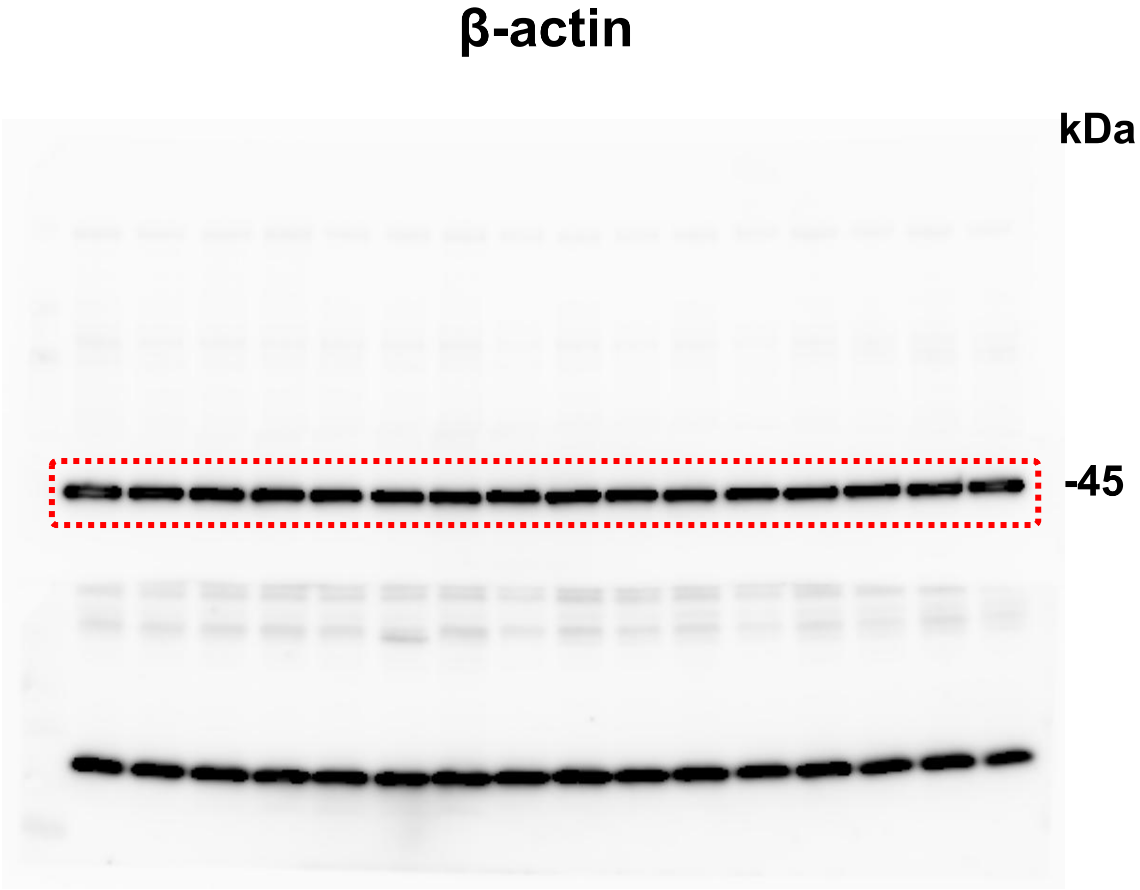

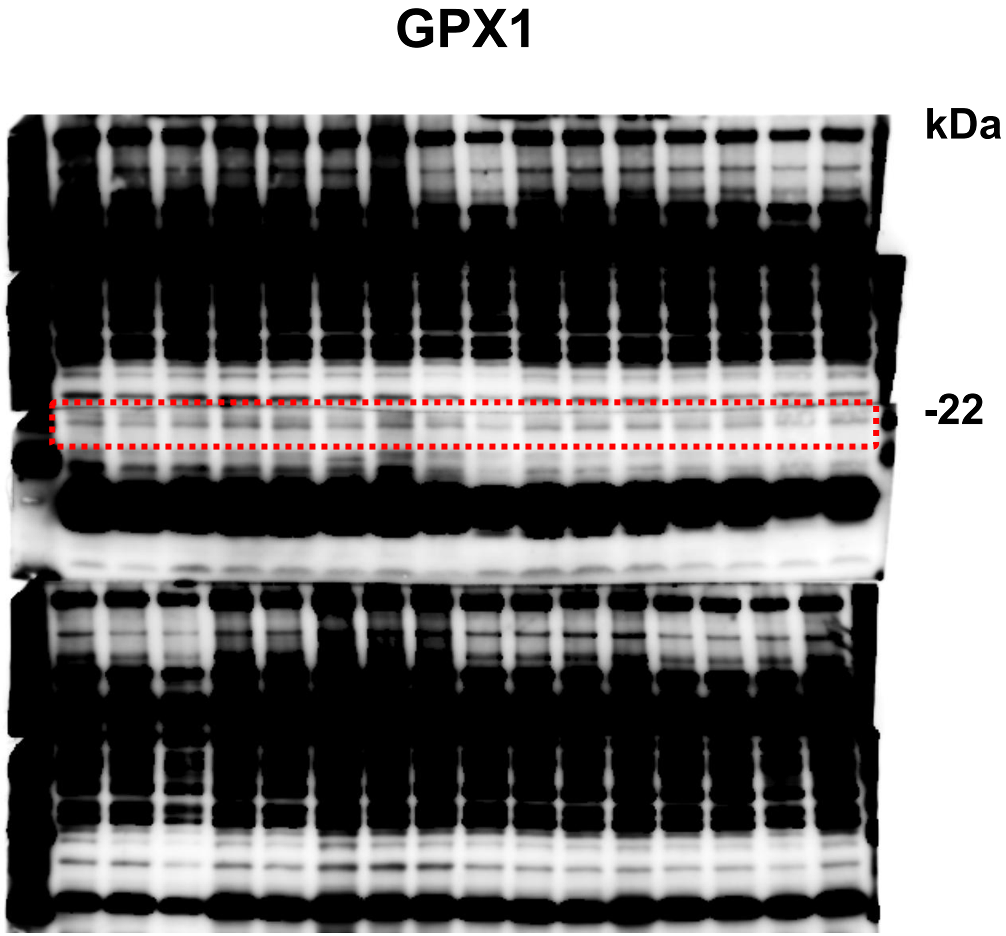

Note: 1. The same blot and different exposure times (short and long exposures shown) were used.
2. Bands used in Figures are indicated by boxes in red outlines.

Fig. S9F (Cleaved-caspase3/Caspase-3-Full unedited blot)

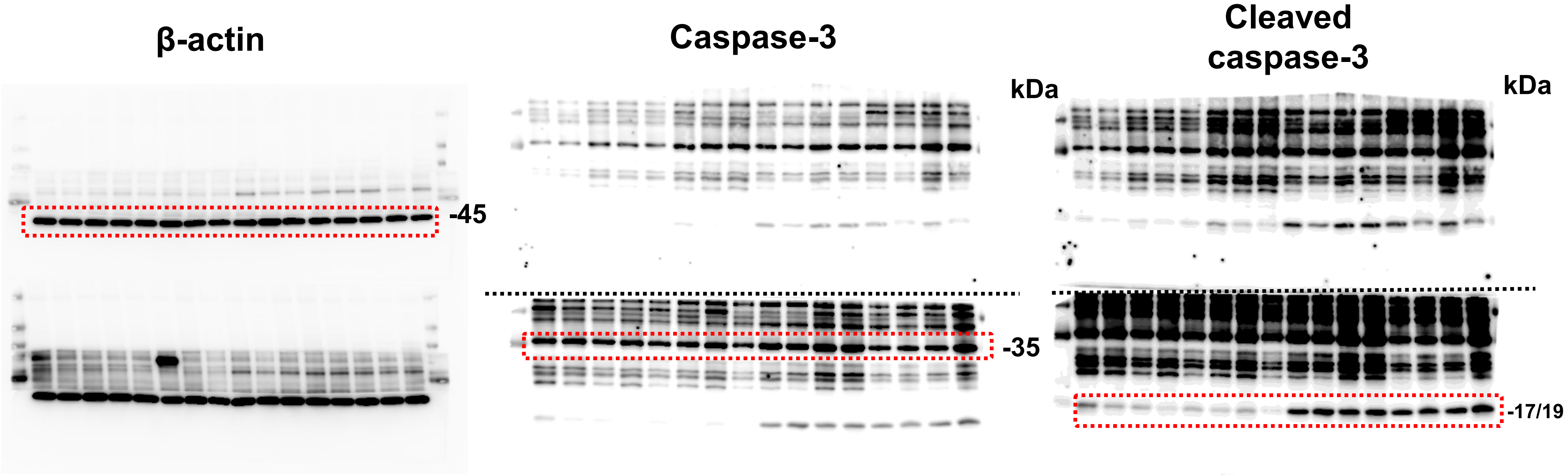

Note: 1. In Cleaved caspase-3 and Caspase-3 expression, the same blot and different exposure times (short and long exposures shown) were used. Between Caspase-3 and β-actin expression, separate, noncontiguous lanes from the same samples were used for each antibody.
2. Bands used in Figures are indicated by boxes in red outlines.

Fig. S9F (BAK-Full unedited blot)

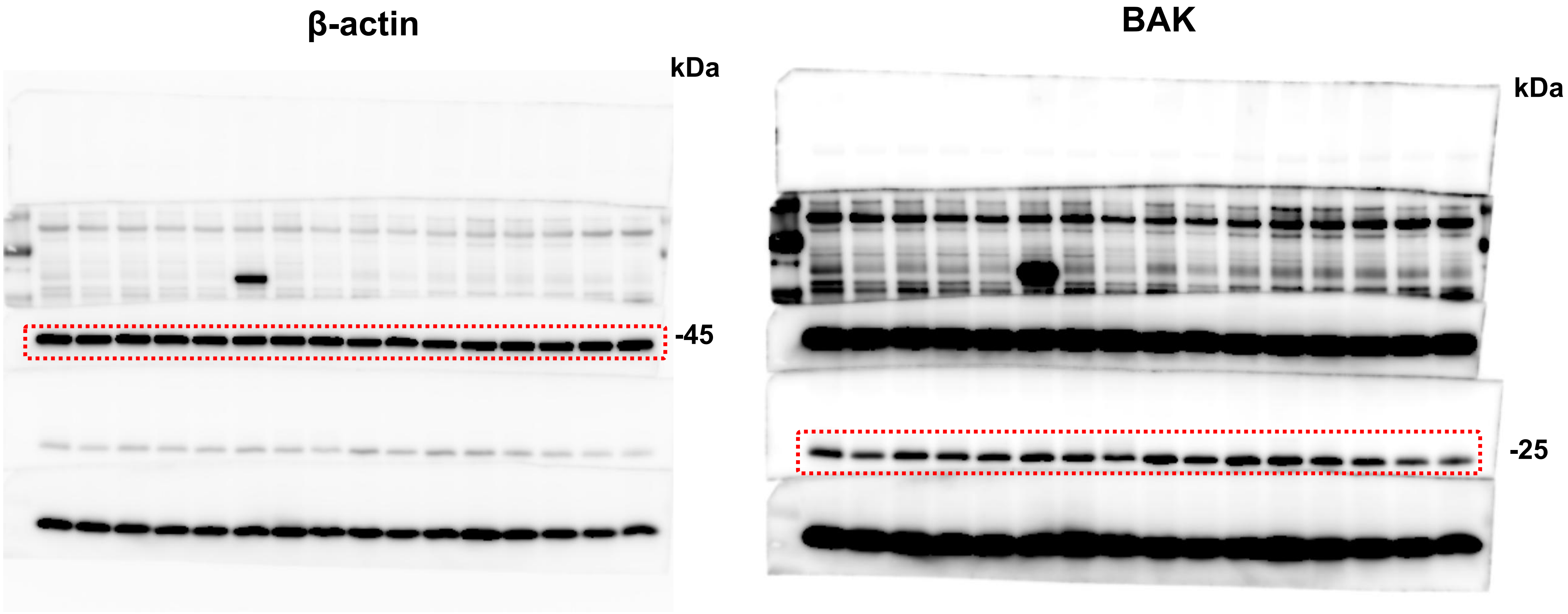

Note: 1. The same blot and different exposure times (short and long exposures shown) were used.
2. Bands used in Figures are indicated by boxes in red outlines.

Fig. S9F (BAX-Full unedited blot)

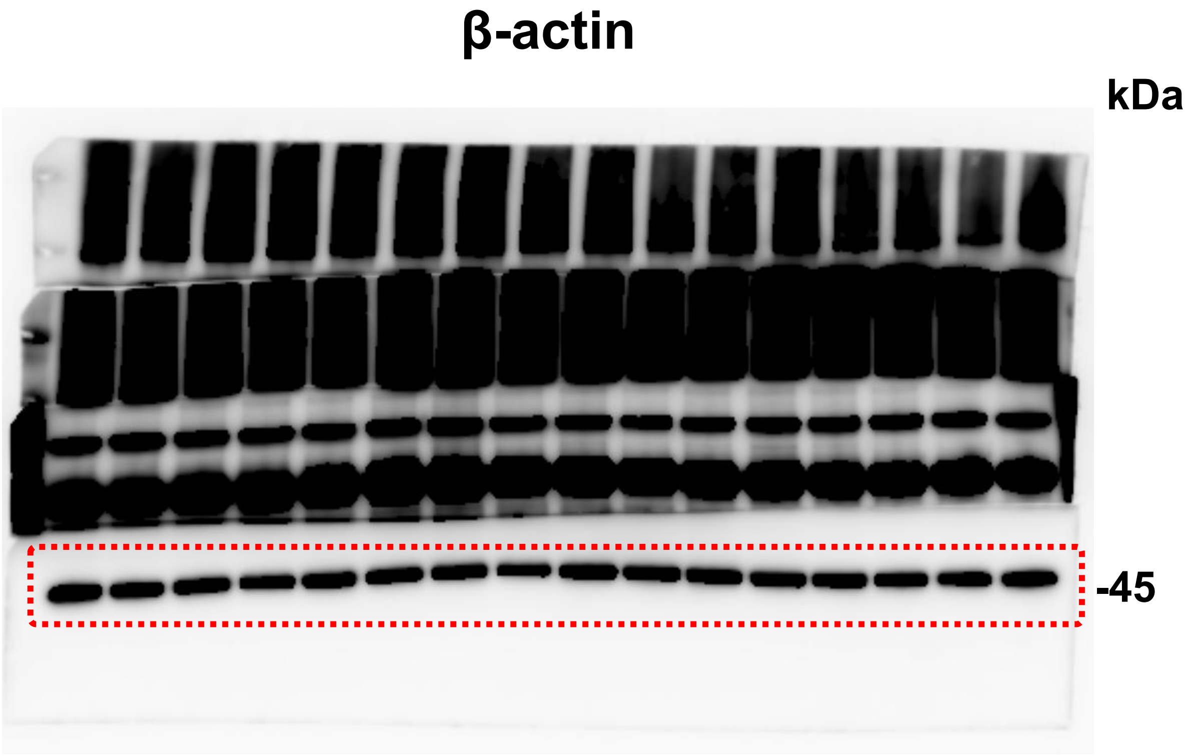

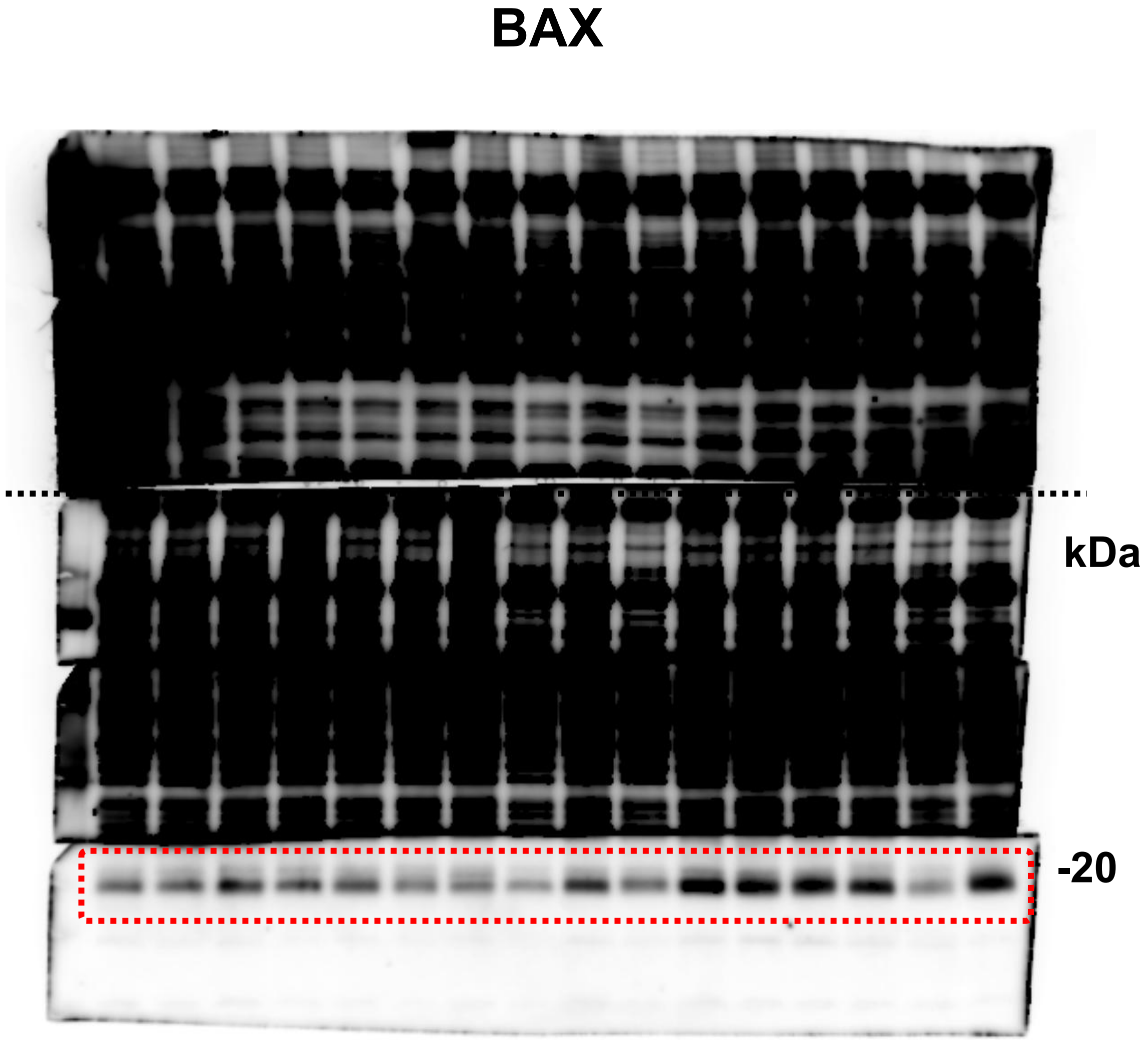

Note: 1. Separate, noncontiguous lanes from the same samples were used for each antibody.
2. Bands used in Figures are indicated by boxes in red outlines.

Fig. S9F (Cleaved-caspase-8/Caspase-8-Full unedited blot)

β-actin

See Fig. S9F (BAK-Full unedited blot)

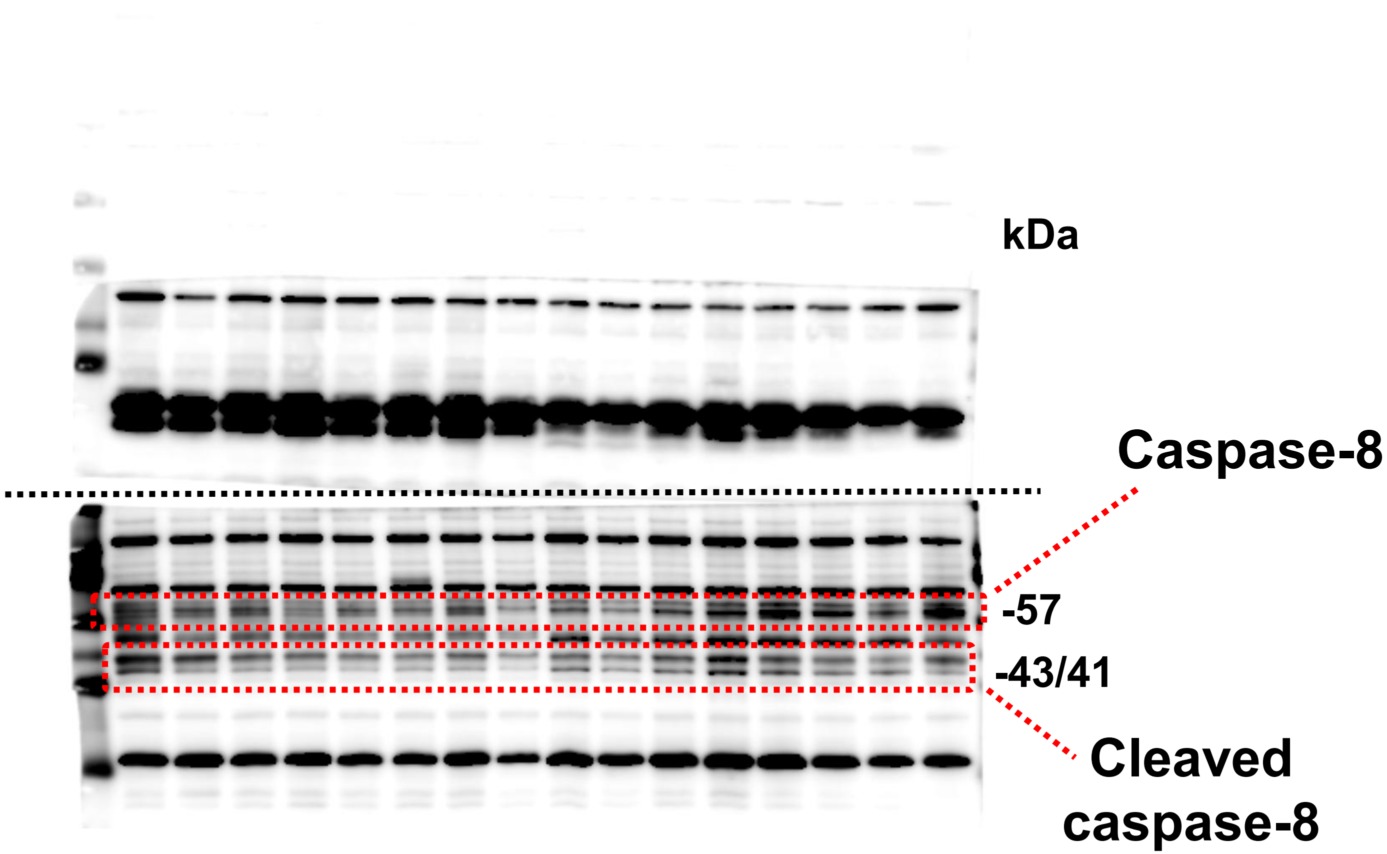

Note: 1. In Cleaved-caspase-8 and Caspase-8 expression, the same blot was used. Between Caspase-8 and β-actin expression, separate, noncontiguous lanes from the same samples were used for each antibody.
2. Bands used in Figures are indicated by boxes in red outlines.

Fig. S10 (LC3-Ⅱ/LC3-Ⅰ-Full unedited blot)

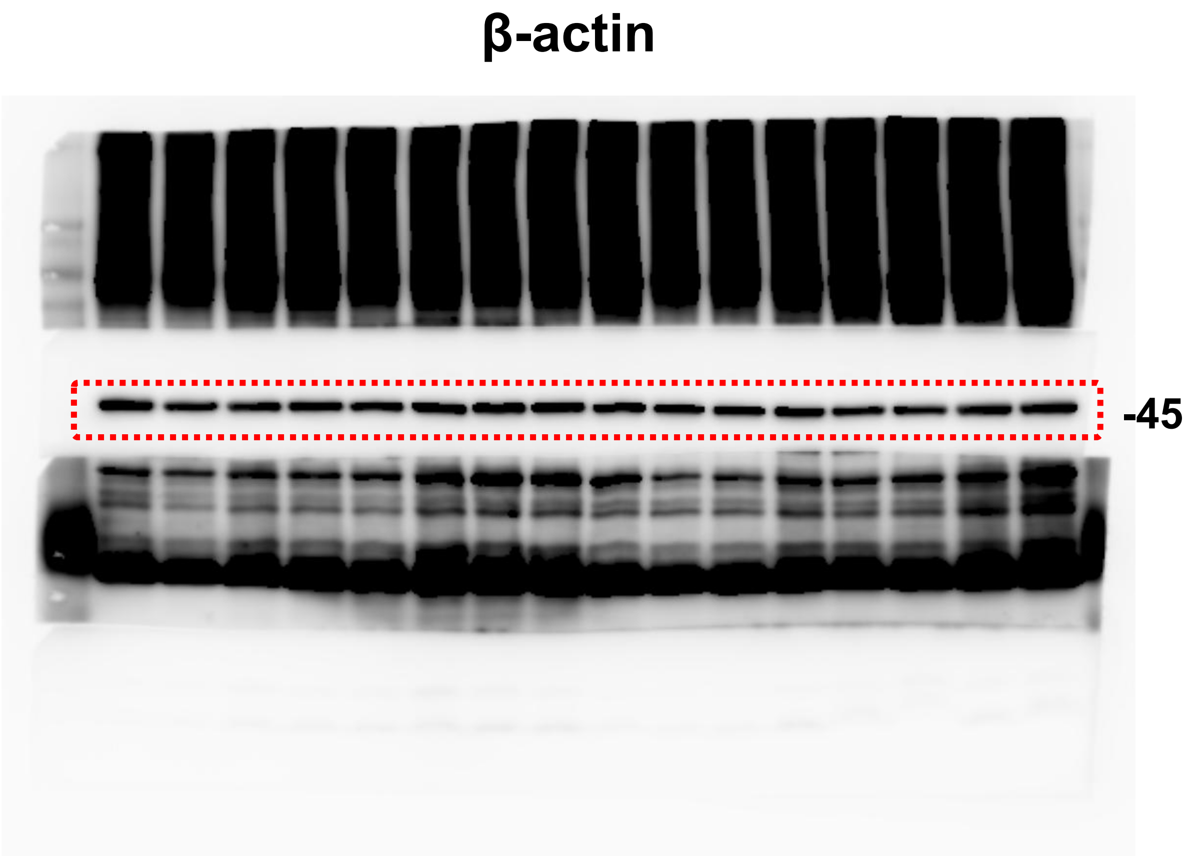

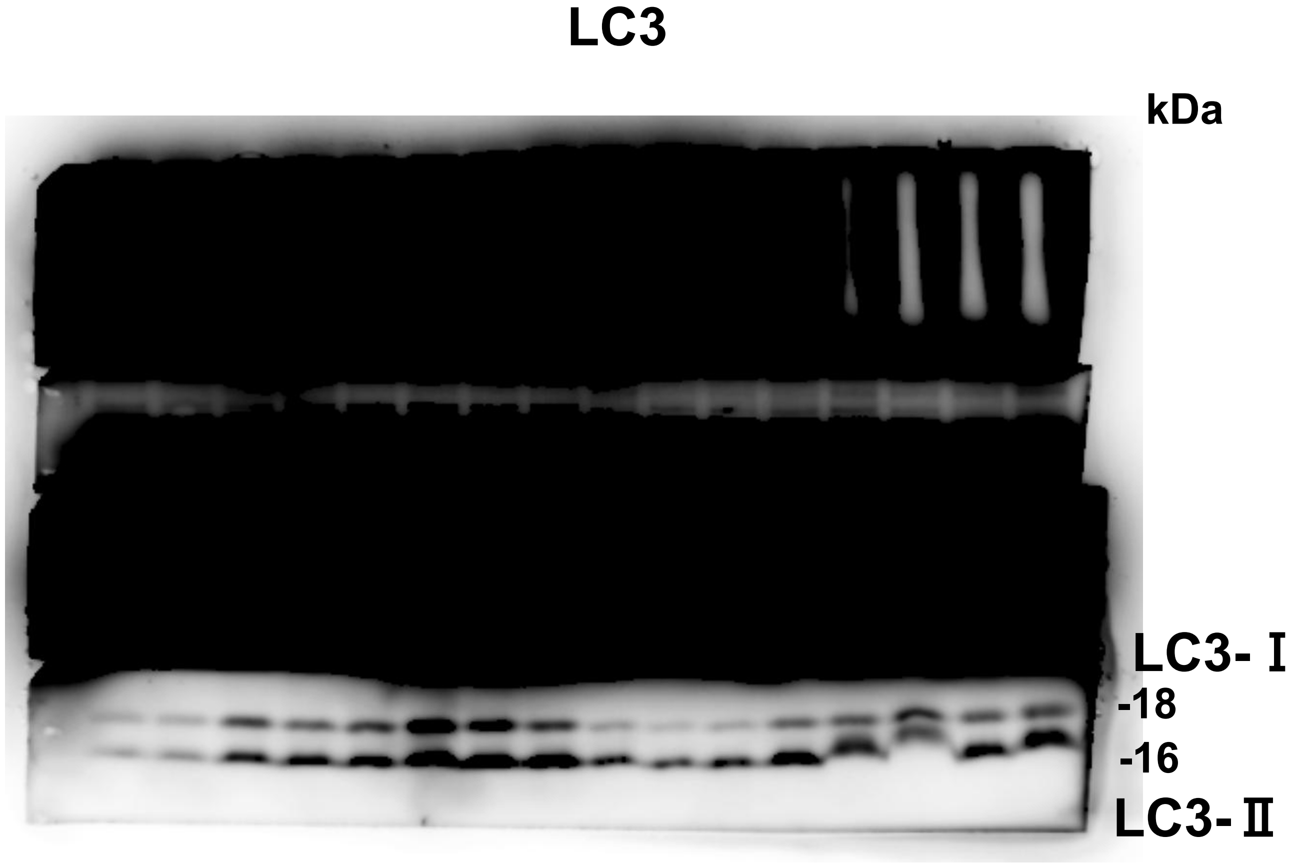

Note: 1. In LC3-Ⅱ and LC3-Ⅰ expression, the same blot was used. Between LC3 and β-actin expression, the same blot and different exposure times (short and long exposures shown) were used.
2. Bands used in Figures are indicated by boxes in red outlines.

Fig. S10 (p62-Full unedited blot)

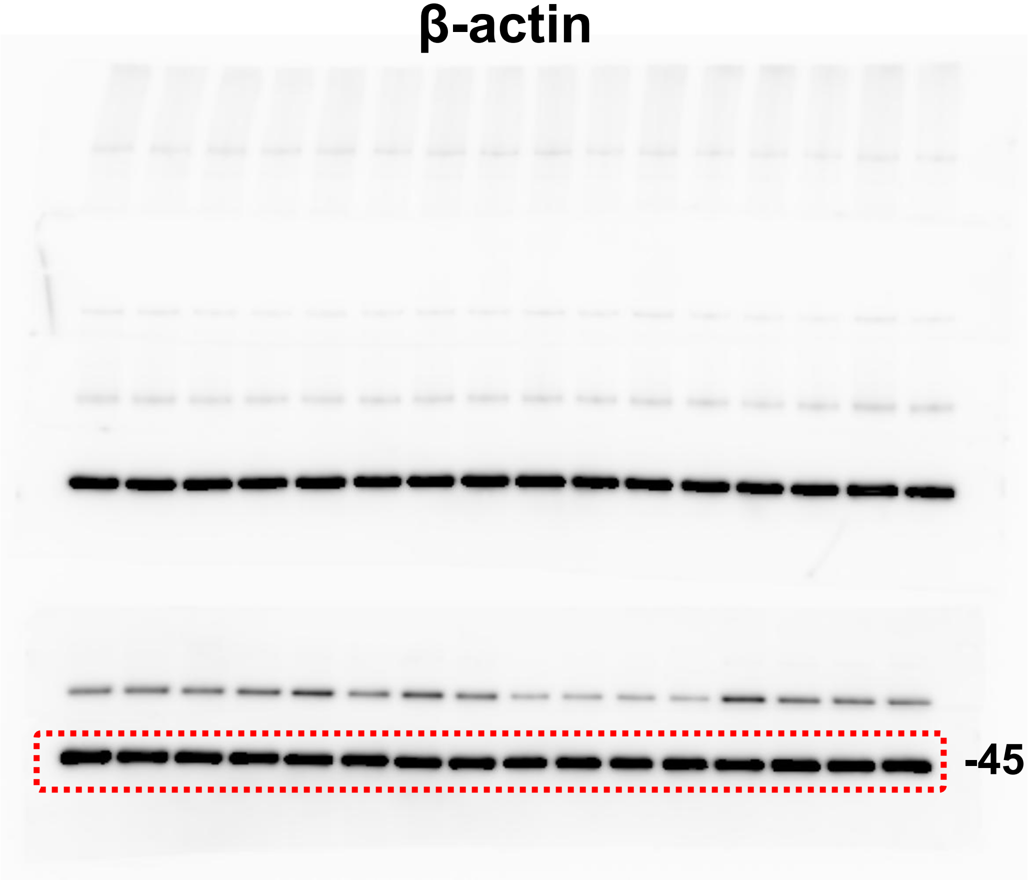

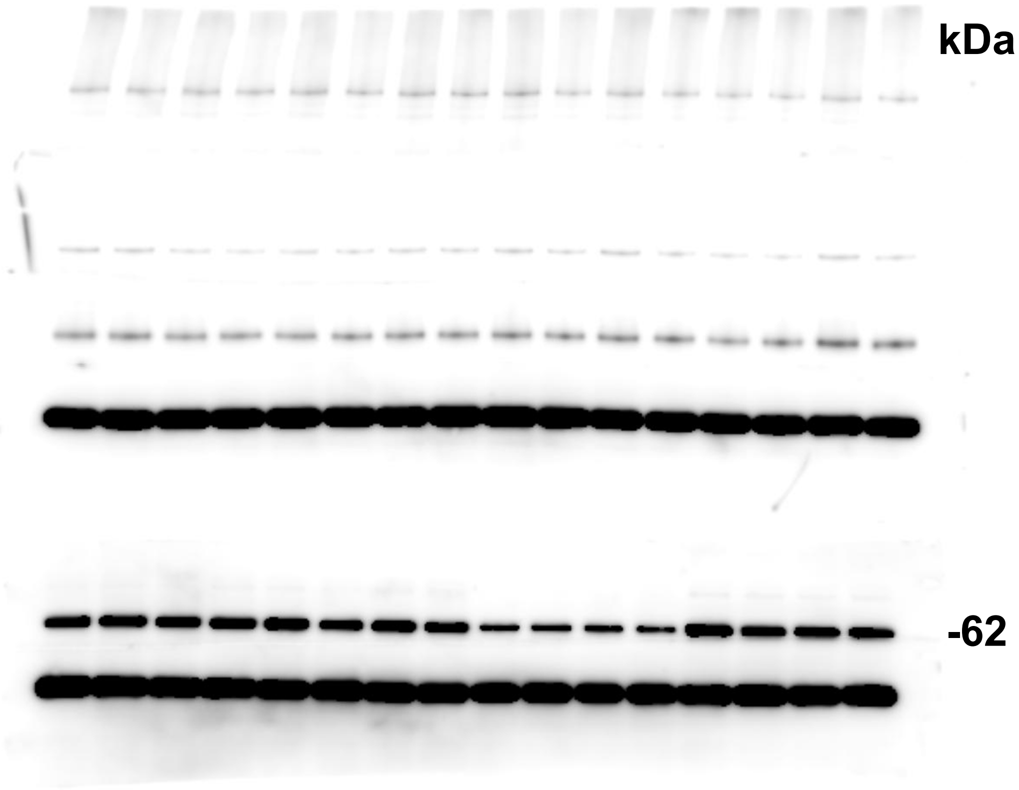

Note: 1. The same blot and different exposure times (short and long exposures shown) were used.
2. Bands used in Figures are indicated by boxes in red outlines.
